# Collaborative analysis of multi-gigapixel imaging data using Cytomine

**DOI:** 10.1093/bioinformatics/btw013

**Published:** 2016-01-10

**Authors:** Raphaël Marée, Loïc Rollus, Benjamin Stévens, Renaud Hoyoux, Gilles Louppe, Rémy Vandaele, Jean-Michel Begon, Philipp Kainz, Pierre Geurts, Louis Wehenkel

**Affiliations:** ^1^Systems and Modeling, Department of Electrical Engineering and Computer Science and GIGA-Research, University of Liège, Liège, Belgium; ^2^Bioimage Analysis Unit, Institut Pasteur, Paris, France; ^3^Institute of Biophysics, Medical University of Graz, Graz, Austria

## Abstract

**Motivation**: Collaborative analysis of massive imaging datasets is essential to enable scientific discoveries.

**Results:** We developed Cytomine to foster active and distributed collaboration of multidisciplinary teams for large-scale image-based studies. It uses web development methodologies and machine learning in order to readily organize, explore, share and analyze (semantically and quantitatively) multi-gigapixel imaging data over the internet. We illustrate how it has been used in several biomedical applications.

**Availability and implementation:** Cytomine (http://www.cytomine.be/) is freely available under an open-source license from http://github.com/cytomine/. A documentation wiki (http://doc.cytomine.be) and a demo server (http://demo.cytomine.be) are also available.

**Contact:** info@cytomine.be

**Supplementary information:** Supplementary data are available at *Bioinformatics* online.

## 1 Introduction

In various scientific domains (incl. biology, biomedicine, astronomy, botany, geology, paleobiology, marine research, aerobiology, climatology), projects leading to terabytes of multi-gigapixel images become increasingly common (The data deluge, 2012) e.g. biomedical research studies often rely on whole-slide virtual microscopy or automated volume electron microscopy. In these fields, significant advances could be made by multidisciplinary collaboration involving distributed groups of life scientists and computer scientists exploiting large-scale image networks (Moody *et al.*, 2013; Poldrack, 2014), or eventually by enlisting the help of members of the general public in large imaging surveys (Clery, 2011) through interactive games (e.g. EyeWire (http://eyewire.org/) and Brainflight (http://brainflight.org/) projects). For example, researchers in experimental histology are willing to precisely annotate images and need to consult distant experts in pathology or molecular biology. Developers of image processing algorithms are willing to collaborate with machine learning specialists to build complementary image analysis workflows. Furthermore, all these individuals need to actively collaborate to gain new insights, e.g. computer scientists require realistic ground truth and proofreadings (Ground-truth data cannot do it alone, 2011) provided by life scientists to design and refine their analysis methods. Vice versa, life scientists increasingly rely on algorithms or crowdsourced outputs in combination with proofreading tools to enable efficient analysis of very large image sets.

Bioimage informatics aims at developing software to ease the analysis of large-scale biomaging data (Myers, 2012). In recent years, several software have been developed including CellProfiler (Carpenter *et al.*, 2006), CATMAID (Saalfeld *et al.*, 2009), BisQue (Kvilekval *et al.*, 2010), ilastik (Sommer *et al.*, 2011), Icy (de Chaumont *et al.*, 2012), Fiji (Schindelin *et al.*, 2012), OMERO (Allan *et al.*, 2012) and BigDataViewer (Pietzsch *et al.*, 2015). Applications and extensions of these software packages have been proposed in various research fields (e.g. in the context of Drosophila (Jug *et al.*, 2014) and Zebrafish (Mikut *et al.*, 2013) research, or in plant sciences (Lobet *et al.*, 2013)) to address rather specific biological questions (e.g. to map neuronal circuitry in Schneider-Mizell *et al.*, 2015).

In this work, we present Cytomine, a novel open-source, rich web environment to enable highly collaborative analysis of multi-gigapixel imaging data. This tool has been designed with the following objectives in mind:

- provide remote and collaborative principles,
- rely on data models that allow to easily organize and semantically annotate imaging datasets in a standardized way,
- efficiently support high-resolution multi-gigapixel images,
- provide mechanisms to readily proofread (Ground-truth data cannot do it alone, 2011) and share image quantifications produced by machine learning-based image recognition algorithms (de Souza, 2013; Murphy, 2011).

**Fig. 1.**
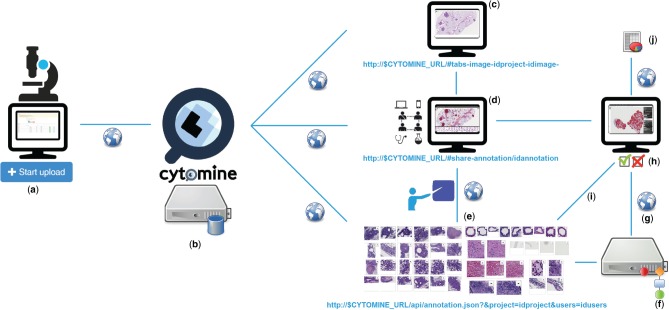
Overview of multidisciplinary collaborative principles illustrated for tumor segmentation in H&E lung cancer whole tissue slides: (**a**) Images are uploaded using Cytomine-WebUI or remote clients. (**b**) Images and related data are stored by Cytomine-Core and Cytomine-Image Management System. (**c**) Once uploaded, multi-gigapixel images are de facto available to other distributed users according to access rights and referenced by URLs. (**d**) Remote, multidisciplinary individuals are collaboratively and semantically annotating regions of interest in images and each annotation is referenced by its URL. (**e**) Expert annotations can be filtered and sets of annotations can be displayed or retrieved through the API. (**f**) Distributed algorithms can exploit these annotations, here a segmentation recognition model is built by supervised learning based on expert training examples. (**g**) An algorithm or recognition model can be applied remotely on new multi-gigapixel images for automatic annotation. (**h**) Experts review other user and automatic annotations by using Cytomine-WebUI proofreading tools. (**i**) Reviewed annotations can eventually be reused to refine and re-apply the recognition model. (**j**) Once image annotations are validated by an expert, final quantification results of the ‘reviewed layer’ are exported in standard formats

While some of these features are available in existing tools, none of these tools provide all these features simultaneously. By emphasizing collaborative principles, our aim with Cytomine is to accelerate scientific progress and to significantly promote image data accessibility and reusability (The data deluge, 2012; Moody *et al.*, 2013; Poldrack, 2014). We want to break common practices in this domain where imaging datasets, quantification results and associated knowledge are still often stored and analyzed within the restricted circle of a specific laboratory. To achieve this goal, the Cytomine platform permits active collaboration between distributed groups of life scientists, computer scientists and citizen scientists. It allows seamless online sharing and reviewing of semantic and quantitative information associated with large images, either produced manually or automatically using machine learning algorithms, as schematically illustrated in Figure 1.

The paper is structured as follows. In Section 2 we describe the main design principles and functionalities of Cytomine. In Section 3, we briefly present use cases initiated by our collaborators to help readers to determine how they can use our software to address their own research questions. We then discuss the concepts of extensibility of the platform in Section 4, and finally, we conclude.

## 2 System and methods

To allow image-based collaborative studies and meet software efficiency and usability criteria (Software with impact, 2014; Carpenter *et al.*, 2012; Prins *et al.*, 2015), the software is decomposed into four main components (Supplementary Note 1) communicating through web mechanisms (through a RESTful API): Cytomine core (Cytomine-Core), Cytomine Image Management System (Cytomine-IMS), Cytomine web user interface (Cytomine-WebUI) and Cytomine analysis modules (Cytomine-DataMining), designed as follows.

### 2.1 Cytomine-core

Cytomine-Core relies on recent web and database software development technologies. Its underlying data models (Supplementary Note 2) allow to create and store projects. Each project can be accessed by multiple users through authentication. A project can contain multi-gigapixel image sequences and a user-defined ontology, i.e. a structured list of domain-specific semantic terms. Each image instance can be annotated by users or software using annotation objects of various shapes for regions of interest (e.g. a cell or a tissue subregion) and labeled with one or multiple semantic terms from the ontology (e.g. a specific cell type or tissue structure). In addition, metadata (key-value properties, associated files and rich descriptions) can be associated to any project, image and annotation. Such data can be created remotely either by human experts (through Cytomine-WebUI) or automatically (by our analysis modules or any third-party software implementing basic web communication mechanisms). Because these data are identified by URLs they are de facto shared with any authenticated user. Also, as they are represented in standard formats (namely JSON, a lightweight data-interchange format), they can be automatically parsed and generated by registered external applications.

### 2.2 Cytomine-IMS

Cytomine-IMS backend server provides web services that encapsulate a collection of distributed, specialized image server instances. It is used to upload 5D image sequences (x,y,z,c,t planes) and to dynamically deliver original image areas and annotation masks over the internet – at any pyramid resolution. It supports various standards and specific microscopy image formats (including most of whole-slide scanner formats) either by directly accessing their native formats, or by seamlessl conversion to a pyramidal format during the upload phase (see Supplementary Note 1 for a list of supported formats).

### 2.3 Cytomine-WebUI

**Fig. 2.**
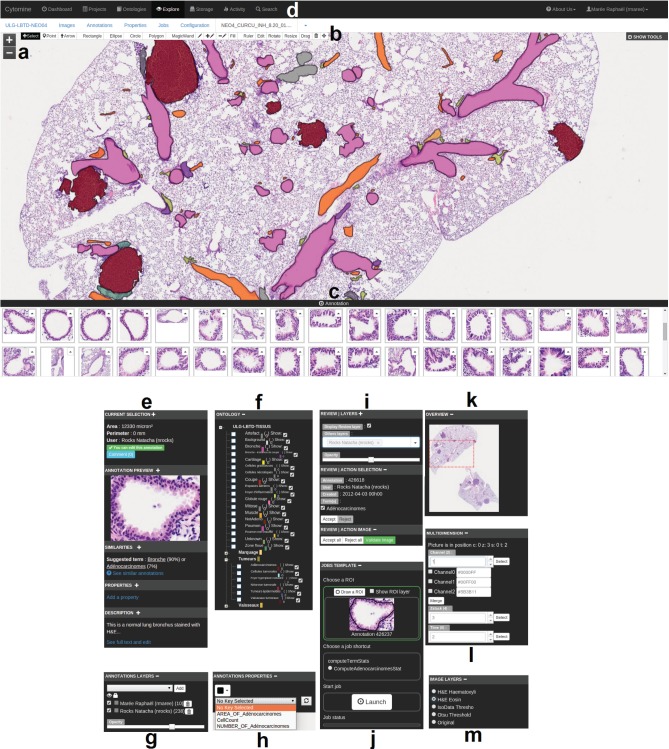
Overview of Cytomine-WebUI: (**a**) Zoomable multi-gigapixel image viewer (a la Google Maps) with overlaid annotations colored according to ontology terms (Original image size: 19968 × 25088 pixels). (**b**) Annotation drawing tools including various shapes and operations on polygons. (**c**) Gallery of bronchus annotations in current image. (**d**) Main menu including project listing, ontology editor, storage to upload images, user activity statistics, textual search engine. (**e**) Selected annotation panel with thumbnail, suggested terms (based on content-based image retrieval algorithm), textual description. (**f**) Project-specific, user-defined ontology for semantic annotation. (**g**) Activation of annotation layers of possibly distributed users and softwares. (**h**) Annotation properties (key-value pairs). (**i**) Proofreading tools to accept or edit annotations. (**j**) Job template panel to launch pre-configured processing routines on regions of interest. (**k**) Gigapixel image overview with current position. (**l**) Multidimensional image panel with selectors for channel, slice in a *z*-stack, and time point. (**m**) Image layer panel to apply on-the-fly tile image processing

Cytomine-WebUI is a customizable and responsive rich internet application (Fig. 2), accessible through regular web browsers and mobile devices. It allows to create, organize, visualize and edit all data. It includes a zoomable, tile-based viewer for multi-gigapixel images with the visualization of overlaid (human or computer-generated) annotation layers and their properties. Furthermore, an ontology editor, several modules to derive annotation statistics and visualize annotation galleries, a textual search engine and proofreading tools for expert reviewing of annotation objects are part of this user interface. In addition, we have implemented functionalities to allow various forms of collaborative works. One of them is the tracking of all user activities to e.g. allow multiple users to follow remotely another user’s observation paths and actions. Conversely, a blinded mode can be activated to hide image and user information to allow independent studies and reduce bias when analyzing imaging data. An additional module (Cytomine-IRIS, the interobserver reliability study module) also allows independent ground-truth construction and inter-observer annotation statistics e.g. to identify cell type classification disagreements among experts.

### 2.4 Cytomine-DataMining

Cytomine-DataMining analysis modules currently include variants of machine learning based image recognition algorithms (Marée *et al.*, 2013a) that can be run on remote servers (Supplementary Note 3). This property facilitates large-scale analysis on distributed cluster systems where expensive computations can be outsourced. We provide an unsupervised, incremental, content-based image retrieval method that searches on-the-fly for visually similar annotations in the database and displays them in Cytomine-WebUI every time a user draws an annotation (see examples in Supplementary Note 4.2). Variants of supervised image recognition algorithms are also provided for object classification, semantic segmentation and landmark detection (see examples in Supplementary Note 4.2). Through web communication mechanisms, these analysis modules can be launched from Cytomine-WebUI. These modules typically retrieve filtered sets of labeled annotation objects through the API and build computational image recognition models. These models can be applied at any pyramid level of a gigapixel image in order to analyze its content at different resolutions and automatically create novel annotation objects (e.g. cell or tumor geometries and their semantic terms for cell sorting and tumor quantification, or coordinates of points corresponding to landmarks for morphological measurements). Despite progress in machine learning, it often remains necessary for experts to proofread automatically generated annotations. For this purpose, we also provide Web UIs to revise computer-generated annotations (e.g. edit their shape or spatial localization, modify their ontology term,…). Notably, these editing tools are independent of our image recognition algorithms and can be used to remotely review annotation objects created by other software (see Supplementary Note 5.3 for details on extensibility) or scientists. Reviewed annotations are stored as novel entities in the database so they can be disseminated or used later to refine recognition models.

## 3 Applications

**Fig. 3.**
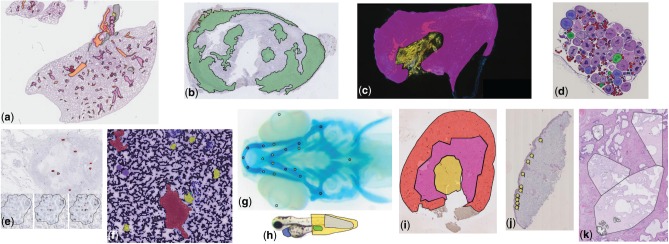
Examples of annotations created using Cytomine in images from various research fields (see Section 3 for additional details): (**a**) Delineation of tissue components in H&E images in mice lung cancer research (D.Cataldo’s lab), (**b**) Tumoral areas in HDAB images in mice lung cancer research (P. Martinive’s lab), (**c**) Area quantification in immunofluorescent mouse ear sponge assays in tumor angiogenesis (C. Gilles’ lab), (**d**) Counting of oocytes in H&E images in *Chondrostoma nasus* sexual maturation research (V. Gennotte’s lab), (**e**) mRNA expression quantification through in situ hybridization assays in human breast cancer research (C.Josse’s lab), (**f**) Cell types in fine-needle aspiration cytology in human thyroid (I. Salmon’s lab), (**g**) Landmarks in *Danio rerio* embryo development (M. Muller’s lab), (**h**) Phenotypes in *Danio rerio* toxicology research (M.Muller’s lab), (**i**) Region delineation and cell counting in immunohistochemistry images in renal ischemia/reperfusion research (F.Jouret’s lab), (**j**) Cell scoring in immunohistochemistry images in melanoma microenvironment research (P.Quatresooz’s lab), (**k**) Nucleus counting in H&E images in human breast cancer research (E. De Pauw’s lab)

While our first developments were primarily motivated by the analysis of brightfield cytology and histology images (digital slides) in lung cancer research (Marée *et al.*, 2013b), we have significantly increased our software’s versatility and improved its extensibility. Cytomine has now been used on various bio(medical) imaging datasets that involved various types of images and experts in different collaborative operating modes to perform various quantification tasks. In particular, we briefly present here several use cases to help readers to determine how they can use our software to address their own research questions (see illustrative examples in Fig. 3 and Supplementary Note 5 for a user guide). These applications were regrouped into 4 categories corresponding to different image recognition tasks.

### 3.1 Tissue area quantification

In these use cases, scientists aims at quantifying the size (area) of tissue regions (e.g. the ratio of tumor islets with respect to whole tissue sections). This type of task implies to delineate the whole tissue section as well as the specific regions of interest within the tissue, either manually or semi-automatically (see Supplementary note 5.2.4.1 for a step-by-step guide using automatic recognition algorithms on toy data).

Following these principles, Cytomine enabled semi-automatic tumor area assessment in hundreds of whole lung Hematoxylin–Eosin (H&E) stained digital slides in mice inflammation and cancer research (Marée *et al.*, 2014) (Fig. 3a). Experts (pneumologists and biomedical researchers) first used Cytomine-WebUI drawing tools to provide manual tumoral islets and non-tumour annotations. Cropped images of these annotations were retrieved using web services and fed into our supervised learning algorithms for semantic segmentation. The task was formulated as pixel classification problem using multiple outputs. User interfaces and communication mechanisms to launch algorithms from Cytomine-WebUI were implemented to allow experts to execute training and prediction algorithms in an autonomous way. As our algorithms were not perfectly recognizing tumors, tools were implemented in Cytomine-WebUI to allow scientists to proofread annotations that were generated automatically. Experts are therefore able to accept or reject annotations and edit their shapes using drawing tools which allow to edit vertices, scale, substract or merge polygons, or fill internal holes. These manual operations are automatically translated internally into spatial queries on polygons, and validated annotations are stored in Cytomine-Core. After expert validation, statistics can be exported in standard formats for further analysis.

A similar workflow was used in (Leroi *et al.*, 2015) for semi-automatic tumor delineation in tens of whole Hematoxylin–Diaminobenzidine (HDAB) stained immunohistochemical digital slides in mice lung cancer research (Fig. 3b). Manual annotations (tumor, stroma and necrosis) were provided by experts to build a binary semantic segmentation model whose predictions were then proofread. In this study, this step was followed by quantitative assessment of antibody staining in relevant tissue area.

Finally in (Suarez-Carmona *et al.*, 2015), Cytomine was used to enable independent assessment by two observers (using the blinded configuration mode) of recruitment of CD11b + GR1 + myeloid-derived suppressor cells using mouse ear sponge models from whole immunofluorescent stained frozen sections. Experts used manual freehand annotation tools and multidimensional image visualization interface to analyze and merge fluorescent images (Fig. 3c).

### 3.2 Scoring and object counting

In these use cases, scientists aim at scoring or counting ‘objects’. This type of task implies to define (manually or automatically) regions of interest and count different types of ‘objects’ (e.g. cells marked with a marker-specific antibody) within these regions.

Cytomine was used to enable independent assessment by two observers (using our blinded configuration mode) of tens of thousands of BRCA1 mRNA expression signals and nucleus counts by in situ hybridization assays in tens of formalin-fixed, paraffin-embedded tissues in human breast cancer research (Boukerroucha *et al.*, 2015) (Fig. 3e). This study involved pathologists (for manual tumor delineation) and biomedical researchers (for manual annotation of spots and nucleus using point annotations). It required the development of web services performing polygon intersection operations to count spots within specific regions of interest. In addition, scripts using these web services were implemented to export quantification statistics in standard formats for further statistical analysis. Similarly, experts in sexual maturation research performed manual classification and counting (using point annotations) of thousands of oocytes in whole H&E slides of Chondrostoma nasus (Fig. 3d).

In Weekers *et al.* (2015), semi-automatic counting of immuno-reactive cells in regions of interest (cortex, medulla, corticomedullary junction) of tens of kidney sections was performed (Fig. 3i). Experts (nephrologists, pathologists and biomedical researchers) first provided manual freehand annotations (regions of interest, positive and negative cells) to train semantic segmentation models. These were applied for positive cell detection whose statistics were exported for each region of interest.

Other applications include manual double-blind scoring within tissue subregions from immuno-histostained digital slides in melanoma cellular microenvironment research (Fig. 3j), and manual point annotations of hundreds of thousands of nuclei for microproteomics from small regions of interest in H&E formalin-fixed paraffin-embedded tissue samples in human breast cancer research (Longuespée *et al.*, 2015) (Fig. 3k).

### 3.3 Labeled ground truth creation and object classification

In this family of tasks, scientists aim at sorting ‘objects’ (e.g. to detect rare abnormal cells or phenotypes). This type of task implies to detect objects and then classify them according to predefined categories (see Supplementary Note 5.2.4.2 for a detailed guide on using automatic detection and recognition algorithms on cytology toy data, and Supplementary Note 5.2.7.2 that describes how to create independent ground truth data).

Following these principles, Cytomine enabled manual semantic annotation of eleven categories of *Danio rerio* larva defects (e.g. edema, dead, curved tail,…) in hundreds of brightfield microscopy images by consensus voting of three biologists (Fig. 3h). These annotations were then used as ground-truth to build a worfklow for automatic phenotype classification using tree-based supervised learning (Jeanray *et al.*, 2015).

In Marée *et al.* (2016), we analyzed tissue components in human renal biopsies (Masson-Trichrome stain). We proposed an automatic glomeruli detection workflow combining image processing operations using Icy (de Chaumont *et al.*, 2012) and variants of our supervised classification algorithms. Icy was registered in Cytomine-Core using our software parameter templating mechanisms and it was therefore able to import and export image and annotation data using our web services (see examples in Supplementary Note 5.3). To build a large ground truth dataset (almost thirty thousand tissue components), glomeruli candidates automatically detected by Icy were analyzed using our proofreading tools for object classification. These interfaces show galleries of classified objects and allow a user to readily validate or correct (by drag and drop) predictions of ontology terms.

Other large ground truth datasets were collected using manual annotation tools. Several thousands of cells were annotated to build a large ground truth dataset in human thyroid cytology for the (ongoing) development of rare cell detection algorithms (Fig. 3f), inspired by previous work on cervical cancer screening (Delga *et al.*, 2014). We also implemented novel user interfaces (Cytomine-IRIS, see Supplementary Note 5.2.7.2) to enable different users to independently assign ontology terms to objects of interest. In particular, it was used by several pathologists to annotate bone marrow cells in order to study inter-observer agreements and build a large concordant ground truth dataset for the design of cell classification algorithms.

### 3.4 Landmark detection and morphometric measurements

In this fourth family of quantification tasks, the goal is to detect specific landmarks (or interest points) in images to perform morphometric measurements (e.g. distances between skeletal points in developmental studies). This implies to scan images to identify localizations of specific points (see Suppl Note. 5.2.4.3 for a step-by-step guide using automatic recognition algorithms on toy data).

Cytomine was used to perform manual annotation of tens of thousands of landmarks (positioning and naming) in hundreds of microscopy images of *Danio rerio* embryo for morphometric measurements in hormonal and hypergravity bone development studies (Aceto *et al.*, 2015) (Fig. 3g). These annotations are currently used to design and evaluate a generic landmark detection algorithm, following previous work in cephalometry (Huang *et al.*, 2015). For this type of tasks, we implemented proofreading web interfaces to rapidly and precisely visualize the localization of detected interest points, and to manually move them if they are not well positioned.

## 4 Discussion

The proposed software and its algorithms have already been applied to a wide variety of image types to accelerate discovery and to enable collaborative analysis. These results encourage its exploitation in many domains. However, in practical applications, obtaining satisfactory recognition performance using automatic algorithms depends on many factors including image variations (e.g. due to image acquisition and sample preparation protocols), and the quality and quantity of annotations provided for training (see e.g. empirical evaluations in Supplementary Note 4.2). Although the combination of our algorithms and proof-editing tools enabled to derive relevant quantification results in various applications, it is important to note that further adaptation of algorithms or developing novel recognition algorithms might be needed for specific types of images or varying acquisition conditions. A key advantage of our platform is therefore its extensibility. Indeed, our architecture enables computer scientists to add their novel software, register them to the Cytomine-Core and launch them from Cytomine-WebUI or from the command line. Also, annotation objects created by each instance of a software are stored in the database and are available through web services. These can then be subsequently proofread, or retrieved through the API by other software for further analysis and creation of novel – more precise – annotation objects. This allows to create complex image analysis pipelines based on distributed software.

It has to be noted that although the software allows visualization of 5D image planes (x,y,z,c,t), current applications cited in Section 3 have involved independent analysis of 2D image planes only (e.g. fluorescent image planes in (Suarez-Carmona *et al.*, 2015) and tissue slices in (Marée et al., 2014)). Using our API based on web services, one is able to extend the software by designing analysis algorithms that integrate 5D information if needed, or to interoperate with existing software.

## 5 Conclusion

Cytomine is a versatile software for collaborative analysis of multi-gigapixel images as already demonstrated by its various applications. With our design choices, we also believe our platform will facilitate accessibility, curation and dissemination of imaging-related data. In the future, it might be extended and tailored to support: (i) the setup of large-scale, multi-centric image repositories or the emergence of an imaging ‘data bazaar’ (Poldrack, 2014) to enable new research questions or validate results on larger cohorts, (ii) the organization of image analysis challenges on unprecedented benchmarks to foster image machine learning research, (iii) the crowdsourcing of image annotation tasks to tackle intractable datasets, (iv) the dissemination of multi-gigapixel imaging data and associated quantification results to support scientific claims of research papers and (v) increase the reproducibility of scientific results by providing a platform where published results are available along the algorithms and the image data. We have also started to derive the software for teaching purposes (see Supplementary Note 4.1.2).

## Supplementary Material

Supplementary Data
