# Supplementary material for: Collaborative analysis of multi-gigapixel imaging data using Cytomine
Source: Bioinformatics. 2016 Jan 10;32(9):1395–401. doi: 10.1093/bioinformatics/btw013 (PMC4848407; doi:10.1093/bioinformatics/btw013)
Supplement: Supplementary Data [file supp_32_9_1395__index.html]

Collaborative analysis of multi-gigapixel imaging data using Cytomine — Collaborative analysis of multi-gigapixel imaging data using Cytomine — Supplementary Data 

# Collaborative analysis of multi-gigapixel imaging data using Cytomine

## Supplementary Data

files

- Supplementary Data - pdf file
