# Supplementary material for: Collaborative analysis of multi-gigapixel imaging data using Cytomine
Source: Bioinformatics. 2016 Jan 10;32(9):1395–401. doi: 10.1093/bioinformatics/btw013 (PMC4848407; doi:10.1093/bioinformatics/btw013)
Supplement: Supplementary Data [file supp_btw013_maree-bioinformatics-2015-supplementary-material-revision2.pdf]

Collaborative analysis of multi-gigapixel imaging data using Cytomine

Raphaël Marée[1,2], Loïc Rollus[1], Benjamin Stévens[1], Renaud Hoyoux[1], Rémy Vandaele[1], Gilles Louppe[1], Jean-Michel Begon[1], Philipp Kainz[3], Pierre Geurts[1], Louis Wehenkel[1]

[1]Systems and Modeling, Department of Electrical Engineering and Computer Science and GIGA-Research, University of Liège, Belgium.
[2]Currently also affiliated with Bioimage Analysis Unit, Institut Pasteur, Paris, France
[3]Institute of Biophysics, Medical University of Graz, Austria

Correspondence should be addressed to Raphael.Maree@ulg.ac.be

Supplementary Material

Supplementary Table of Contents

Supplementary note 1: Methods - Software architecture (pages 3-5)
Supplementary note 2: Methods - Core data model (pages 6-10)
Supplementary note 3: Methods – Analysis modules (pages 11-14)
Supplementary note 4: Results - Empirical evaluation (pages 15-21)
Supplementary Note 5: User guide (pages 23-62)
Supplementary References (page 65)

Supplementary note 1: Methods - Software architecture

1.1 Overview

Cytomine is designed to enable multidisciplinary and distributed collaboration (as illustrated in Article Figure S1). In this Section we describe briefly the internals of the four main modules of our software illustrated by Figure 1.1: Cytomine-Core, Cytomine-IMS, Cytomine-WebUI, and Cytomine-DataMining. Our software follows the representational state transfer (REST) architecture and it is based on abstract communication interfaces so that main components (and users) can be at different physical locations (they are referenced by uniform resource locator (URL)).

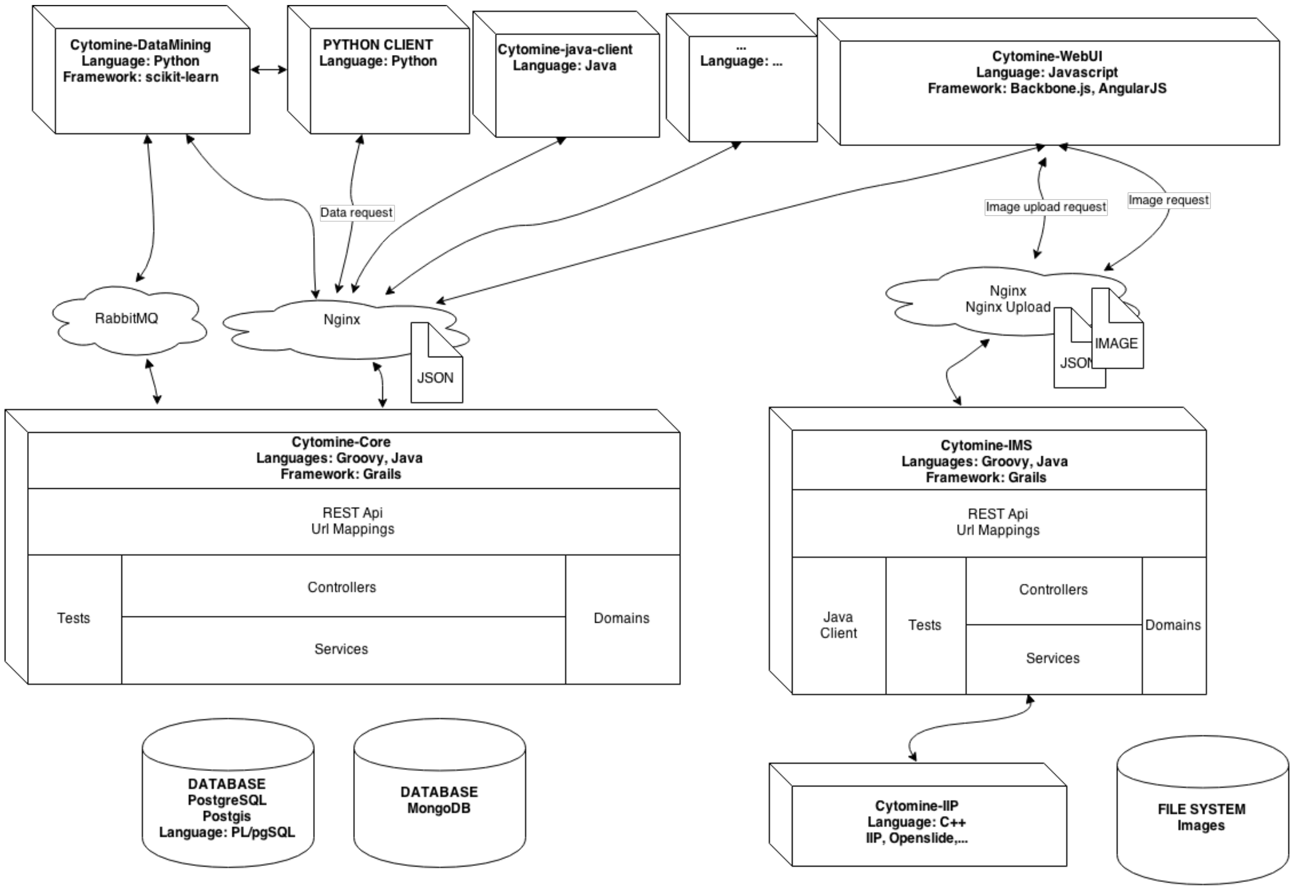

Figure 1.1: Cytomine global architecture with Cytomine-Core, Cytomine-IMS, Cytomine-WebUI, and Cytomine-DataMining

The Cytomine core server (**Cytomine-Core**) (https://github.com/cytomine/Cytomine-core) is a web application backend written in Groovy/Java with the Grails framework. It seamlessly integrates several libraries (see Section 2.2). It is based on model-view-controller (MVC) design patterns. While most of the data are actually stored in spatial SQL and NoSQL engines (See Supplementary note 2 for a description of the data model), the application provides RESTful web services to manage all these data (it is documented here http://demo.cytomine.be/restApiDoc/?doc_url=http://demo.cytomine.be/restApiDoc/api#) and relies mostly on the Hibernate framework for object-relational mapping. NoSQL engines were choosen to log effectively user activities in settings with thousands of users (see Supplementary Note 5.1 about our teaching server instance).

Image operations (image upload and extraction of image areas and annotation masks) are performed through the Cytomine Image Management System (**Cytomine-IMS**) (https://github.com/cytomine/Cytomine-IMS). Like Cytomine-Core, it is a MVC web backend application written in a collection of languages and frameworks. By combining lightweight image servers (namely IIPImage) and libraries (namely VIPS and OpenSlide), it currently supports standard 2D image formats (Pyramidal or non-pyramidal TIFF/BigTiff, jpeg, png, gif) and major 2D whole-slide scanner image formats (including Hamamatsu Ndpi, Aperio svs, 3DHistech mrxs, Leica SCN, Philips Tiff, Ventana Bif, Huron Tiff, Zeiss CZI once converted to Tiff). Pyramidal TIFF and whole-slide scanner image formats do not require to store many independant tiles on disk. They are designed to enable fast random access to small image areas from the whole image file (without loading the whole image into memory). Using domain sharding, we enable parallelization of image tile delivery by running multiple instances of Cytomine-IMS and by associating (in our data model) a single image to multiple image servers URLs. Cytomine-Core then redirects seamlessly and randomly each image tile HTTP request to one of these Cytomine-IMS, distributed, server. This trick overcomes the limitation of simultaneous connections to a single host in web browsers, resulting in a faster user experience.
Our system also supports many microscopy 5D image sequences (x,y,channel,slice,timeframe) formats through seamless conversion to 2D OME-TIFF image sequences during uploading phase. Extension to novel image formats or optimizations could be implemented either as extension of current image server instances (through extension of currently used libraries, namely VIPS, Openslide and BioFormats) or by developing connectors to other image servers e.g. for 3D images using [8] or molecular MALDI imaging using [9].

The Cytomine user interface (**Cytomine-WebUI**) (https://github.com/cytomine/Cytomine-core/tree/master/web-app) is an MVC web client application written in Javascript, HTML5 and CSS3 which runs on regular web browsers (Google Chrome, Mozilla Firefox, Apple Safari) without the need to install plugins. It uses HTTP digest access authentication and emits HTTP requests to Cytomine-Core and Cytomine-IMS to display data dynamically into web pages or to update data persistently into the database. Cytomine-IRIS (https://github.com/cytomine/Cytomine-IRIS) is a derived web client for inter-observer studies and it follows the same design principles. It illustrates the possibility to extend our software for specific purposes. To ease the development of other extensions, we provide a simple web application template to start with: https://github.com/cytomine/Cytomine-sample-webapp).

The analysis modules (**Cytomine-DataMining**) are relying on Java and Python reusable clients (https://github.com/cytomine/Cytomine-java-client and https://github.com/cytomine/Cytomine-python-client) to extract/import data from Cytomine-Core and Cytomine-IMS. These clients provide an easy way to get/update Cytomine-Core/IMS data using an external application/script through abstract HTTP calls, JSON messages, and HTTP status codes. Simple examples are provided to manipulate data (Java: https://github.com/cytomine/Cytomine-java-client/tree/master/src/main/java/be/cytomine/client/sample and Python: https://github.com/cytomine/Cytomine-python-client/tree/master/client/examples). Developing clients in other languages is possible provided these implement basic HTTP operations.
In terms of currently integrated algorithms, we first integrate an unsupervised image recognition library (https://github.com/loic911/CBIRetrieval) that is a multi-threaded implementation in Java of our unsupervised content-based image retrieval algorithm [10]. The retrieval server is directly called by Cytomine-WebUI once a user selects or draws an annotation.
The supervised image recognition modules are multi-threaded implementations for image classification, semantic segmentation, and landmark (interest point) detection [11]. These modules are implemented in Python and rely on its stack of numerical and machine learning libraries

(numpy, scikit-learn). Algorithms and examples of how to run them are provided (https://github.com/cytomine/Cytomine-python-datamining/tree/master/cytomine-applications). As other modules, they communicate with Cytomine-Core and Cytomine-IMS through HTTP requests. These modules can be launched through command-lines on remote servers, or executed manually from the Cytomine-WebUI. We use a message broker (RabbitMQ) implementing AMQP (Advanced Message Queuing Protocol) to invoke Cytomine-DataMining modules from Cytomine-WebUI and Cytomine-Core.

Although Cytomine is a complex software with tens of thousands of code lines and integrating many libraries, we use software containerization techniques (with Docker) to fully automate its deployment on regular desktops or servers. Scripts for installation and update of a Cytomine instance are provided: https://github.com/cytomine/Cytomine-bootstrap
Full installation procedure is described here: http://doc.cytomine.be/x/goCj

1.2 List of libraries integrated into Cytomine

We integrated several open-source libraries to build the Cytomine software. Figure 1.2 summarizes major integrated libraries. A full, continuously updated list is available on:
http://doc.cytomine.be/x/AYGS

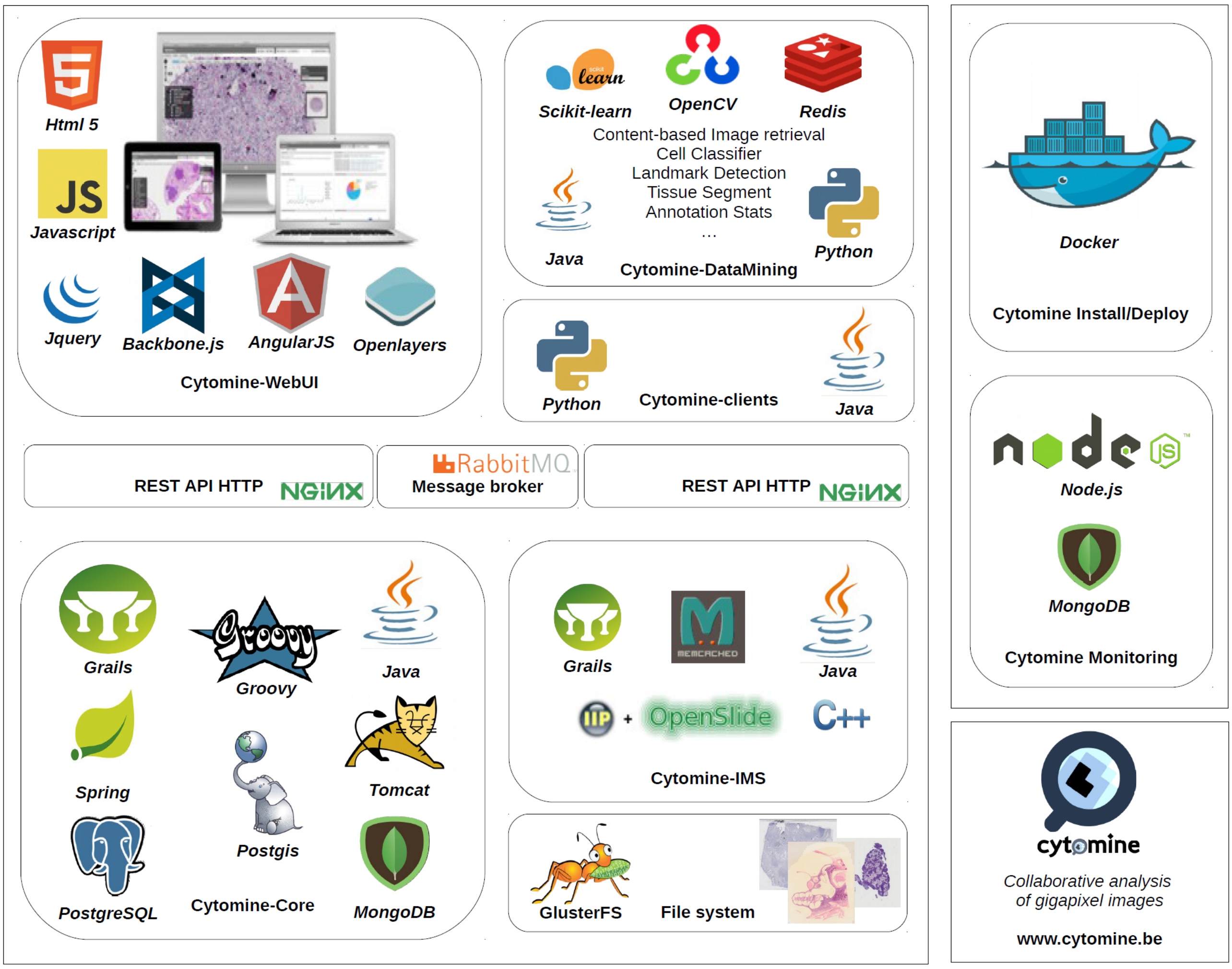

Figure 1.2: Main libraries integrated into Cytomine.

Supplementary note 2: Methods - Core data model

2.1 Data model overview

In this section we describe briefly the main relational data entities of the data model of Cytomine-Core using simplified entity relationship diagrams. More details are available on http://doc.cytomine.be/x/ZYCS

An overview of main data entities is given by Figure 2.1. The central Cytomine data object is the Project that contains User(s), an Ontology, Image(s), Annotation(s) within images, Software, and Properties that can attached to Projects, Images, and Annotations. Each resource is identified by a unique identifier (encoded in 64 bits). In addition, user activity data (such as user position and last connection) are stored in a non-relational database. Properties/Description/AttachedFile/Metadata can be associated to Projects, Images, and Annotations. Properties are key-value pairs, descriptions are rich text descriptions (in HTML), AttachedFile are any supplementary file (e.g. PDF), and Metadata.

AbstractImages (Figure 2.2) are 2D images uploaded to a storage server and can be accessed through an ImageServer. ImageInstances are abstract images associated to a project. ImageInstances can be linked into ImageSequences (each image in an ImageSequence is then identified as a 3-tuple for additional dimensions: c for channels, z for slice, t for time) and associated with a project as ImageGroups (Figure 2.3).

Both human users and software need to authenticate and have access rights to given project(s) through authentication (Figure 2.4). A user is also associated with one or multiple user groups (superadmin, admin, user, guest) which determines actions he/she is allowed to perform: e.g. a guest user can visualize project contents but not edit existing objects and a regular user can view and edit all data in projects for which he has access rights. If a regular user is member of the admin role group, she/he can switch to this role and perform any operation on data from all projects.

An ontology is an editable list of terms with relations (Figure 2.5). A single project contains only a single ontology, but a given ontology can be associated with multiple projects. One or multiple terms of an ontology can be associated to any annotation in the project.

Annotations are geometrical objects (e.g. circle, rectangle, polygon, freehand, point) that can be drawn manually in Cytomine-WebUI by regular human users (UserAnnotations) or created by job instances (UserJobAnnotations). Each annotation is associated to one ImageInstance (i.e. it corresponds to a region of interest in an image). Additionally, a user can review Annotations created in an image and create ReviewedAnnotations that can be a mix of validated UserAnnotations or UserJobAnnotations (Figure 2.6).

Software is an object that can be associated with project(s) to run any processing commands. They can be described by a list of software parameters (name and value type). A job is a software instance with specific parameters values. A jobTemplate is a set of predefined parameter values for a given software. Jobs inherit access rights from the human user who invoked them ie. it is allowed to add/edit/delete the same resources (Figure 2.7).

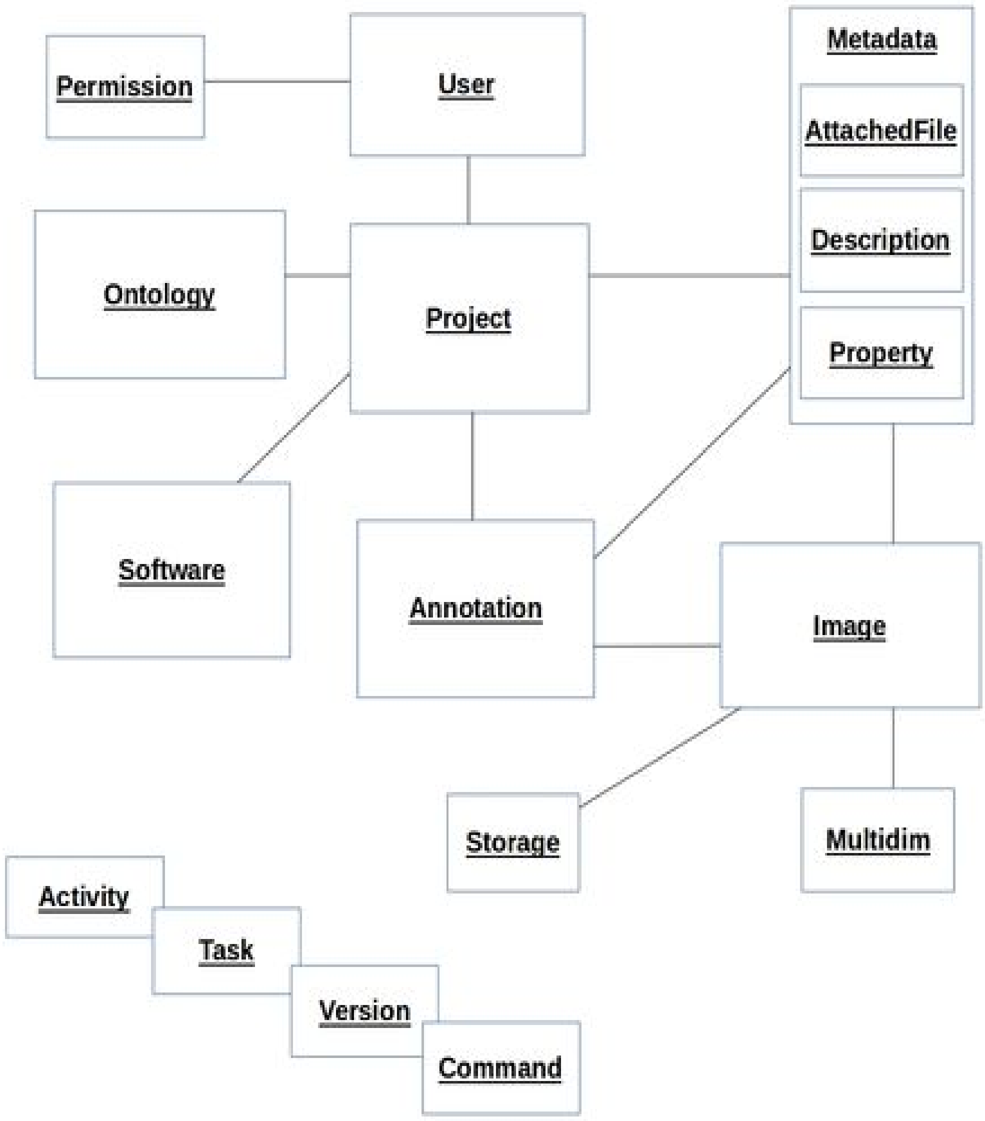

Figure 2.1: General overview of the Cytomine data model.

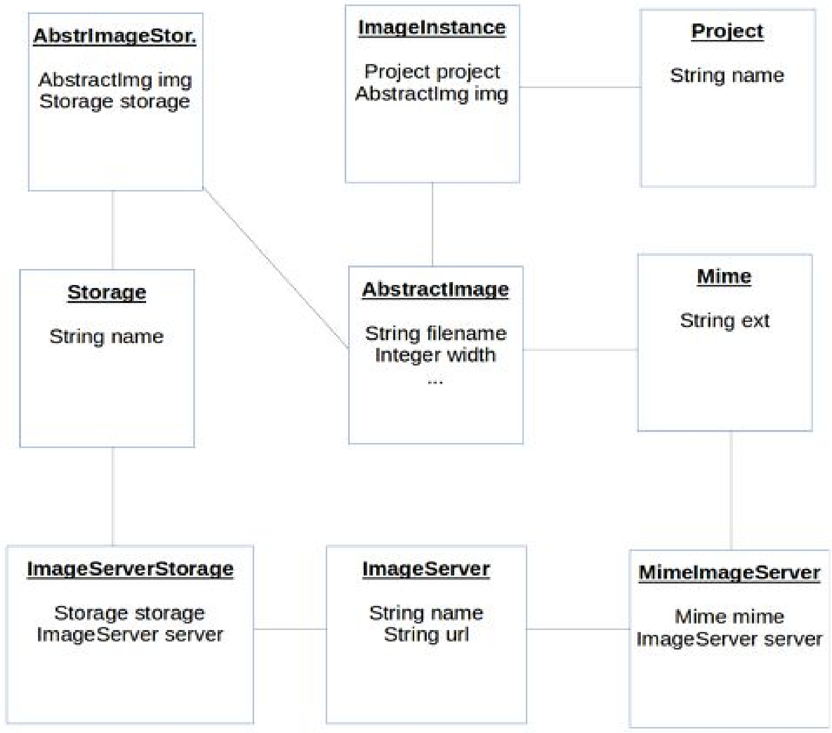

Figure 2.2: Data Model: Image.

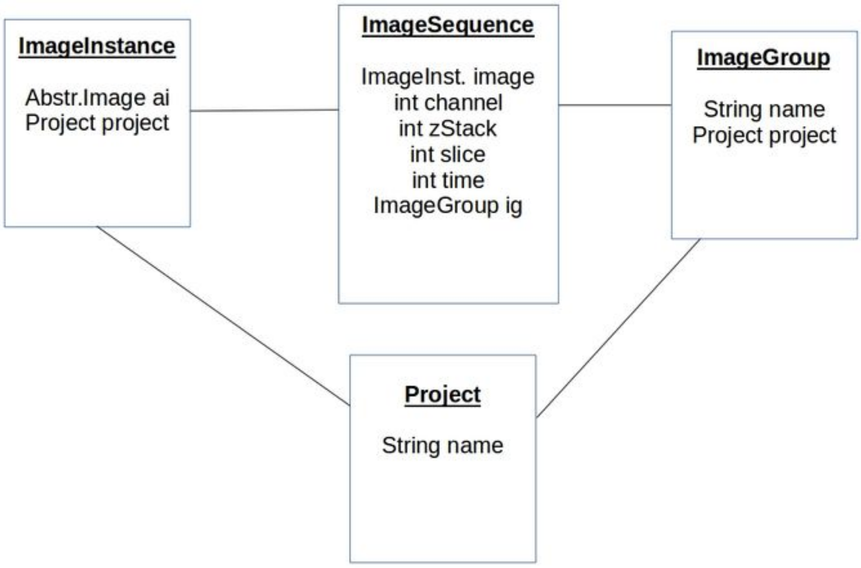

Figure 2.3: Data Model: Sequence of images (for multidimensional images).

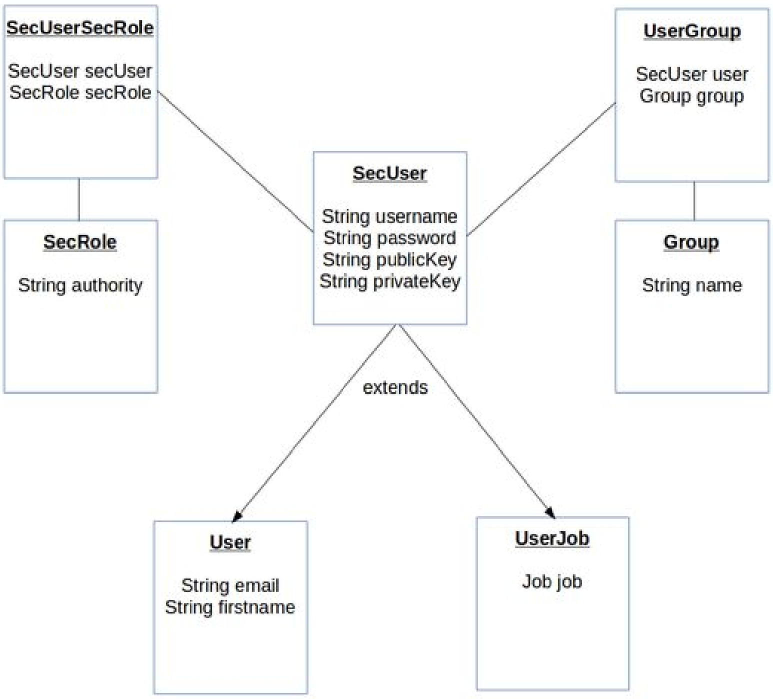

Figure 2.4: Data Model: Security for users and softwares.

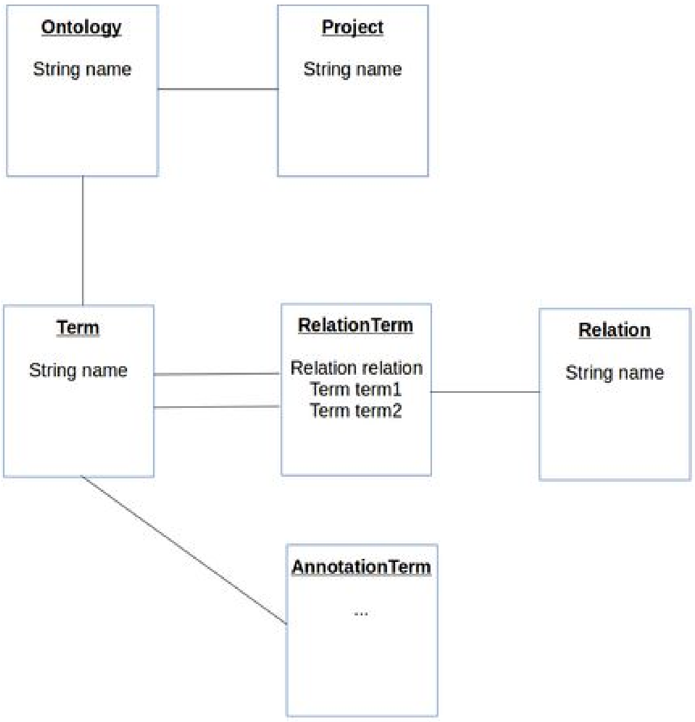

Figure 2.5: Data Model: Ontology for semantic annotation.

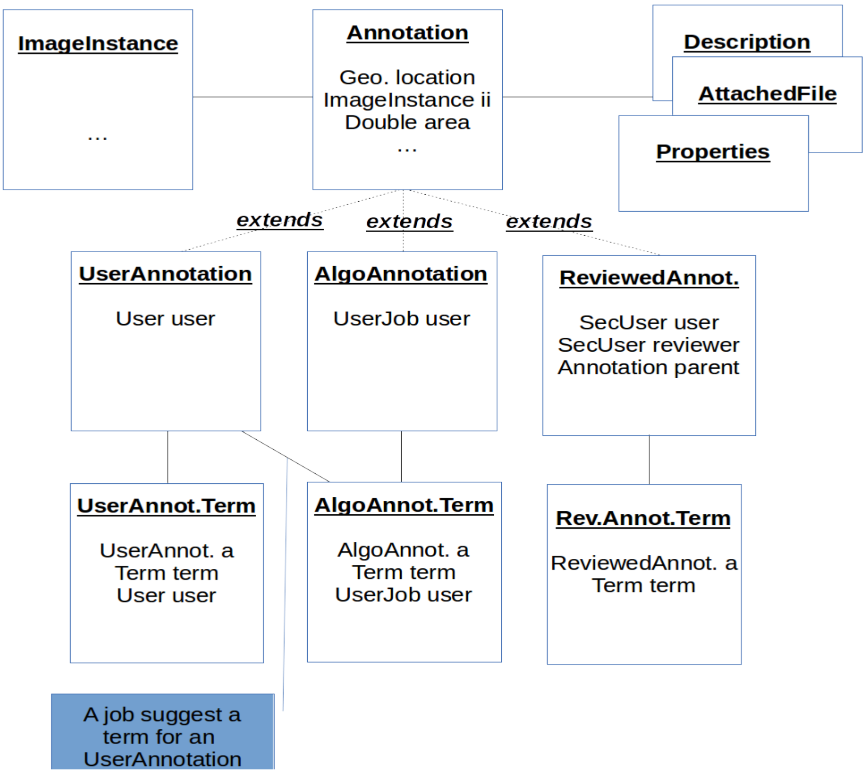

Figure 2.6: Data Model: Annotations

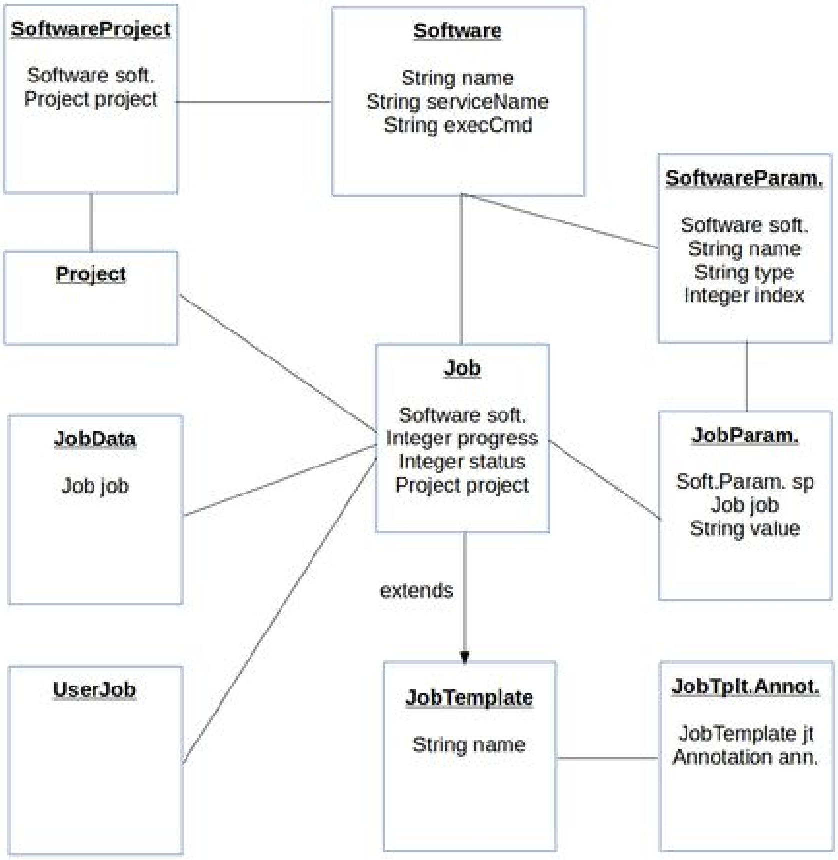

Figure 2.7: Data Model: Software, jobs and parameters

Supplementary note 3: Methods – Analysis modules

Cytomine analysis modules (Cytomine-DataMining) are novel implementations of previously published algorithms and novel variants. These modules are specializations of an unified framework [11] involving the extraction of random subwindows within images and the induction of ensembles of extremely randomized trees. They can be used to solve general problems in computer vision, ranging from image classification and segmentation to content-based image retrieval and landmark detection.

Here we describe their basic principles and their novel multi-threaded implementation to interact with other Cytomine components. These analysis modules are able to download images and annotation data from Cytomine-Core and Cytomine-IMS, process them locally on a server hosting the algorithm (e.g. to learn segmentation/object classification models then segment or classify objects in tiles of new images), and upload results (e.g. annotation geometries in WKT format and associated term identifiers (e.g. cell category) or properties (e.g. cell counts) to Cytomine-Core. Annotation objects created by each instance of a software are stored in the data model as UserJobAnnotations. These can be subsequently retrieved through the RESTful API by other software for further analysis. This allows to create complex image analysis workflows based on distributed software. Annotations objects that are created by these algorithms can also be proofread by human experts using Review modules on Cytomine-WebUI. These are then stored in the data model as ReviewedAnnotations (with a link to their parent UserJobAnnotation) and these novel annotations can be used either to retrain algorithms or to produce final quantification results. These analysis modules are evaluated in Supplementary Note 4, and examples of their usage on "toy" data are given in the user guide (Supplementary Note 5).

3.1 Detect Sample

This module applies an adaptive thresholding algorithm to whole image thumbnails from a project (downloaded from Cytomine-Core and Cytomine-IMS) and uploads detected geometries to Cytomine-Core (in a individual UserJob layer for each image). It can be used to detect tissue sample regions in order e.g. to quantify tissue sample area or before applying other algorithms (e.g. segmentation) to avoid applying time-consuming algorithms to image background regions.

Source:

https://github.com/cytomine/Cytomine-python-datamining/tree/master/cytomine-applications/detect_sample/

3.2 Object Finder

This module applies an adaptive thresholding algorithm and a connected component algorithm to individual tiles in a whole gigapixel image and uploads detected contours (geometries) to Cytomine-Core (in a UserJob layer). Its typical use is object detection (e.g. cells) before object classification step. This procedure can be applied on tiles extracted at any resolution level, as the conversion to maximum resolution coordinates is performed automatically by a local function. Future developments might easily adapt the thresholding algorithm for specific purposes.

Source:

https://github.com/cytomine/Cytomine-python-datamining/tree/master/cytomine-applications/object_finder

3.3 Image/Object classification

The aim of supervised image classification is to automatically build computerized models able to predict accurately the class (among predefined ones) of new images (objects), once trained from a set of labelled images (objects). A typical example is individual cell classification in cell populations.

This module is decomposed into three components: a validation phase (validation), a training phase (model builder) and a prediction phase (prediction). The validation phase downloads from Cytomine-Core, for a given list of projects and additional filters (terms, users,...), cropped image regions for each annotation (minimal bounding box fully containing the annotation geometry). Using a cross-validation protocol, it learns classification models (see algorithm below) and evaluates their classification accuracy for a given set of parameters. The predictions are uploaded to Cytomine-Core and the resulting confusion matrix can be visualized in the Cytomine-WebUI (confusion matrices are interactive, with the possibility to visualize annotation misclassifications directly in original gigapixel images). The training phase similarly downloads annotations, builds a classification model on the whole set of annotations for the given set of parameters and saves the classification model locally. The prediction phase applies such a model on existing annotations downloaded from Cytomine-Core within a gigapixel image (e.g. annotations detected by the object finder module), and uploads predicted annotation terms to Cytomine-Core (in a userjob layer).

The algorithm involves the extraction of random subwindows described by raw pixel values and the use of ensemble of extremely randomized trees by different means. More detailed algorithm description and illustration of its main steps are given in the technical report [12]: http://hdl.handle.net/2268/175525.

Source:

1. Validation: https://github.com/cytomine/Cytomine-python-datamining/tree/master/cytomine-applications/classification_validation
2. Learning: https://github.com/cytomine/Cytomine-python-datamining/tree/master/cytomine-applications/classification_model_builder
3. Prediction: https://github.com/cytomine/Cytomine-python-datamining/tree/master/cytomine-applications/classification_prediction

3.4 Semantic segmentation

This module aims at learning and applying pixel classifiers for semantic segmentation of images (e.g. tumor contouring). It is based on a previously published algorithm [19]. It is decomposed into a training phase (model builder) and a prediction phase (prediction).

For a given list of projects (cytomine_annotation_projects), the training phase downloads from Cytomine-Core cropped image regions for each annotation (minimal bounding box fully containing the annotation geometry) and its corresponding binary mask (in PNG format with alpha channel). Positive examples are selected using the cytomine_predict_terms variable, negative examples are other annotations that are not included into cytomine_predict_terms neither cytomine_excluded_terms. Then, the method builds a segmentation (pixel classifier) model using random, fixed-size (pyxit_target_width and pyxit_target_height) subwindow extraction and extremely randomized trees with multiple outputs [19]. The tree model is built using the same parameters than for classification. It is saved locally on the running server filesystem. The

prediction phase applies such a binary segmentation model on individual tiles of a gigapixel image. A connected components labeling algorithm is then applied on the running server to extract contours from each tile pixel classifier mask. These contours are then translated into valid geometric shapes for spatial database by using hit-or-miss transforms. Point coordinates of valid geometries found in each tile are then converted to real coordinates (in the whole slide image) and communicated to Cytomine-Core that internally translates the HTTP requests into spatial insertion queries (these annotations are added in the UserJob layer). Finally, once all tiles are processed, these contours are eventually merged by spatial union queries over tiles to take into account the fact that a single region of interest (e.g. a tumor islet) may actually overlap several tiles. Both training and prediction phases can be applied at any resolution level (cytomine_zoom_level) of gigapixel images with seamless conversion of coordinates. A typical application is tumor/inflammation detection in tissues in histology slides (see Supplementary Note 4.2.2 and 5.2.4.1).

Source:

1. Learning: https://github.com/cytomine/Cytomine-python-datamining/tree/master/cytomine-applications/segmentation_model_builder
2. Prediction: https://github.com/cytomine/Cytomine-python-datamining/tree/master/cytomine-applications/segmentation_prediction

3.5 Landmark detection

The goal of this module is to build and apply interest point (landmark) detection models. Typical applications include morphometric measurements in developmental studies. This module implements novel variants of an algorithm previously presented in [20]. This package is decomposed into a training phase (model builder) and a prediction phase (prediction). For the training phase, it downloads from a given project images and landmarks (x,y positions and landmark class) by communicating with Cytomine-Core. It then builds landmark detection models (one model per landmark) using multiresolution pixel-based features and extremely randomized tree classifiers. These models are saved in files locally. For the prediction phase, it downloads novel images and applies all landmark prediction models to each image. Landmark coordinates are uploaded to Cytomine-Core as Point annotations with predicted terms (landmark class).

More precisely, a binary classification scheme is used in the implementation. In this approach, each pixel in an image can either be a landmark or not. It is a landmark pixel if it is located at an euclidean distance <R to the real landmark position. Each pixel is described by square subwindows of size W taken at D different resolutions centered at the location of the pixel. If D resolutions are taken, the resolutions of the image divided by 2^0 to 2^D will be used. During training, all the landmark pixels are extracted from each image of our training dataset. If N is the number of landmark pixels extracted for one image, P*N non-landmark pixels are also extracted from each image, at a distance of at most RMAX to the landmark position. After this first extraction, the process is repeated Nr times by applying Nr random rotations to each image of the training dataset. These rotations will be chosen in the interval [-a,a]. A model of T extremely randomized trees is built. During prediction, we extract Npred pixels located at random positions chosen from a multivariate normal law based on the position of the landmark in the training dataset. The position of each landmark is then computed as the median of the locations of the pixels predicted as landmarks with the highest probability by the corresponding model.

All these parameters (R, W, D, P, RMAX, Nr, a, T, Npred) can be chosen individually for model building, or tuned through K-fold cross validation.

Source:

1. Learning & validation: https://github.com/cytomine/Cytomine-python-datamining/tree/master/cytomine-applications/landmark_model_builder
2. Prediction: https://github.com/cytomine/Cytomine-python-datamining/tree/master/cytomine-applications/landmark_prediction

3.6 Image retrieval

The goal of an image retrieval algorithm is to retrieve from a large database of images (the reference set), visually similar images to a query image.
We integrated a novel implemention in Java of a previously published algorithm [10] based on random subwindows and vectors of random tests. Description of this novel library is provided in [Paper in preparation].

Source:
1. CBIRetrieval: a Java library package that can be run as a simple application (command line) or can be imported in a JVM application (jar). https://github.com/loic911/CBIRetrieval
2. CBIRestAPI: an HTTP REST API server for CBIRetrieval lib. https://github.com/loic911/CBIRestAPI

Supplementary note 4: Results - Empirical evaluation

This supplementary note provides quantitative information about current version of Cytomine. It summarizes our current usage and empirical evaluations of basic operations and its recognition algorithms.

4.1 Software requirements, usage statistics, performances, and code quality

4.1.1 Software requirements

Minimal hardware requirements to host a Cytomine server are described here:
http://doc.cytomine.be/display/PubOp/%5BDOC%5D+Cytomine+Requirements
Medium-sized servers are able to host Cytomine components. Smaller instances have also been tested using our installation procedure on regular desktop computers e.g. for developers testing their algorithms on small datasets of gigapixel images.

4.1.2 Usage statistics in research and education

As an example, we have two running instances at the University of Liège: a research instance and a teaching prototype instance.

For our research instance, we use:

- Cytomine-Core: web server using 8GB memory and 4 * 2.3 Ghz processors and database server using 4 GB memory and 4 * 2.3Ghz processors ;
- Cytomine-IMS using 36 GB memory and 12 * 2.1Ghz processors).

As of 1st June 2015, it hosts (after four years of usage):

- 300 projects,
- 20200 images,
- 190 users,
- 621000 user manual annotations and 285000 annotation terms,
- 5582200 algorithm annotations (created by 10000 jobs) among which more than 50000 were proofread by experts.
- Images use 6.5TB of disk space.
- The raw database dump file is 100 GB.
- The largest 2D image uploaded by users is 106259 x 306939 pixels (32 Gigapixels).

In addition to use cases (see Main paper Section "Applications"), the use of manual annotation tools also results in unprecedented datasets that might be exploited in future studies to design automated algorithms. It includes more than 45000 landmarks in Zebrafish development images, more than 370000 nucleus counts in breast H&E images, more than 6000 cell classifications in thyroïd cytology images, more than 88000 nucleus and RNAScope/PLA spots in breast IHC images,....

For our teaching prototype instance, we use:

- Cytomine-Core: web server using 16 GB memory and 8 * 2.8Ghz processors and database server using 8 GB memory and 4 * 2.7Ghz processors,
- Cytomine-IMS using 8 GB memory and 4 * 2.7Ghz processors) hosts (after 1.5 years of usage):

As of 1st June 2015, it hosts (after one year of usage):

- 55 projects,

- 2050 images,
- 4015 (students and teachers) users,
- 189000 user manual annotations.
- Images use 1.5TB of disk space
- The raw database dump file is 3 GB.
- The largest 2D image uploaded by users is 196608 x 325632 pixels (64 Gigapixels).

In this setting, projects correspond to practical courses or homeworks. Annotations geometries and descriptions are used to reference important aspects of tissues. In particular, each student is following predefined observation paths to study normal and pathological tissues, or they have to annotate images or to answer some questions through a learning managment system communicating with Cytomine through the RESTful API.

4.1.3 Performances

Here we provide average timing for basic operations through a Wi-fi remote connection and that are executed on our research instance:
- Get the listing of all 300 project descriptions: 994 ms
- Get the listing of the first 100 image descriptions of a project: 1.44s
- Get the listing of 1005 annotations (size: 715KB) from a given user for a given image: 805ms
- Get the listing of annotation geometries of an object finder layer (49336 objects) and apply a K-Means algorithm for lightweight visualization within a gigapixel image (111104 x 91648 pixels), (see Figure of gigapixel pollen image in Supplementary Note 5.2.5): 12s

4.1.4 Code quality

In terms of code, at time of submission, Cytomine is composed of 68K lines of code decomposed into its four main modules (Cytomine-Core +/- 30000 lines (Groovy/Java); Cytomine-WebUI +/- 23000 lines (Javascript/Html); Cytomine-IMS +/- 3000 lines (Groovy/Java); Cytomine-Analysis +/- 10500 lines (Java/Python)). These statistics do not take into account integrated libraries (Supplementary Section 1.2). During our developments, commits to the source repository trigger a new build in our continous integration server to assess code quality. 1150 unit and functional tests are executed. These tests cover 80% of the source code lines for Cytomine-Core.

4.2 Image recognition algorithms (Analysis modules)

In this section we describe or cite empirical studies of our algorithms recognition performances using public benchmarks or datasets manually collected by experts through Cytomine-WebUI. In practical applications, obtaining satisfactory recognition performances depends on many factors including image variations (due to image acquisition and sample preparation protocols), and the quality and quantity of annotations provided for training.While we do not claim our algorithms will provide accurate results on any problem, we illustrate here their potentials on a wide variety of image types. In practical applications, we generally used them in combination with Cytomine-WebUI proofreading tools to derive accurate quantifications from imaging data.

4.2.1 Image/Object classification

In life science research, efficient classifiers have a huge potential [21-25] as they could ease the labor-intensive phenotype classification of biological samples (e.g. cells, tissues, organs, microbes, diatoms, plants, animal models,...) thus facilitate obtaining more quantitative information in large-scale imaging datasets. Recent variants of our image classification algorithms have been evaluated on a large variety of datasets from many application domains

(more than 3800 image classes and over 1.5 million images). Description and references of these datasets, evaluation protocols, and empirical results are described in the following technical report [12]: http://hdl.handle.net/2268/175525.

4.2.2 Image semantic segmentation

The aim of image semantic segmentation is to classify pixels of an image and group pixels of the same class. Our experiments are based on a practical applications involving H&E histology images of mice lungs (provided by Didier Cataldo's Laboratory of Tumor & Development Biology, GIGA-Cancer, University of Liège) where the goal is to segment tumor islets to count them and compute their area to study the effect of molecules or biological processes on lung tumor onset and progression. Results for one experimental study was published in [26]. Here we report results using the same parameter values on 19 additional experimental studies (843 whole-slide images). As in [26], we compared a posteriori validated annotations (Review layer) and annotations produced by our algorithm (UserJob layer). Results (recognition performances and required user corrections) are variable due to tissue variations (e.g. these studies include various tumor subtypes, inflammatory regions that exhibit similarities with tumoral regions, color staining variations,...). We measured a posteriori that on average 89% of validated tumoral area was well detected by the algorithm (true positives). However we also observed a global trend of the algorithm to detect many regions that are not tumors with a false positive rate of 3%. Although this rate is low, in practice the algorithm detected on average 279 tumor islets per slide among which only 53 are validated by experts per slide (43 are left as such and 10 are manually edited). The retraining capability of our software was not used for these experiments. Retraining models with additional negative examples and post-processing of predictions (e.g. based on size as the algorithm tends to detect many small regions that are not validated by experts) might help to reach better recognition performances. Still, in practice, our algorithms were combined with our proofreading tools and our collaborators were able to produce quantifications for about 850 whole-slide images, which yielded the validation of a total of about 45000 tumoral ROIs that could be used in future studies to refine recognition models. The Figure 4.4 shows a thumbnail of a well-segmented slide (left: original, middle: algorithm predictions, right: final validated) where proofreading only consists in rejecting a few false positive regions (red arrows).

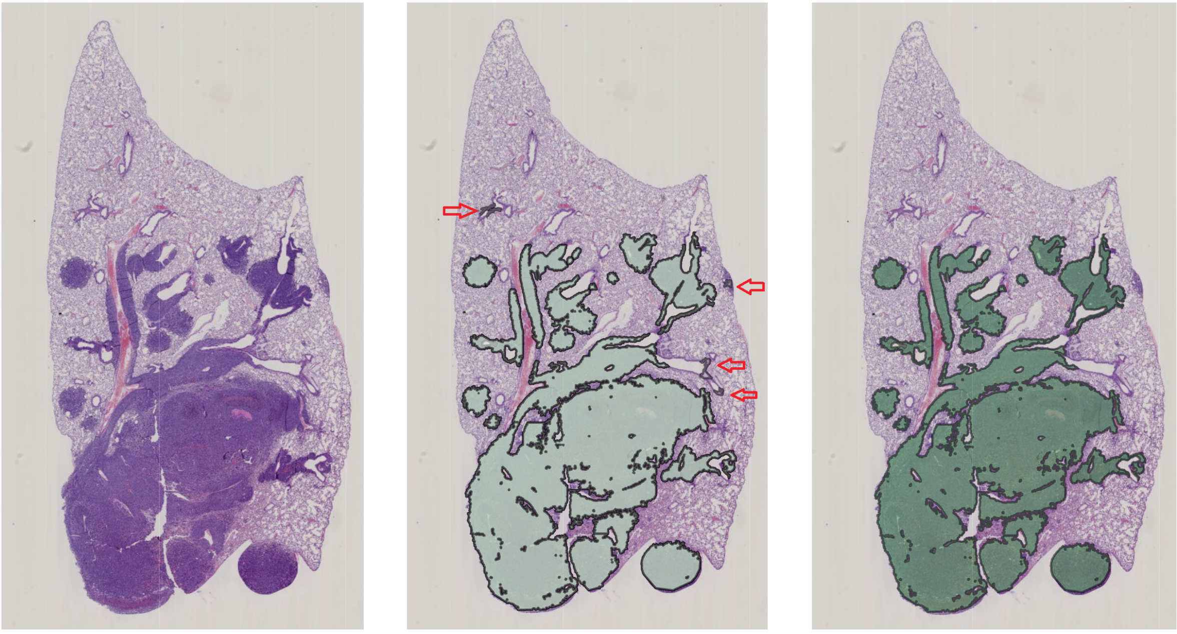

Figure 4.4: An H&E whole-slide mice lung (left), automatic tumor contouring (middle), final proofread tumor contouring (right).

4.2.3 Landmark detection

Our landmark detection algorithm was evaluated empirically on three different datasets: a dataset of 100 cephalometric images with 19 landmarks [27], a dataset of 113 Zebrafish images with 30 landmarks (from Marc Muller's lab, GIGA-Development, Stem Cells and Regenerative Medicine, Organogenesis and Regeneration, University of Liège, Liège, Belgium), and a dataset of 138 drosophilae wing images with 15 landmarks (from Frédérique Peronnet's research unit "Contrôle épigénétique de l'homéostasie et de la plasticité du développement", Laboratoire de Biologie du Développement - Institut de Biologie Paris-Seine, UMR7622 - CNRS - UPMC).

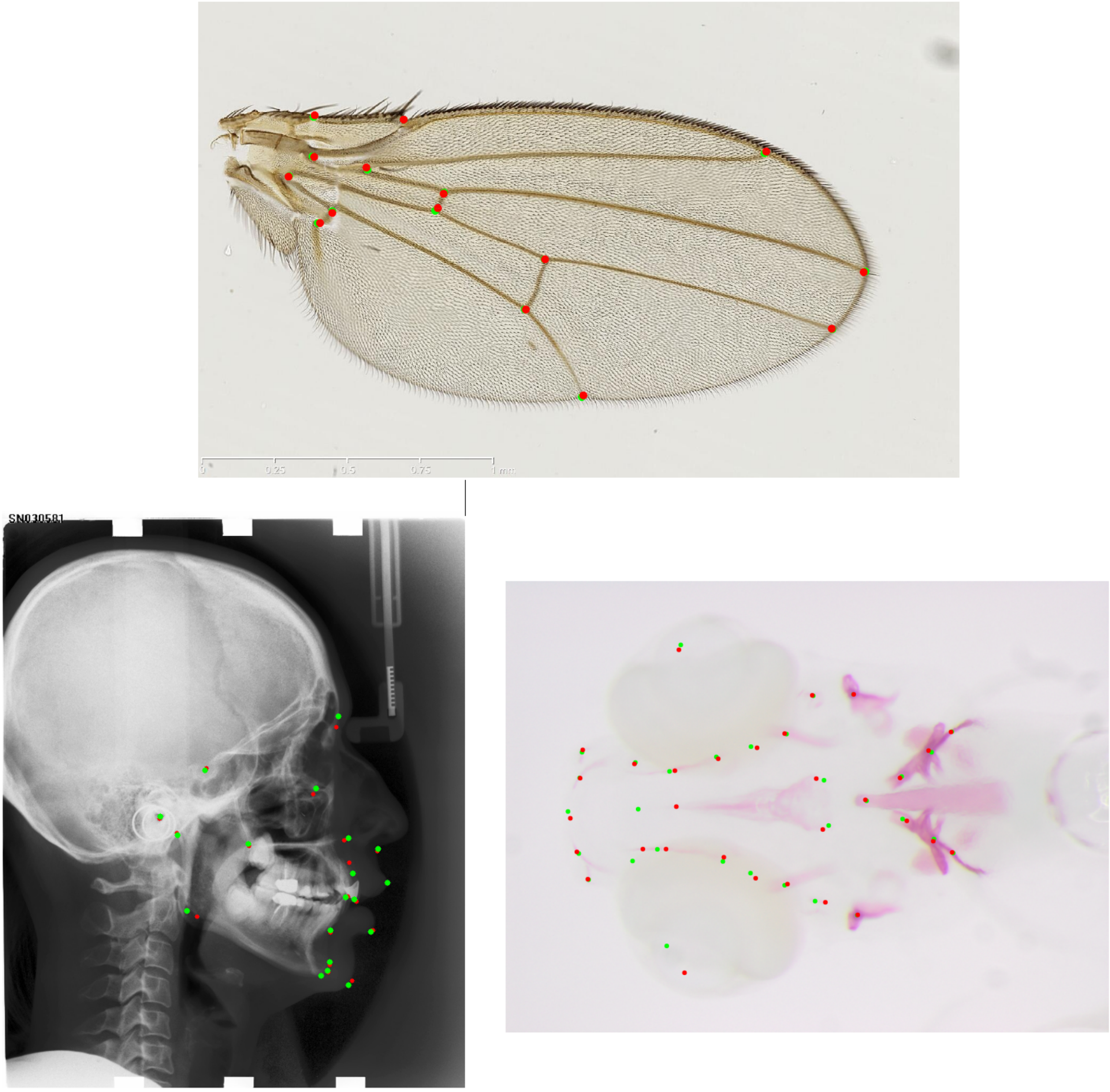

Figure 4.5: Landmark detection on 3 image types. Drosophilae wings (top), cephalometric X-ray (bottom left), Zebrafish development (bottom right)

Results of a preliminary version of our algorithm were presented in [27] for the cephalometric dataset (our algorithm ranked second in the ISBI 2014 Cephalometric challenge), and in [28] for the Zebrafish dataset. We draw here only general observations as a systematic analysis of the influence of the various parameters used in our method is ongoing and will be the subject of a future study. Current cross-validation results show a large diversity in accuracy between the different datasets. We have a mean error (distance between predicted landmark points and manual annotations) around

30 pixels for the landmark detection on the Zebrafish dataset (images of size 2576x1932), 20 pixels for the cephalometric dataset (images of size 2400x1935), and 5 pixels for the drosophilae dataset (images of size 1440x900). We noticed these differences are explained by different factors: the pixel accuracy of the manual annotations of the landmarks used to train the models (directly linked to the resolution of the image), and the diversity of the landmark shapes in the dataset that is related to the variability of images and the types of landmarks to detect. For example, on our Zebrafish dataset, some landmarks can be detected with an accuracy below 10 pixels, while others can only be detected with an accuracy below 50 pixels. Figure 4.5 provides examples of automatic detections (in red) on the three datasets with respect to manual ground-truth (in green). In practical applications, we recommend to combine automated predictions with Cytomine-WebUI manual proofreading tools where each landmark is colored according to its ontology term configuration (see Supplementary Note 5.*2.4.3)*.

4.2.4 Image retrieval

Our algorithm was previously evaluated in [10] on two medium-size public datasets (a radiology dataset and a dataset of sport scenes) and on a first small set of lung cancer histology and cytology images in [29].

Here we performed a wider empirical evaluation on various annotations created through years by research users of the Cytomine-WebUI within our institute. To perform this evaluation, we worked on a subset of annotations from our Cytomine research instance: We selected polygon annotations created by human experts and we kept only those from ontology terms for which at least 10 annotations were created. In total, our reference dataset contains 96000 annotations associated to 294 ontology terms. We evaluated recognition rates using different parameter values and aggregation schemes and here summarize our results when using N=1000 random subwindows (full intervals) resized to 16x16 in HSV, T=10, and 30 tests per vector. All queries are performed by searching for similar annotations in the reference set of annotations which belong to the same ontology as the query. We first evaluated how often a given query image is retrieved as the most similar image (identity search) when considering all images of the ontology as the reference database (query-top-1 = 84%). We then remove the query image from the reference set and evaluated how often an annotation with the same term than the query is retrieved as first similar image (top-1 term = 82%). We then evaluated if considering the top-10 retrieved images and aggregating (by weighting proportionally with their similarity rate) their terms provide better term suggestion (top-10 avg term = 79%, top-10 avg-weighted = 80%).

In terms of computing times, our implementation can be run using regular data structures or optimized hashtables. We recorded indexing and retrieval times on a laptop computer (8 cores, 32Gb Ram, SSD disks). Our regular implementation requires on average 425ms to index an annotation and 201ms to retrieve similar images (using N=500 and T=5) which provides top-1 term accuracy = 79%. The optimized implementation with same parameters requires 178ms on average for indexing but 632ms for retrieval. Optimal choice of the implementation therefore depends on the practical frequency of indexing/retrieval operations.

Figure 4.6 illustrates some retrieval results on different types of data (Zebrafish embryo from Marc Muller's lab, GIGA-Research, University of Liège; Thyroïd cytology data from Isabelle Salmon's lab, Université Libre de Bruxelles; Ovarian histology data from Carine Munaut's lab, GIGA-Research, ; University of Liège):

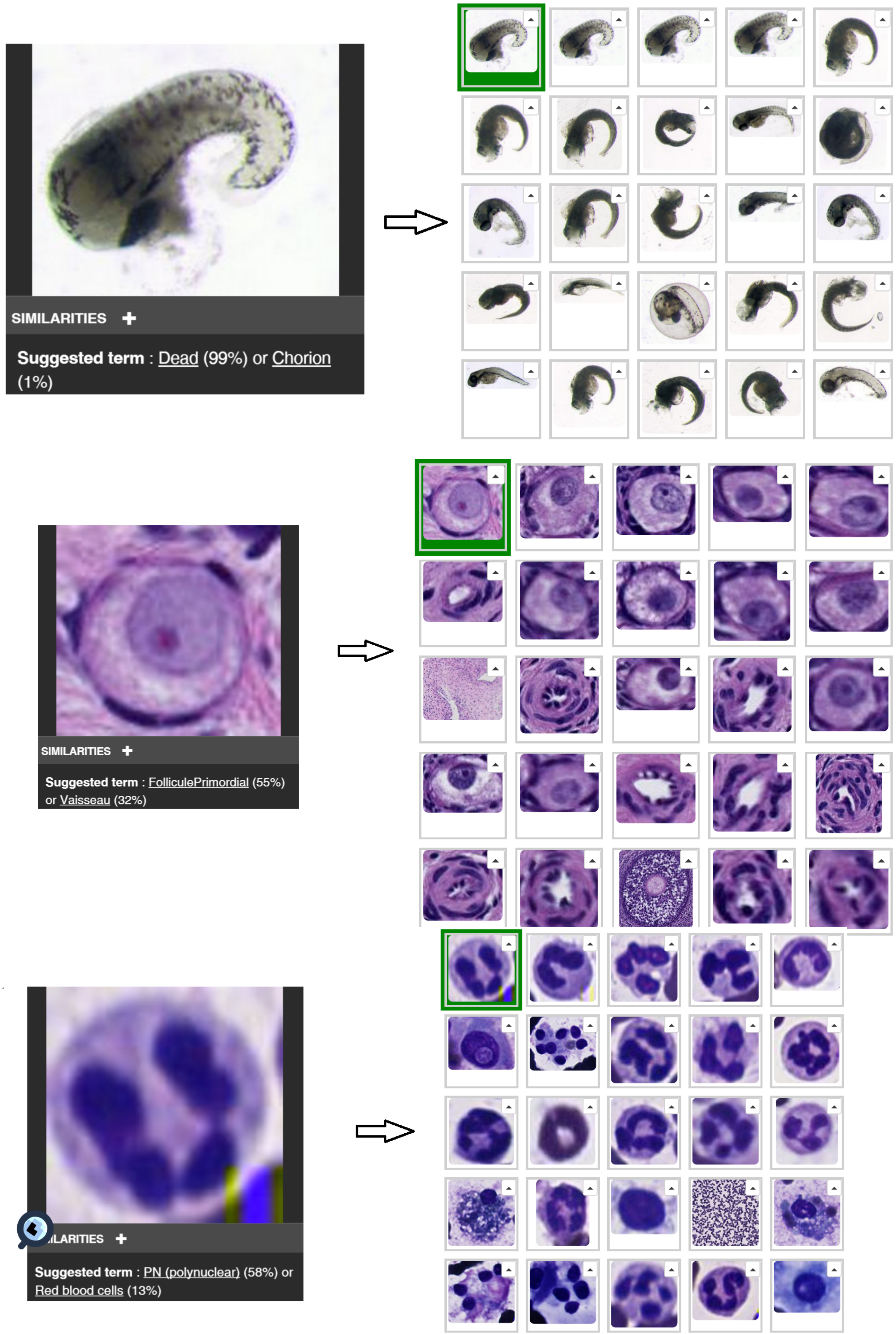

4.2.5 Detect sample and Object Finder

We provide here two illustrative examples of the "Detect sample" and "Object finder" modules that are based on standard image processing algorithms.

Figure 4.7 illustates the application of the detect sample module (image from the OpenSlide repository). This module was also used in our experiments for tissue tumor detection (see Suppl. Notes 4.2.2 and 4.2.4.1).

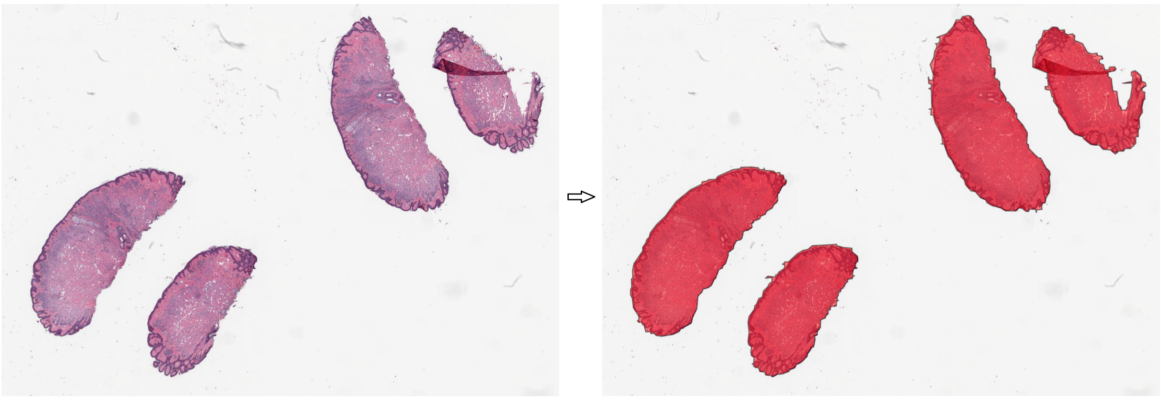

Figure 4.7: Example of tissue detection: original image (left) and detected geometries (right).

Figure 4.8 shows the application of the object finder module on a 10 gigapixel pollen images (from Maurice Streel, Geology Department, University of Liege, Belgium) that generates 49936 objects.

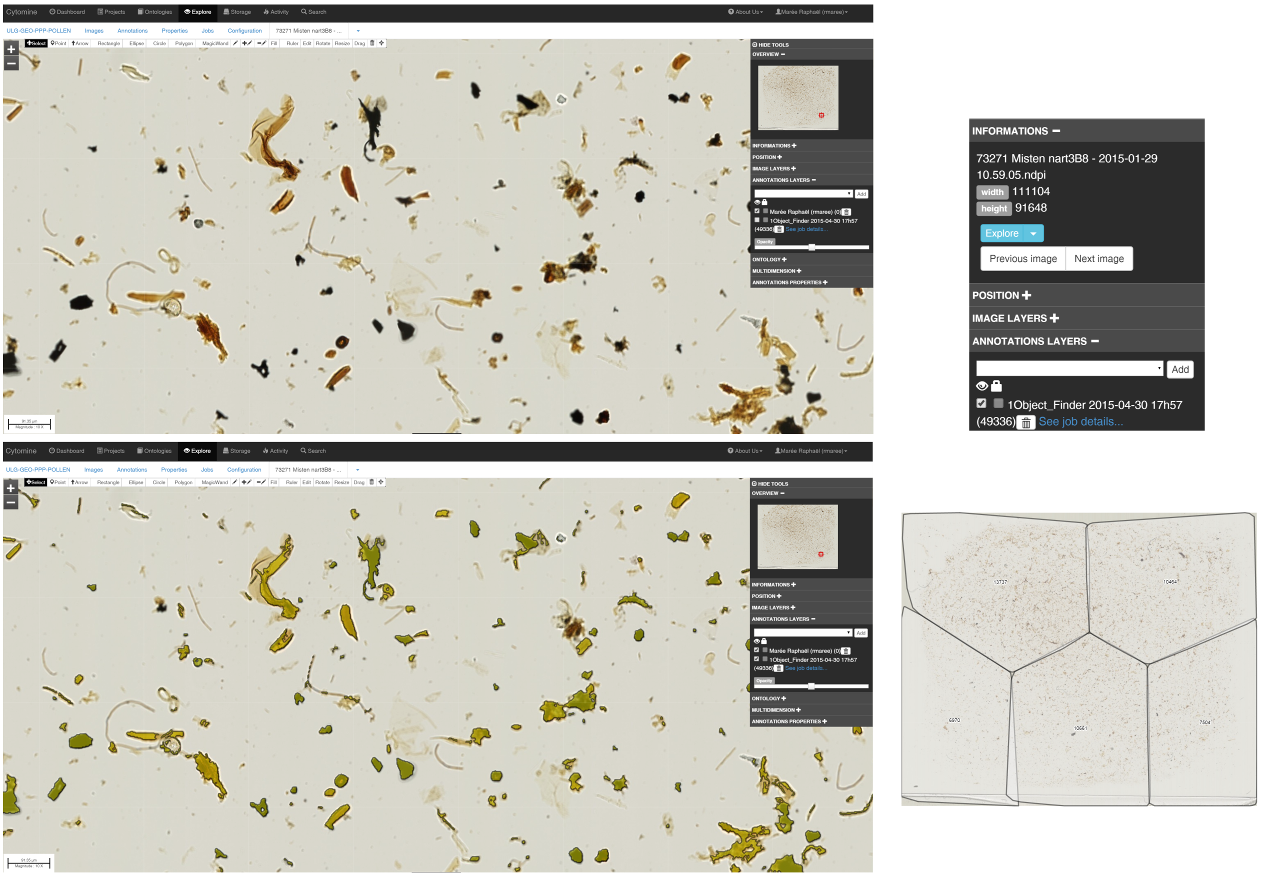

Figure 4.8: Example of the application of the object finder module on pollen images. Original area of the gigapixel image (top left). Overlay of detected objects in yellow (bottom left). Image and annotation informations (top right). Layer of detected objects displayed at lower resolution: When too much objects have to be displayed, we apply a clustering algorithm based on object spatial positions to group them into five clusters (with object counts) in order to avoid overloading the web browser (bottom right).

It is important to note that, as for other analysis modules, adapting parameter values or developing new detection algorithms might be needed for other types of images (e.g. images with lower contrast). Our architecture is extensible and allows to add novel software, register them to the Cytomine-Core and launch them from Cytomine-WebUI or through command line scripts.

Supplementary Note 5: User guide

This supplementary note covers the main functionalities of our software. The software can be tested at http://demo.cytomine.be/ with one of these 3 accounts, using a modern web browsers (Google Chrome (preferred choice), Safari, Mozilla Firefox):

**Username**	**Password**	**Role**
jsnow	jsnow	User
clannister	clannister	User
estark	estark	User

The software can also be installed automatically on desktop/server computers using the procedure described in Section 6.1. In both cases, our installation procedure installs "toy data", namely five projects for testing main functionalities.

This user guide will be continuously updated and available in the "User" documentation at http://doc.cytomine.be/

5.1 Installation

The automated installation procedure is described at: http://doc.cytomine.be/x/goCj
It basically requires the edition of a single configuration file (Bootstrap/configuration.sh) and the execution of a few command lines. The procedure will automatically create virtual environments (using Docker deployment system) for each Cytomine components and download, compile, and install all the required libraries, and start all servers. The whole process takes approximately 2 hours without user intervention. The web user interface is then directly accessible at the http://$CORE_URL$ location (e.g. http://demo.cytomine.be) using modern web browsers (we recommend using Google Chrome).

5.2 Usage (Cytomine-WebUI)

This guide explains how to:

- Visualize and annotate manually images
- Configure projects and manage users
- Upload new images
- Apply Cytomine-DataMining analysis modules and proofread them
- Perform textual search
- Follow online users
- Blind assessment (including IRIS: the Inter-observer study module)

In this guide, we assume using the "jsnow" account.
Please refer to your web browser documentation for basic navigation operations (e.g. "F5" key might be useful to reload pages).

5.2.1 Visualization and manual annotations

Users log into Cytomine using the login panel:

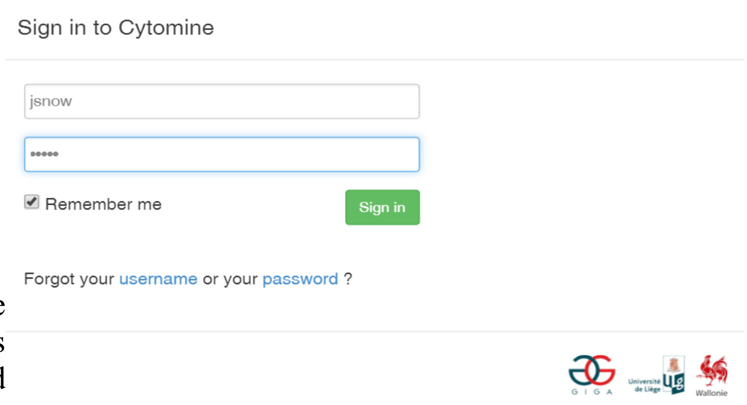

Once connected, the user has access to its user dashboard which summarizes global information (number of projects, images, annotations) and it also shows latest opened projects, images, and activities:

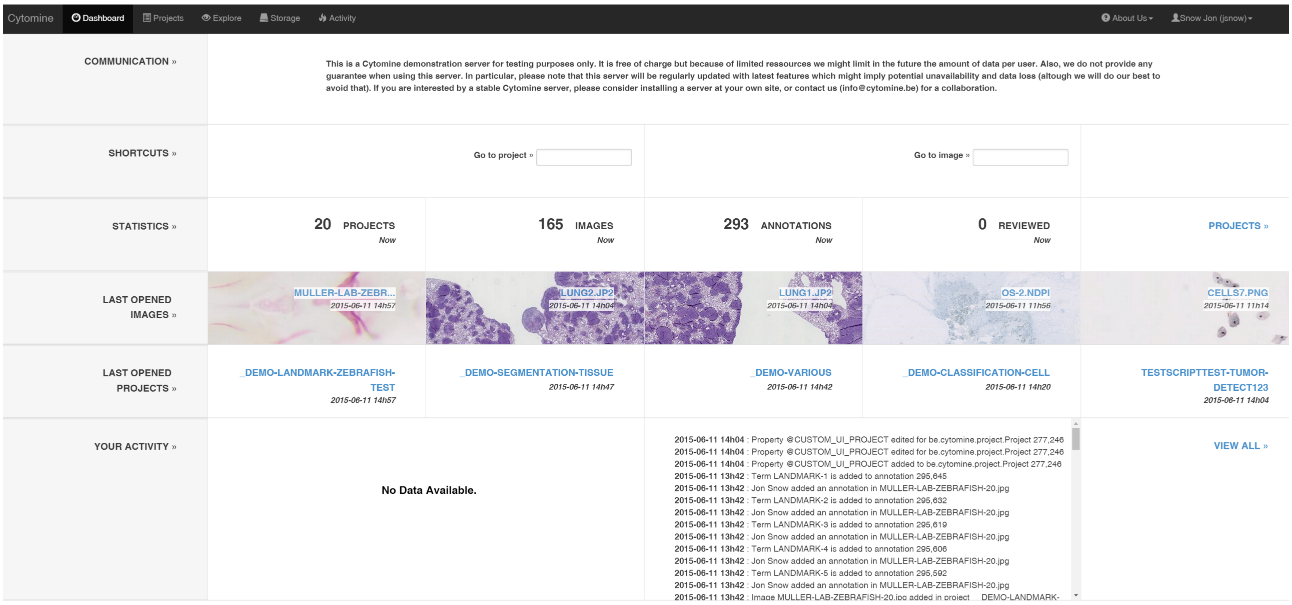

The menu on the top right (black bar) gives access to Account information (under the user name) where the user can change its password and get its public/private keys. The help sub-menu lists main shortcuts applicable when exploring images:

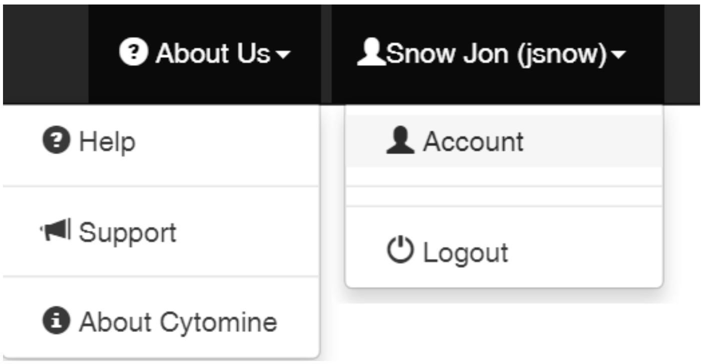

The menu on the top left (black bar) gives access to the User dashboard, the listing of Projects, the

Storage panel (to upload new images through the web interface), and the Activity panel which summarizes latest user activities:

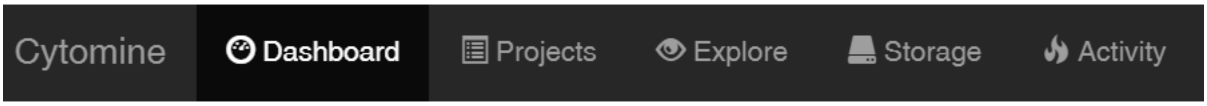

The project listing displays boxes where each box corresponds to a specific project. Project listing can be filtered (box at the top left) by names, number of annotations, images, disciplines, ontology names:

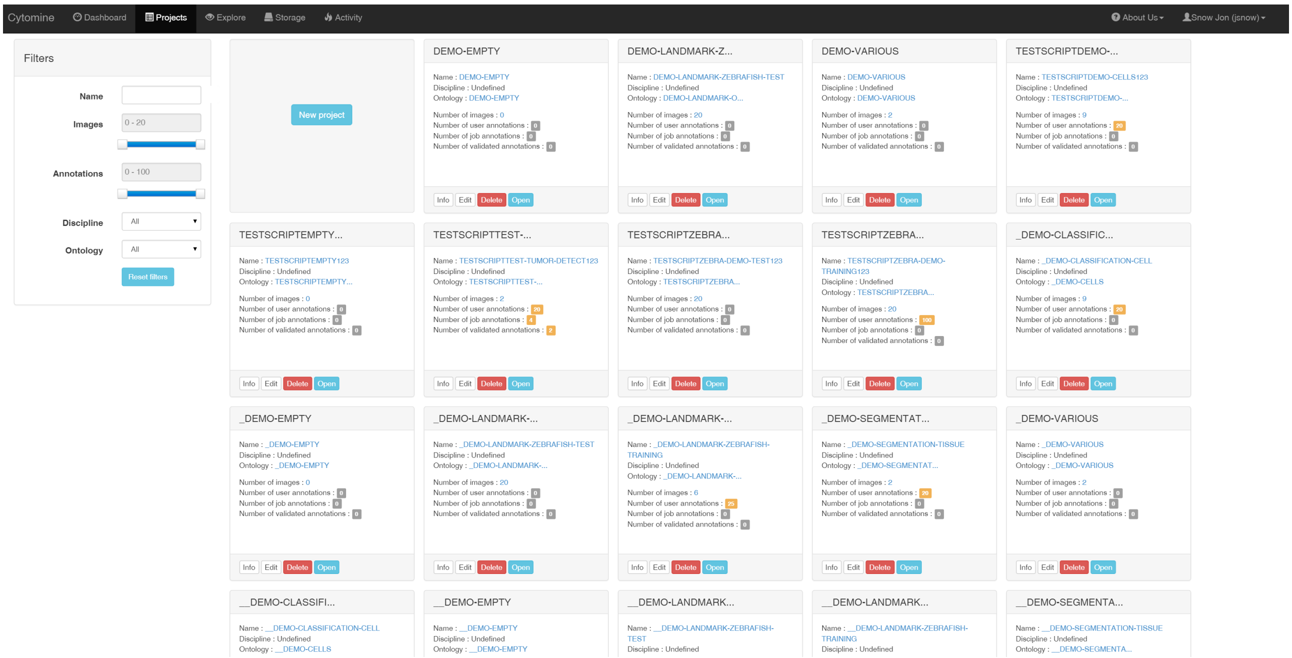

Clicking on the project name (e.g. _DEMO_SEGMENATION_TISSUE) or on the Open button will open the project and shows the Project dashboard with basic information (textual description on the top right, users currently online) and various statistics about the project (including number of images, annotations, various statistics of annotations by ontology terms, users, images, ...).

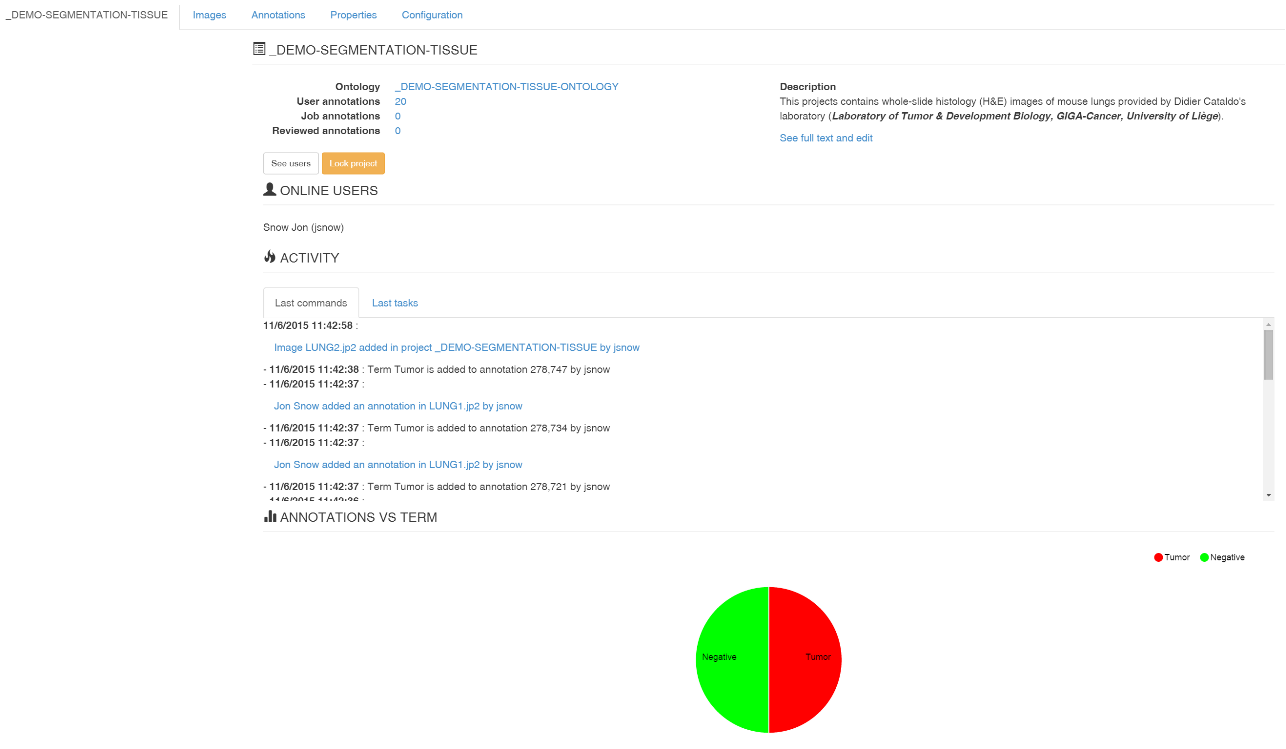

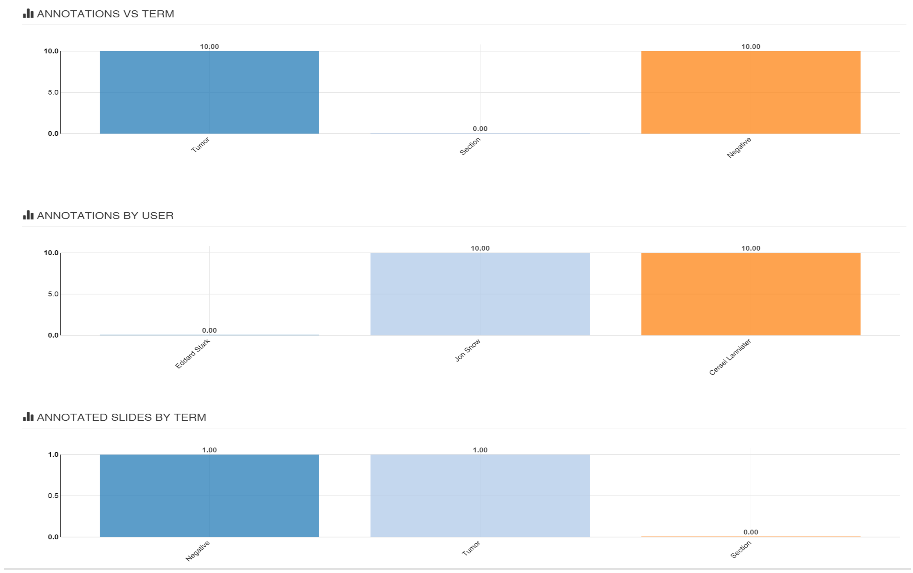

One key concept of Cytomine is the Ontology, a structured vocabulary of user-specified terms used for the semantic annotation of regions of interest in images. Each project has a single ontology. It can be either an existing ontology (it can be associated with the project when a user creates it) or a new ontology that can be edited online. Editing the ontology can be done by clicking on its name

(first line under the project name) which opens the Ontology editor. The editor allows to rename the ontology, add/edit/delete terms from the ontology. In this "toy" project where life scientists are interested to quantify the sizes of tumor islets with respect to the size of tissue sections, the Ontology is simplified and has only three terms: Section (to delimit the tissue section, in brown), Tumor (for tumoral islets, in red), and Negative (for all other tissue substructures, in green):

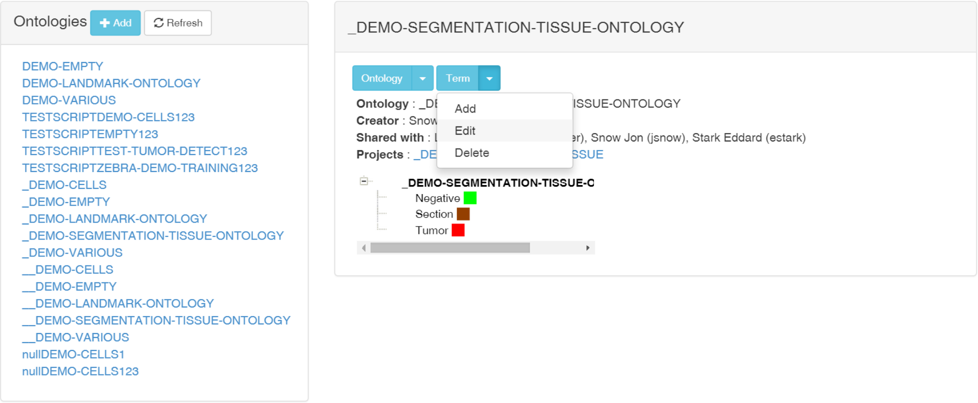

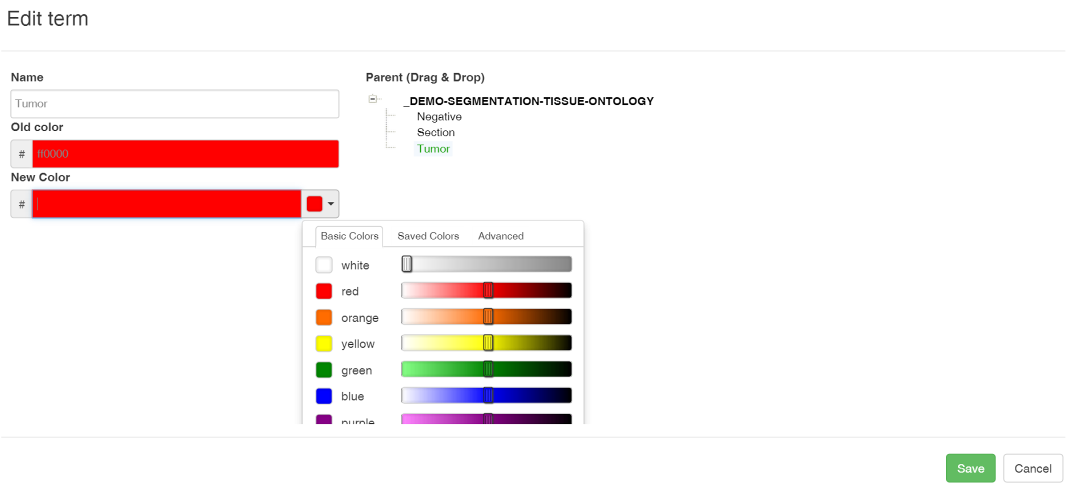

Once the ontology is defined, one can start semantically annotating whole-slide images. It has to be noted that Term names and colors can be changed afterwards, only term identifiers are linked to annotations in the database. To start annotation, the user goes back to the Project dashboard and clicks on the "Images" tab which opens a page with the Image listing of this project:

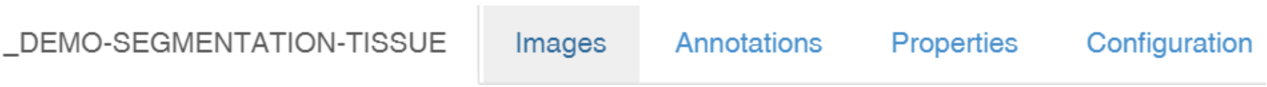

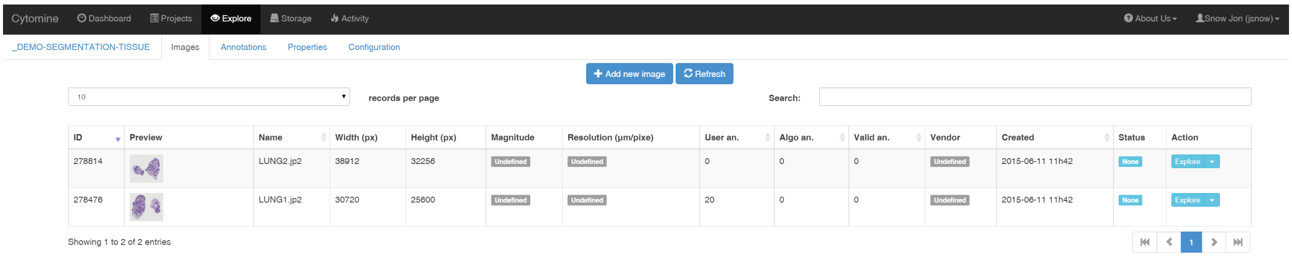

This project contains only 2 images whose characteristics are listed. Image "LUNG1.jp2" already

contains 20 user annotations. One can open the image by clicking on its thumbnail or on the blue Explore button at the right. Note that the arrow at the right of the Explore button gives access to supplementary information and operations (e.g. download the image, describe it, start reviewing it (see below), importing annotations from another project, ...). The explore view is then displayed.
It is a zoomable viewer for gigapixel images, with various tools for annotation. Note that multiple images can be opened in parallel, each one having its own tab. By default (this can be configured in the "Configuration" tab of the project), the viewer also displays current user's annotations (in red here, corresponding to the color of the Tumor term):

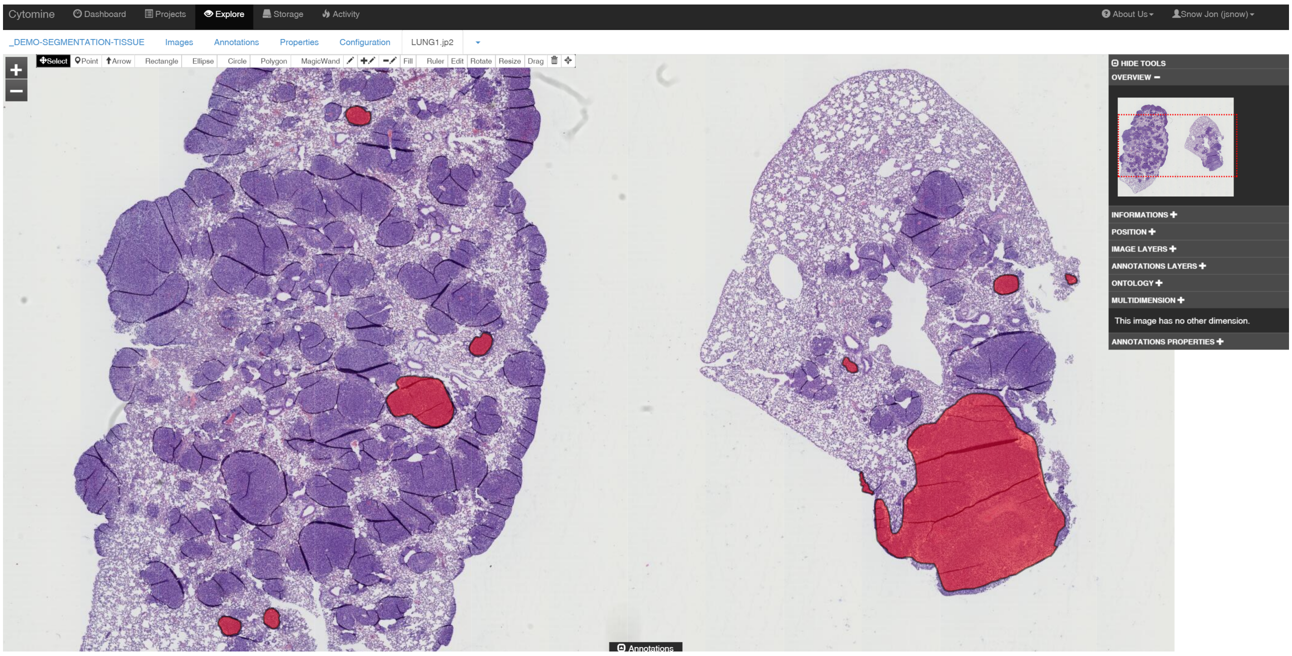

Annotations can be selected 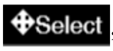

, edited, and drawn manually by using tools on the top bar:

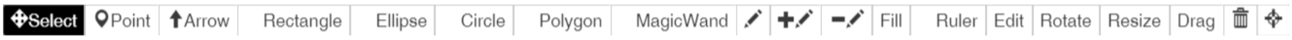

It includes geometries such as:
- Point, Rectangle, Ellipse, Circle, Polygon,
- Freehand
- Magic Wand (which parameters have to be configured in Project Configuration tab)

And operations such as:
- Rotate, Resize, Drag,
- Delete
- Fill
- Complement (performs the union of a new geometry with an intersecting, existing, geometry)
- Subtract (subtracts the intersection of a new geometry with existing geometry)

Selecting an annotation opens the Current Selection Annotation panel (at the bottom left) which gives basic information (e.g. annotation area based on image resolution) about the currently selected annotation and launch the content-based image retrieval algorithm. This algorithm suggests ontology terms based on visual similarity. The most similar annotations can be visualized by clicking on "See Similar Annotations" (they are ranked according to their computed similarity):

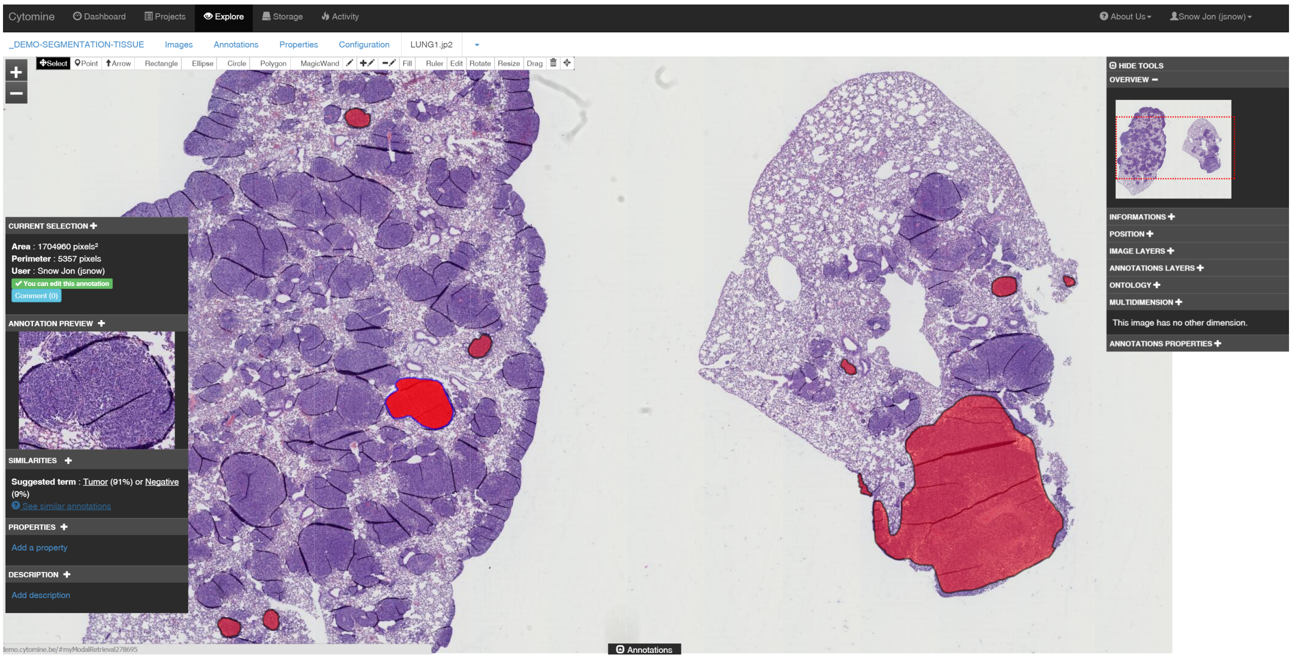

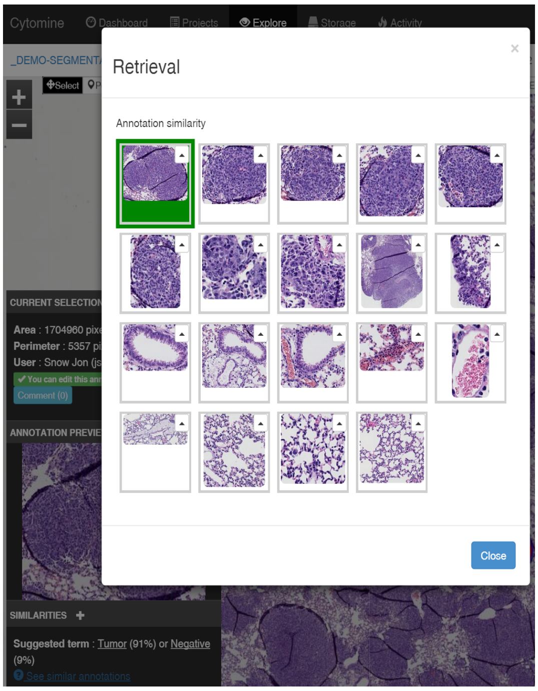

It is possible to click on these "similar" annotations to jump directly at their locations within their original image.

Properties (key-value pairs) and keywords can be added to Annotations by clicking on the "Add property" link (at the bottom of the Current Selection panel).

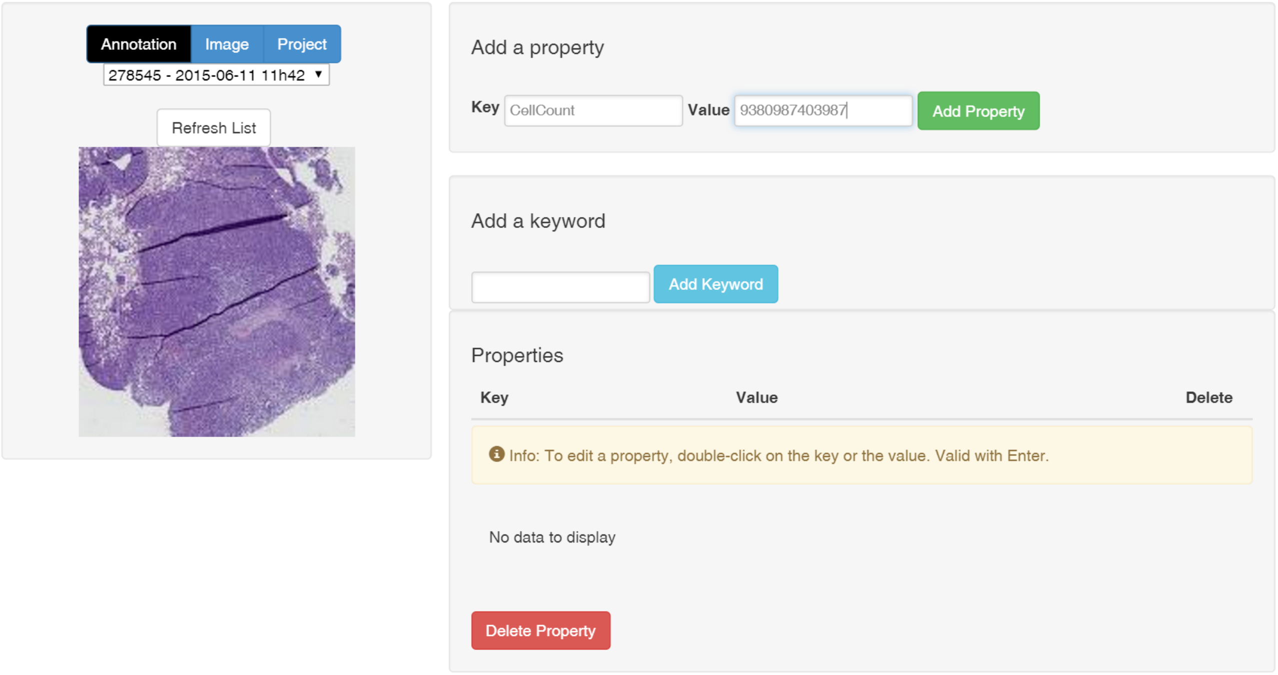

Rich text descriptions can also be added in a similar way as for Projects and Images.

On the right side of the Explore view, the "Tools" panel can be activated to show an overview (thumbnail with red square corresponding to the active view), image information, image layers (which allow to apply on the fly image processing filter to tiles, see Configuration page of the project), annotation layers (containing user layers, userjob layers, and the review layer), ontology, and multi-dimensional browser.

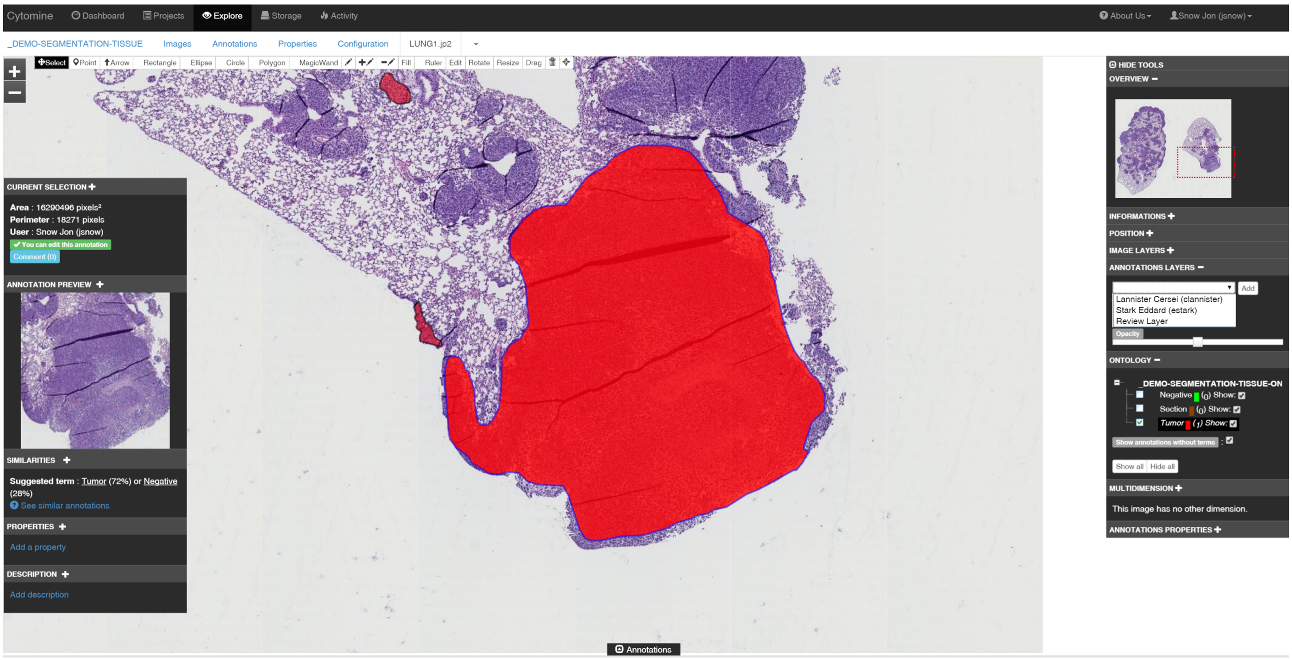

Selecting and adding another user layer will display its layer of annotations in the whole-slide images in addition to current user annotations (in this case, the user clannister only created

annotations with the Negative term, in green):

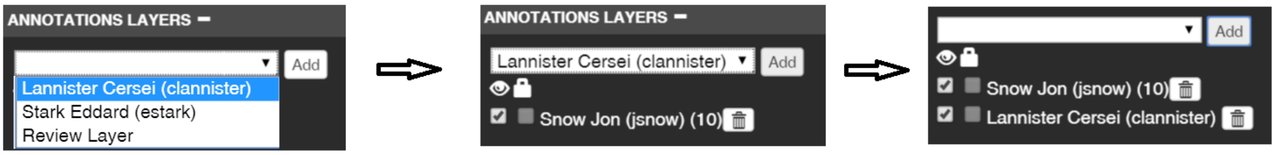

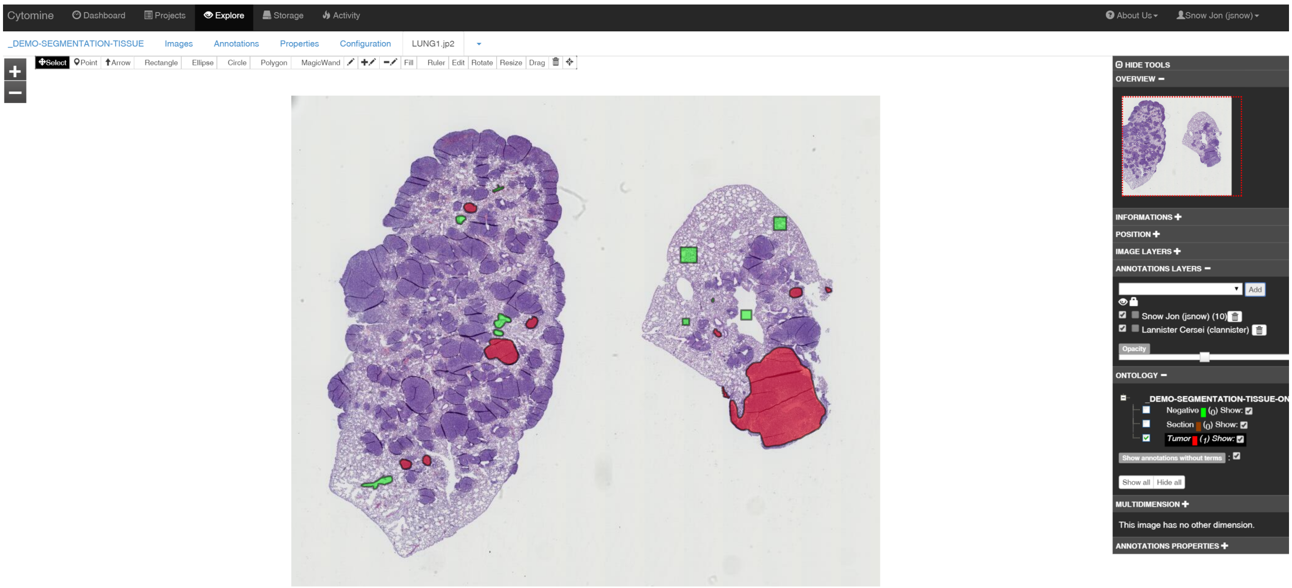

Multiple user annotations can then be displayed. A blinded mode allows to hide other user layers if needed (see Project Configuration page).

Adding an annotation requires the user to draw it, then eventually associate a term from the Ontology (the color of the Annotation thus changes):

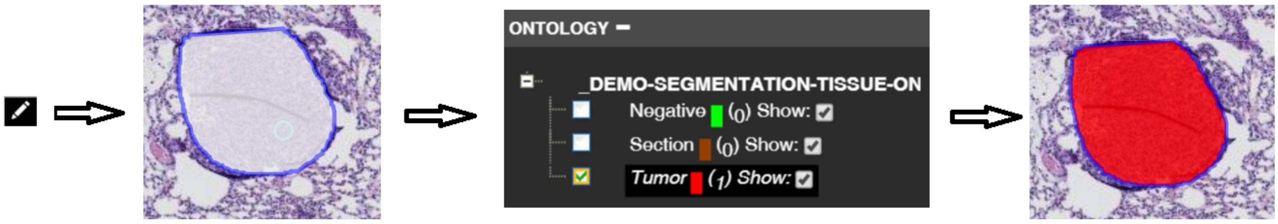

All annotations created in a project are visible in filtered galleries in the Project Annotations tab:

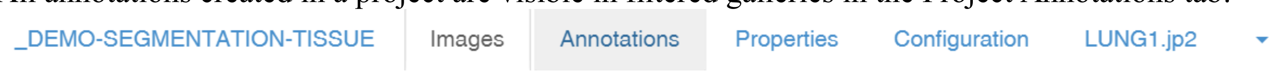

Annotations can be filtered according to images they come from, terms from the Ontology, Users from the project and types (user/job/reviewed). Filters can be saved and reused later. The user can click on any of these annotations to jump to its actual location in whole slide images.

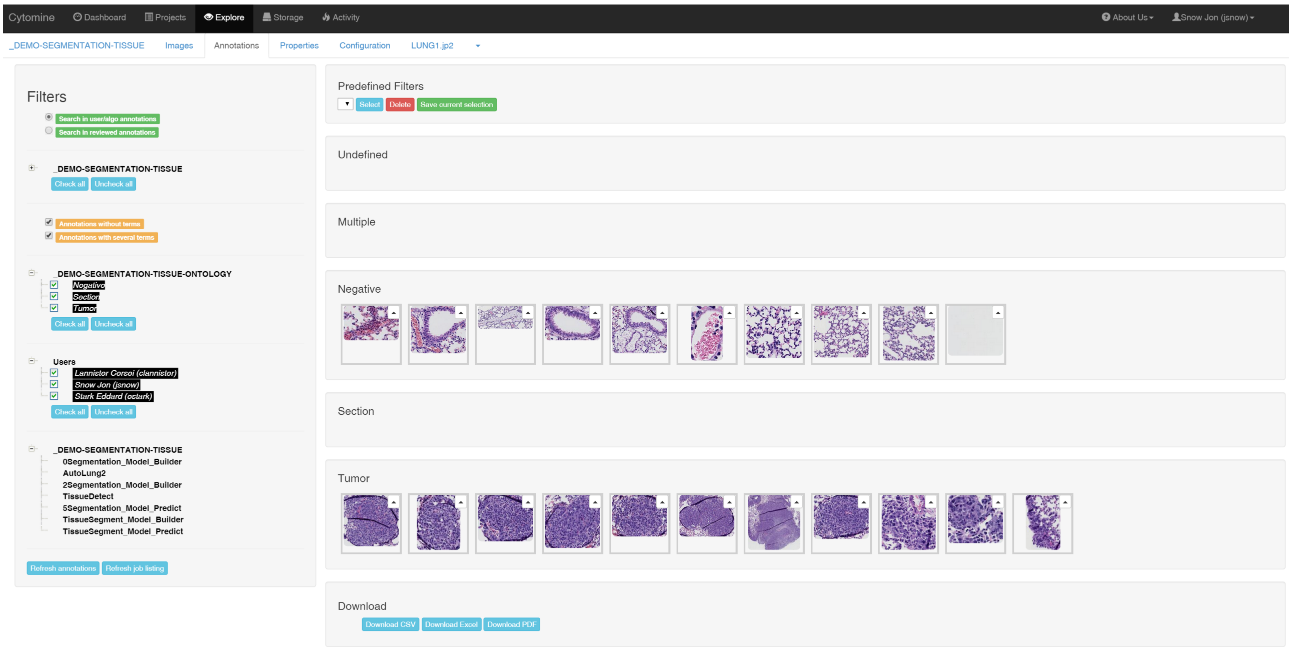

Annotation descriptions can be exported as tabular files containing annotation information (area, user who created them, direct link to it,...):

	A	B	C	D	E	F	G	H	I	J	K
1	Id	Area (micronsÂ²)	Perimeter (mm)	X	Y	Image Id	Image Filename	User	Term	View annotation picture	View annotation on image
2	302661	554624.0	2958.0	7046.116321532636	4725.001467744019	278476	LUNG1.jp2	jsnow	Tumor	http://demo.cytomine.be/api/annotation/302661/crop.png	http://demo.cytomine.be/#tabs-image-278366-278476-302661
3	278747	175230.0	1594.0	7069.32970381784	6572.665609009112	278476	LUNG1.jp2	jsnow	Tumor	http://demo.cytomine.be/api/annotation/278747/crop.png	http://demo.cytomine.be/#tabs-image-278366-278476-278747
4	278734	227312.0	1880.0	5960.75249290256	6380.173060228526	278476	LUNG1.jp2	jsnow	Tumor	http://demo.cytomine.be/api/annotation/278734/crop.png	http://demo.cytomine.be/#tabs-image-278366-278476-278734
5	278721	253480.0	1946.0	12556.091504918204	13743.268818052706	278476	LUNG1.jp2	jsnow	Tumor	http://demo.cytomine.be/api/annotation/278721/crop.png	http://demo.cytomine.be/#tabs-image-278366-278476-278721
6	278708	251696.0	1973.0	9331.685970376962	19742.053609645496	278476	LUNG1.jp2	jsnow	Tumor	http://demo.cytomine.be/api/annotation/278708/crop.png	http://demo.cytomine.be/#tabs-image-278366-278476-278708
7	278695	1704960.0	5357.0	11001.564964964966	12278.455655655656	278476	LUNG1.jp2	jsnow	Tumor	http://demo.cytomine.be/api/annotation/278695/crop.png	http://demo.cytomine.be/#tabs-image-278366-278476-278695
8	278682	71540.0	1605.0	10794.882024042494	20723.60314975305	278476	LUNG1.jp2	clannister	Negative	http://demo.cytomine.be/api/annotation/278682/crop.png	http://demo.cytomine.be/#tabs-image-278366-278476-278682
9	278669	162990.0	1568.0	8824.732830234983	19137.8159396282	278476	LUNG1.jp2	clannister	Negative	http://demo.cytomine.be/api/annotation/278669/crop.png	http://demo.cytomine.be/#tabs-image-278366-278476-278669
10	278656	434896.0	4195.0	4590.964963270912	5427.768220448107	278476	LUNG1.jp2	clannister	Negative	http://demo.cytomine.be/api/annotation/278656/crop.png	http://demo.cytomine.be/#tabs-image-278366-278476-278656
11	278643	129030.0	1419.0	10797.548818104317	13221.16981580511	278476	LUNG1.jp2	clannister	Negative	http://demo.cytomine.be/api/annotation/278643/crop.png	http://demo.cytomine.be/#tabs-image-278366-278476-278643
12	278630	373352.0	2724.0	10976.037383845094	13842.707636046769	278476	LUNG1.jp2	clannister	Negative	http://demo.cytomine.be/api/annotation/278630/crop.png	http://demo.cytomine.be/#tabs-image-278366-278476-278630
13	278617	14602.0	527.0	21993.5566817331	14925.346299593662	278476	LUNG1.jp2	clannister	Negative	http://demo.cytomine.be/api/annotation/278617/crop.png	http://demo.cytomine.be/#tabs-image-278366-278476-278617
14	278604	117600.0	1372.0	20585.0	13798.0	278476	LUNG1.jp2	clannister	Negative	http://demo.cytomine.be/api/annotation/278604/crop.png	http://demo.cytomine.be/#tabs-image-278366-278476-278604
15	278591	681792.0	3304.0	20730.0	17282.0	278476	LUNG1.jp2	clannister	Negative	http://demo.cytomine.be/api/annotation/278591/crop.png	http://demo.cytomine.be/#tabs-image-278366-278476-278591
16	278578	443232.0	2664.0	25496.0	18924.0	278476	LUNG1.jp2	clannister	Negative	http://demo.cytomine.be/api/annotation/278578/crop.png	http://demo.cytomine.be/#tabs-image-278366-278476-278578
17	278564	266760.0	2068.0	23736.0	14159.0	278476	LUNG1.jp2	clannister	Negative	http://demo.cytomine.be/api/annotation/278564/crop.png	http://demo.cytomine.be/#tabs-image-278366-278476-278564
18	278545	1.6290496E7	18271.0	25481.181634248584	9723.086243496413	278476	LUNG1.jp2	jsnow	Tumor	http://demo.cytomine.be/api/annotation/278545/crop.png	http://demo.cytomine.be/#tabs-image-278366-278476-278545
19	278532	279788.0	1982.0	26318.944643801737	15324.092674930067	278476	LUNG1.jp2	jsnow	Tumor	http://demo.cytomine.be/api/annotation/278532/crop.png	http://demo.cytomine.be/#tabs-image-278366-278476-278532
20	278519	96759.0	1279.0	22222.987084750086	13198.883933277524	278476	LUNG1.jp2	jsnow	Tumor	http://demo.cytomine.be/api/annotation/278519/crop.png	http://demo.cytomine.be/#tabs-image-278366-278476-278519
21	278506	57715.0	942.0	28014.469363828004	15463.157382540645	278476	LUNG1.jp2	jsnow	Tumor	http://demo.cytomine.be/api/annotation/278506/crop.png	http://demo.cytomine.be/#tabs-image-278366-278476-278506
22	278492	76746.0	1585.0	22618.569549137515	10065.900478421101	278476	LUNG1.jp2	jsnow	Tumor	http://demo.cytomine.be/api/annotation/278492/crop.png	http://demo.cytomine.be/#tabs-image-278366-278476-278492

In addition to ontology terms, properties, keywords, and rich text descriptions, Comments can be associated to Annotations and send through e-mail to project's users (using e-mail addresses encoded in the User account). E-mails contain the textual comment and a direct link to the actual location of the annotation within its gigapixel image for direct visualization. The user has to click on the blue "Comment" button in the Current selection panel, then select recipients and then click on the green "Share" button:

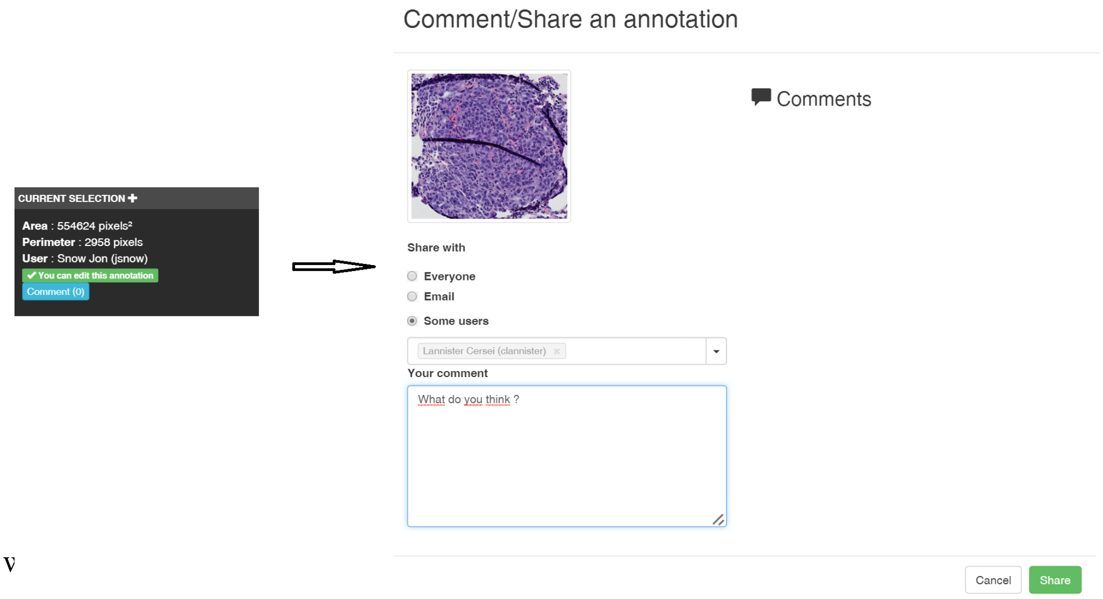

5.2.2 Project configuration and user managment

Each project can be configured through the Configuration page:

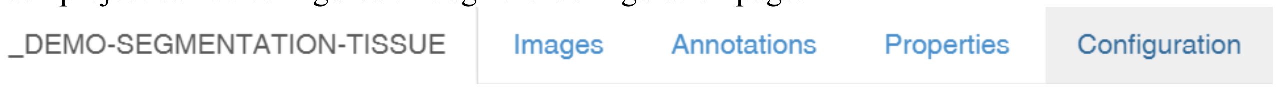

Admins of a project can configure all options including general configuration (e.g. blind mode which hides image names, enable/disable the content-based image retrieval algorithm,...), user management (add/delete users from this project), configure the default annotation layer in the Explore view, customize the user interface to enable/disable graphical tools and displayed information (such as the drawing tools and panels), the image filters (that can be applied on-the-fly to image tiles), the softwares to be associated (e.g. the Cytomine-DataMining modules or any other third-party softwares registered to the database), and some other visual configuration (e.g. the magic wand tolerance parameter or the size of Point annotations, configurable by a regular User).

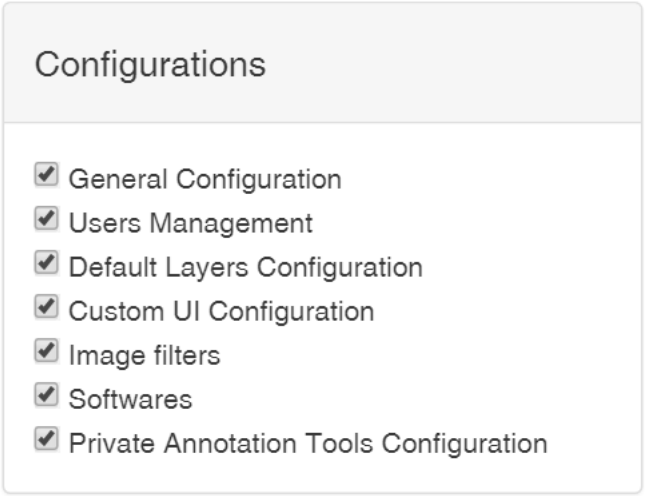

An existing user in the database can be added to the Project by an admin.

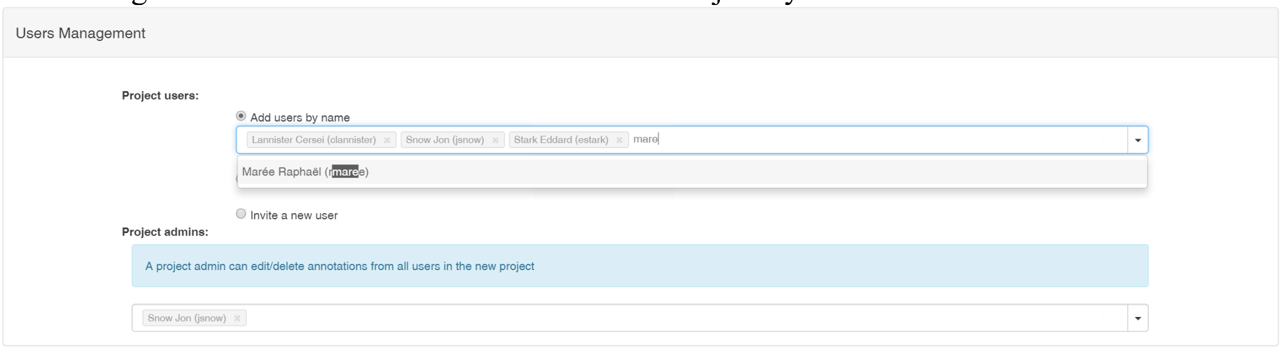

To add new user into the database through the web user interface, admins or super admins have to enter into the Cytomine admin area where the can add new user or edit existing ones (including their role):

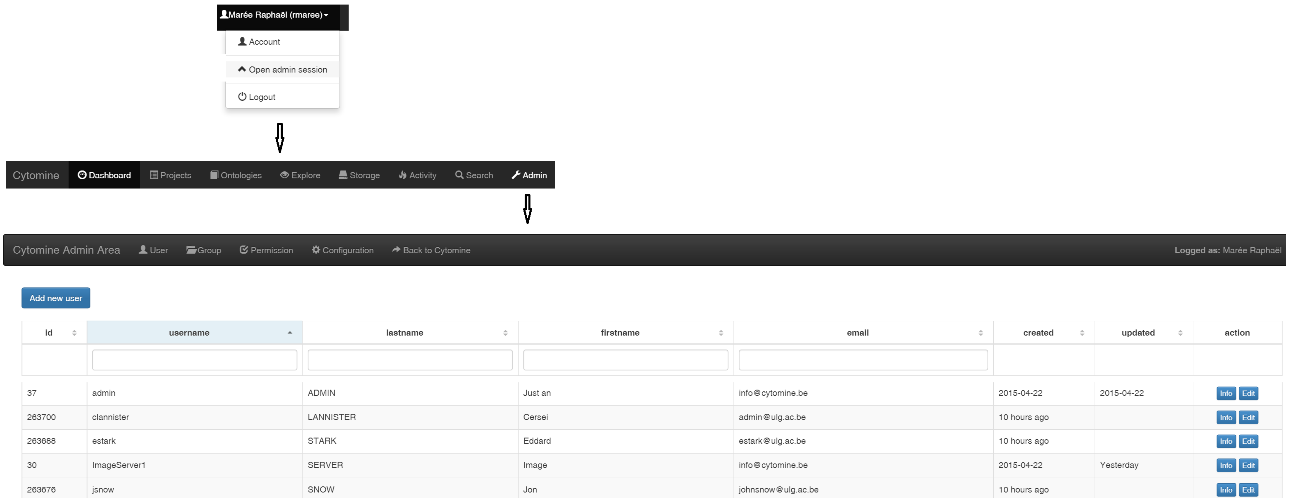

Note: on our demo instance, we do not provide admin codes to avoid issues due to unintentional operations. If you install the software on your servers, an admin user will be created by our automated installation procedure (username/password is requested during installation).

5.2.3 Upload and manage images

A user can upload images to its storage and then associate images to project(s). The user has to click on the "Storage" button (top black bar) to access the Storage panel where he can select (drag & drop or "Add files" dialog box) images from its local computer and link them automatically to a given project. If multiple files are to be uploaded, start upload will upload five of them in parallel:

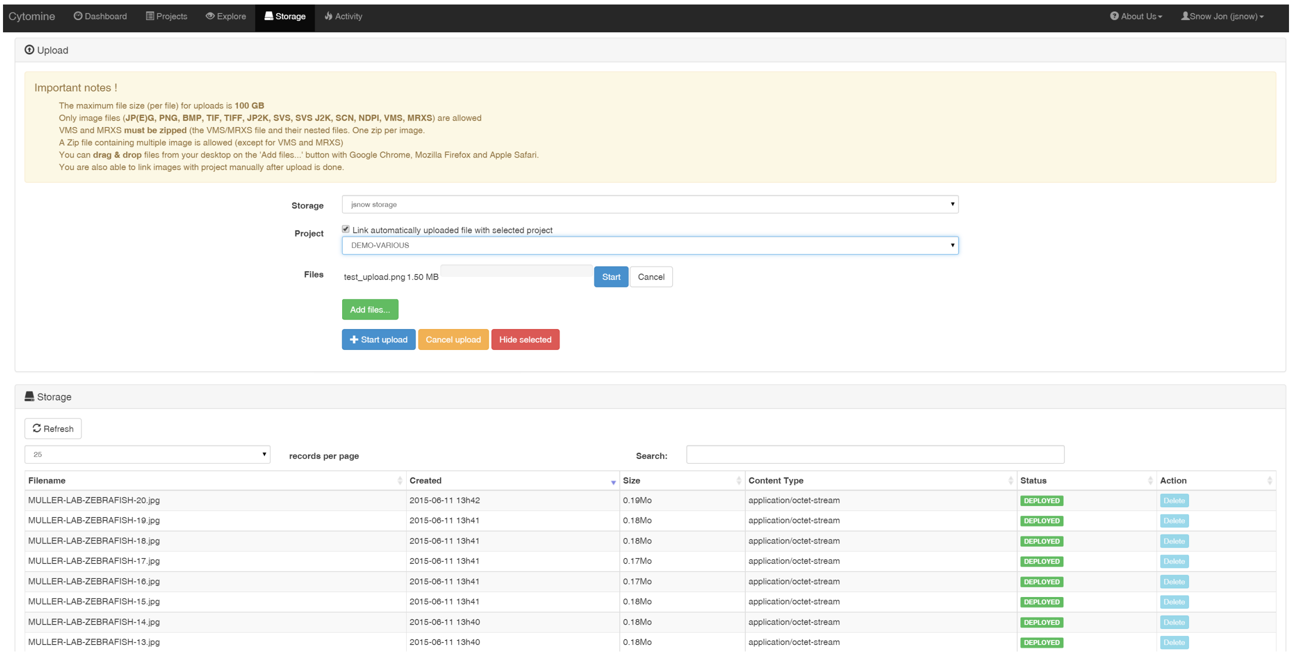

The bottom table lists all images uploaded to the Cytomine instance and their current status. Once it is "Deployed", an image will appear in the user storage and selected (if any) project image list. Some images require additional conversion steps (e.g. non-pyramidal TIFF, or multidimensional microscopy formats supported by Bio-Formats) and will take longer than natively supported formats. The uploaded image will also be available for other projects and the User can associate it

to another project through the "Add Image" (blue) button in the specific Project image list:

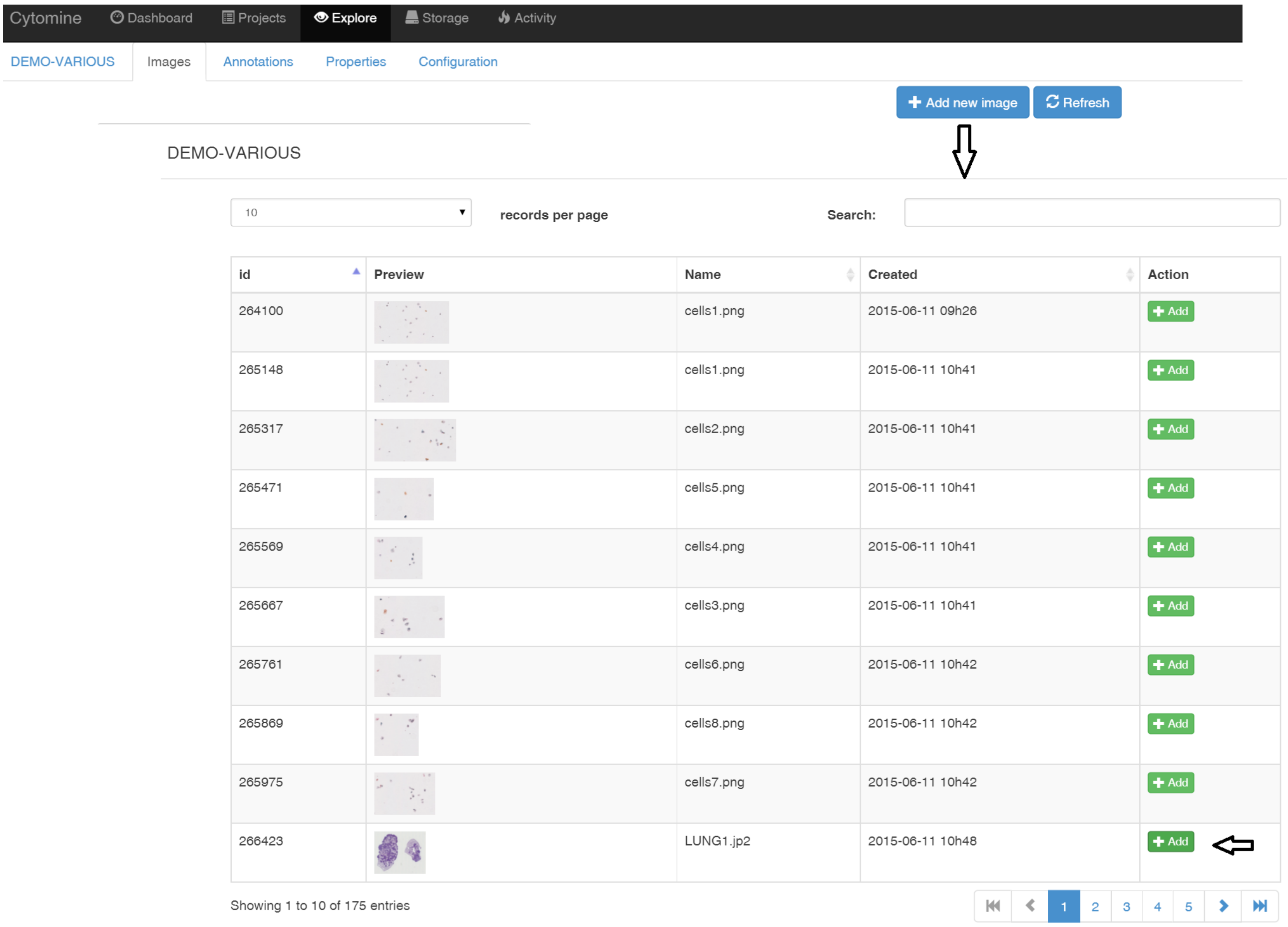

An image can be removed from a project through the "Delete image" option in the drop down list (Arrow at the right of the explore button):

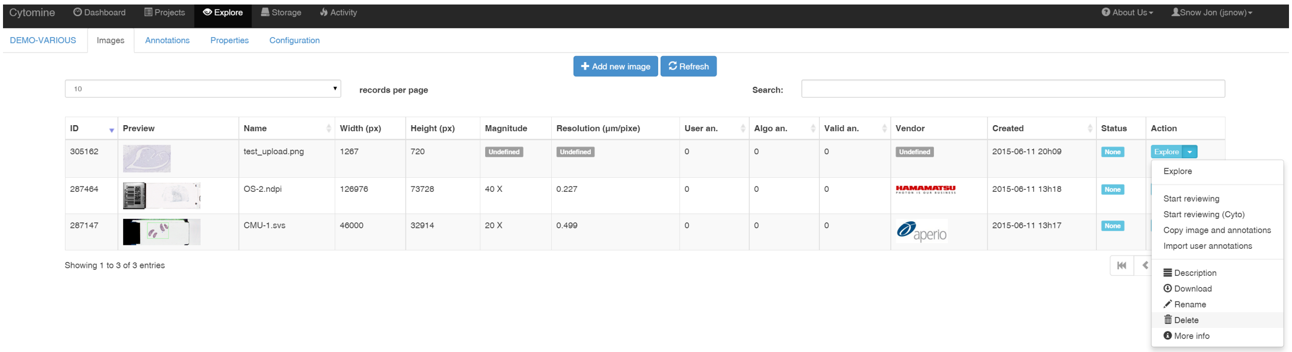

5.2.4 Apply Cytomine-DataMining analysis modules and proofreading

Our Cytomine-DataMining analysis modules can be launched from Cytomine-WebUI. It has to be noted that other third-party modules can be similarly launched, provided they are registered as Software into the Cytomine-Core and described by a template of Software parameters.

Here we only illustrate the basic principles of these workflows on small ("toy") datasets. Obtaining satisfactory recognition performances on real-world applications depends on many factors including image variations (due to image acquisition and sample preparation protocols), and the quality and quantity of annotations provided for training. To obtain validated training sets by multiple experts, one might use the Inter-observer module (see below Section 6.2.5). Overall, our tool allows to rapidly prototype such applications by using our generic algorithms described in Supplementary Note 4. Additional research in machine learning and image analysis might produce algorithms better suited for specific applications. Our modules allow to build models by tree-based supervised learning, apply them on new images, edit results, and re-apply learning procedures based on enhanced training sets.

6.2.4.1 Tissue Detection and Semantic segmentation

In this example, we want to quantify the size (area) of tumor islets with respect to section sizes in whole slide (gigapixel) images. In order to produce these quantification results, we will apply four registered softwares: a Tissue Section Detector, a Tumor detector learning procedure, a Tumor detector prediction procedure, and a final procedure to output statistics (ComputerTermArea). We use the toy project DEMO-SEGMENTATION-TISSUE.

The first step (if not already done) is to associate the Software to the Project in the Project Configuration page (bottom of the page), in our case we begin with the TissueDetect software:

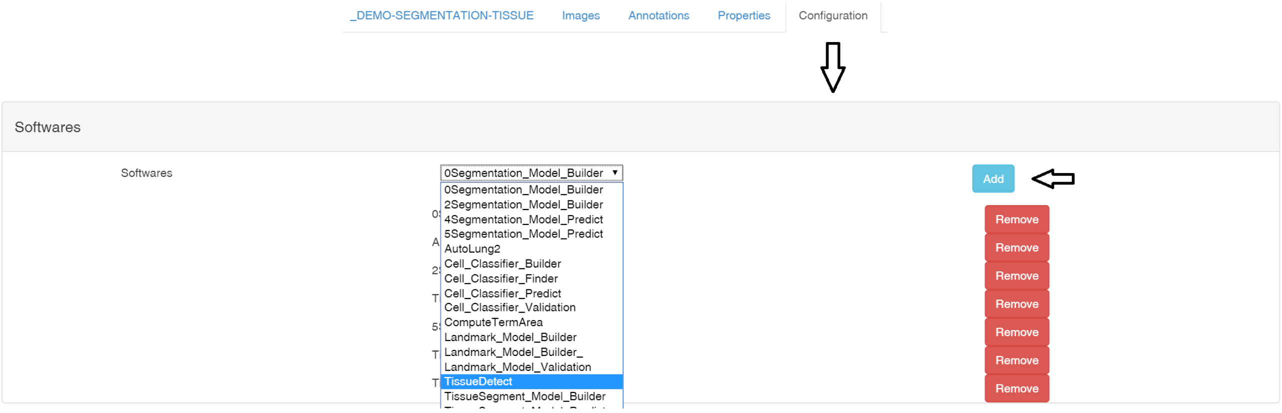

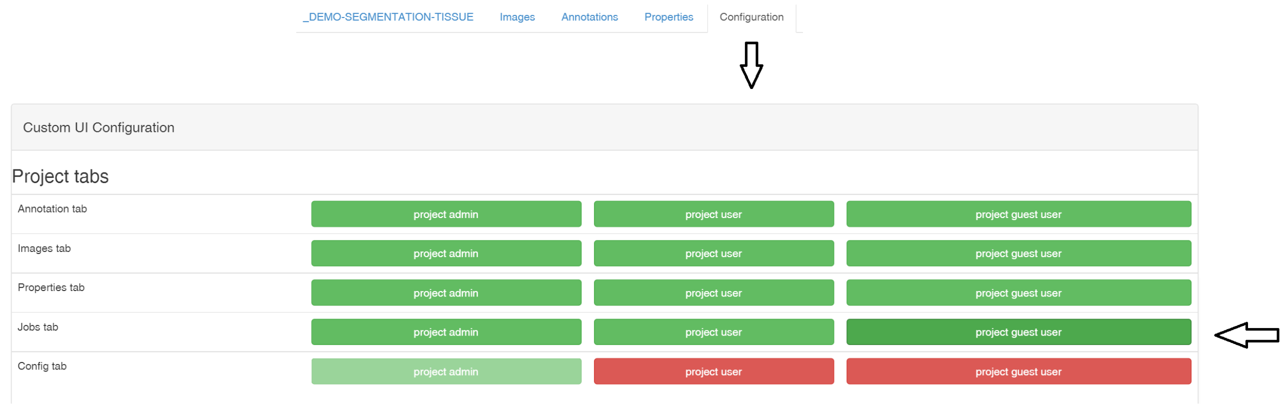

The user also has to activate (green) the "Jobs" tab in the "Custom UI" panel (if not already done): Now let's consider the User wants to apply the "Detect Sample" analysis module to detect its tissue sections. The user first click on the "Jobs" tab of the project, then on the "TissueDetect" software, then on the "Run Job":

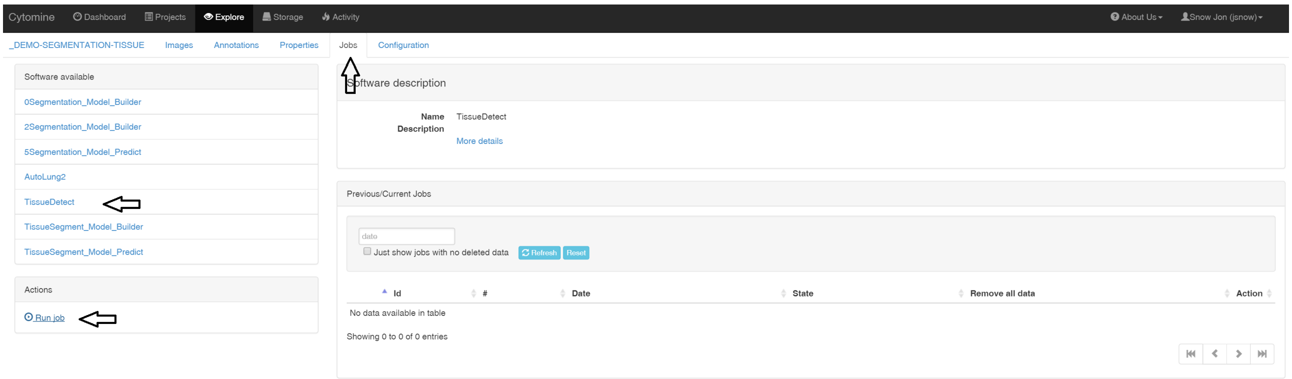

It opens a "Launch new job" dialog box to allow the User to configure the Software parameter values (the form is automatically build based on software parameter definition in the database). Here, we want to associate the term "Section" to objects detected by the procedure, and we keep default values for the thresholding algorithm:

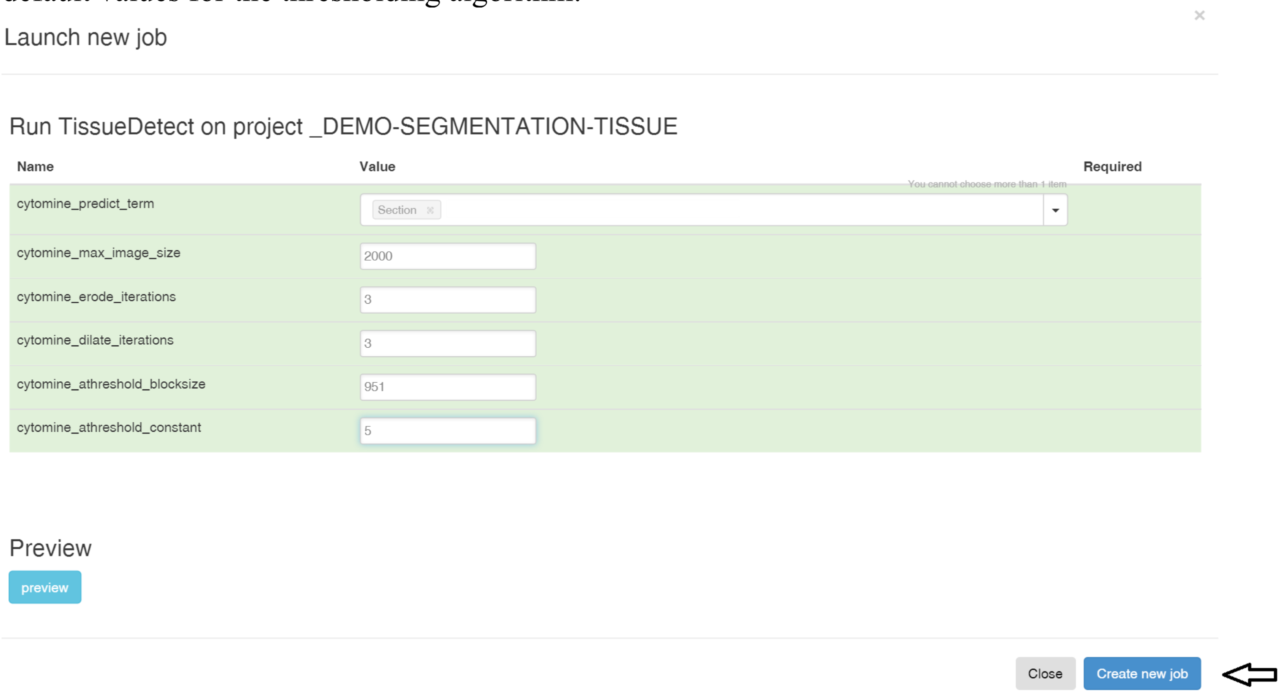

This will add an entry to the "Previous/Current jobs" panel. The job will be executed and Status is

displayed and updated during execution. It can be seen by clicking on the "Details" blue button. This also displays parameter values of the running job:

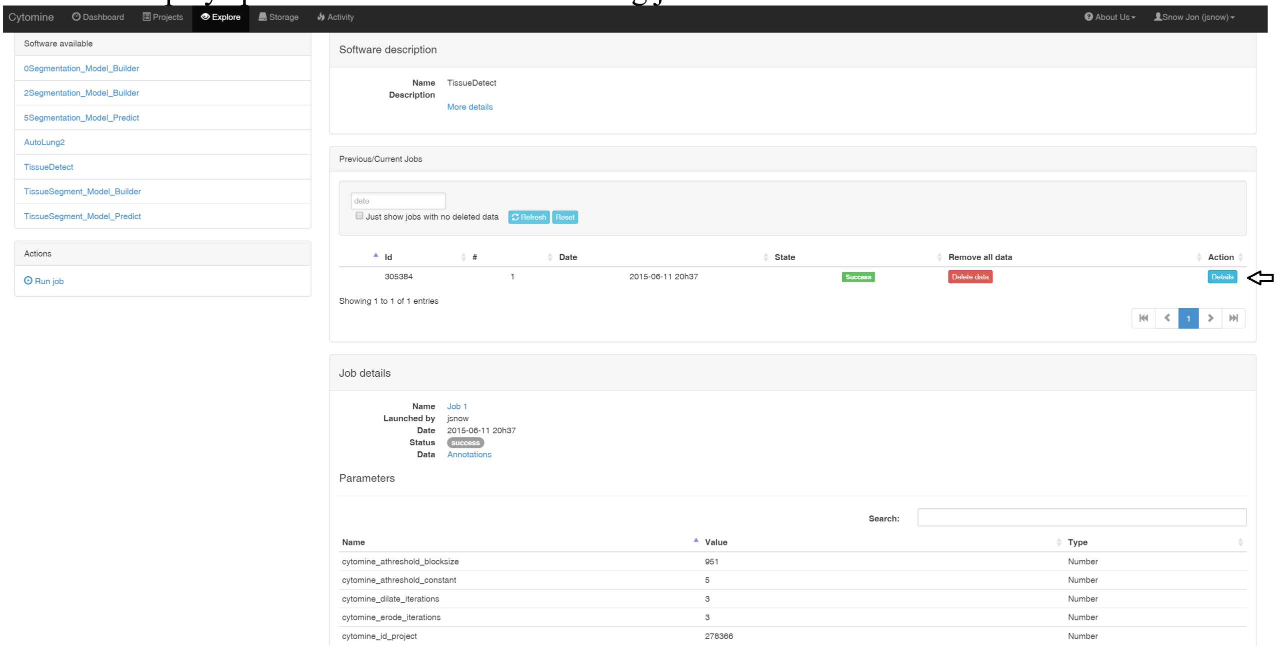

Once the job has reached the "Success" status (in roughly one minute for the two images of our toy project running on our demo instance), one can see (by scrolling down) the objects detected by this job by clicking on "View Predicted galleries" (blue button) that will open the Annotations tab with the filter corresponding to this UserJob. In our case, Sections of the lung are well detected automatically:

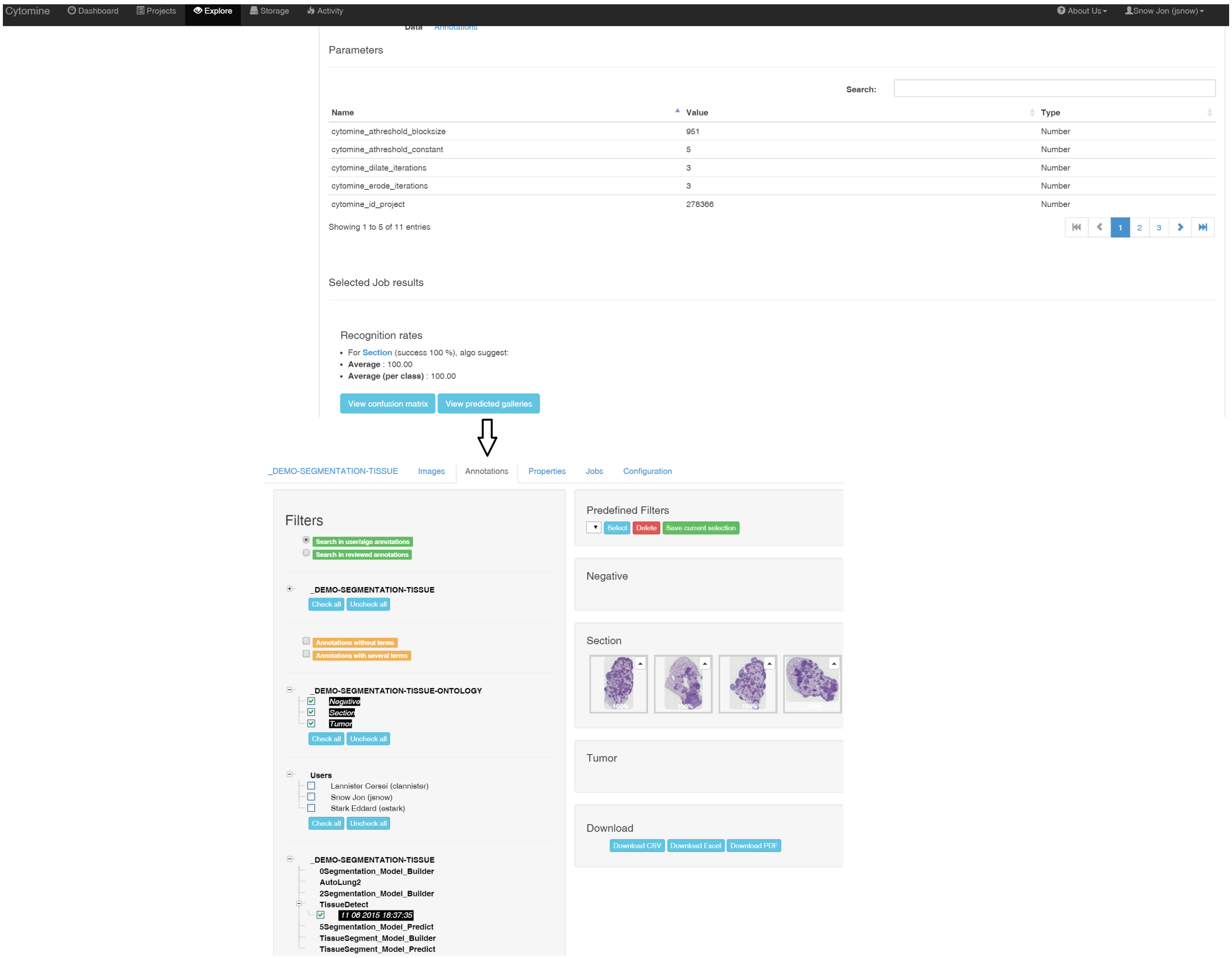

The user can then validate annotations produced by this module in all project images by using proofreading (Review) tool from the Project Image listing:

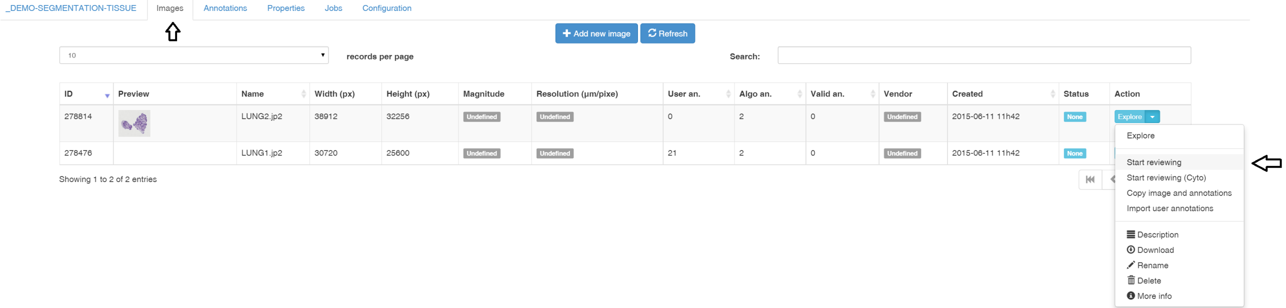

"Start reviewing" (using LUNG2 image) opens the Explore view with additional "Review" tools in the right Panel. The user can then first select the Job (or User) layer from which he wants to validate annotations:

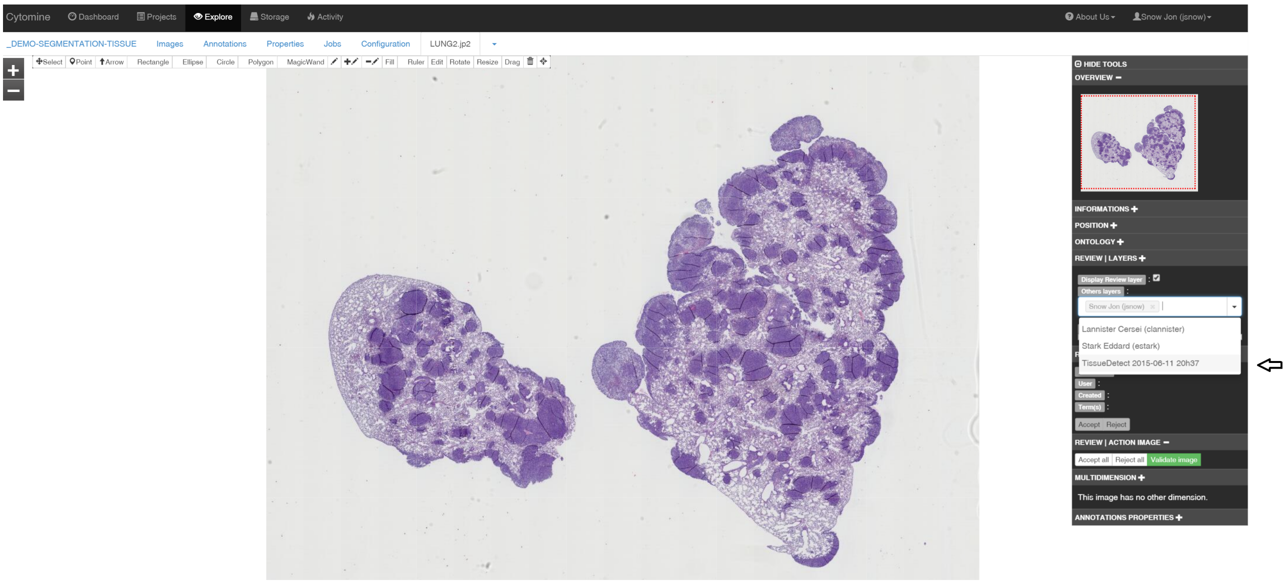

It displays the unvalidated annotations with a lighter opacity and red borders:

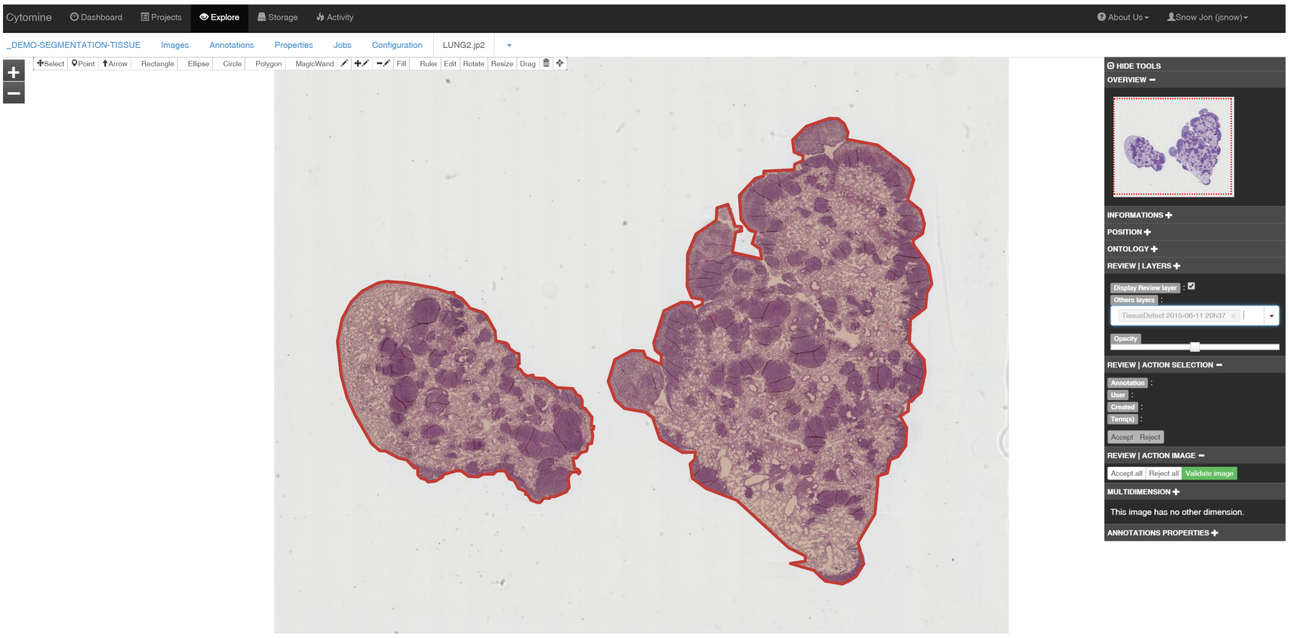

The user can then accept annotations one by one (by selecting the annotation, then click on "Accept" or press "A" on the keyboard) or all at one (by clicking on "Accept all"). Validated annotations are colored with the original color of the ontology Term and have green borders.
Concretely, these annotations are copied into the "Reviewed" annotations of that image:

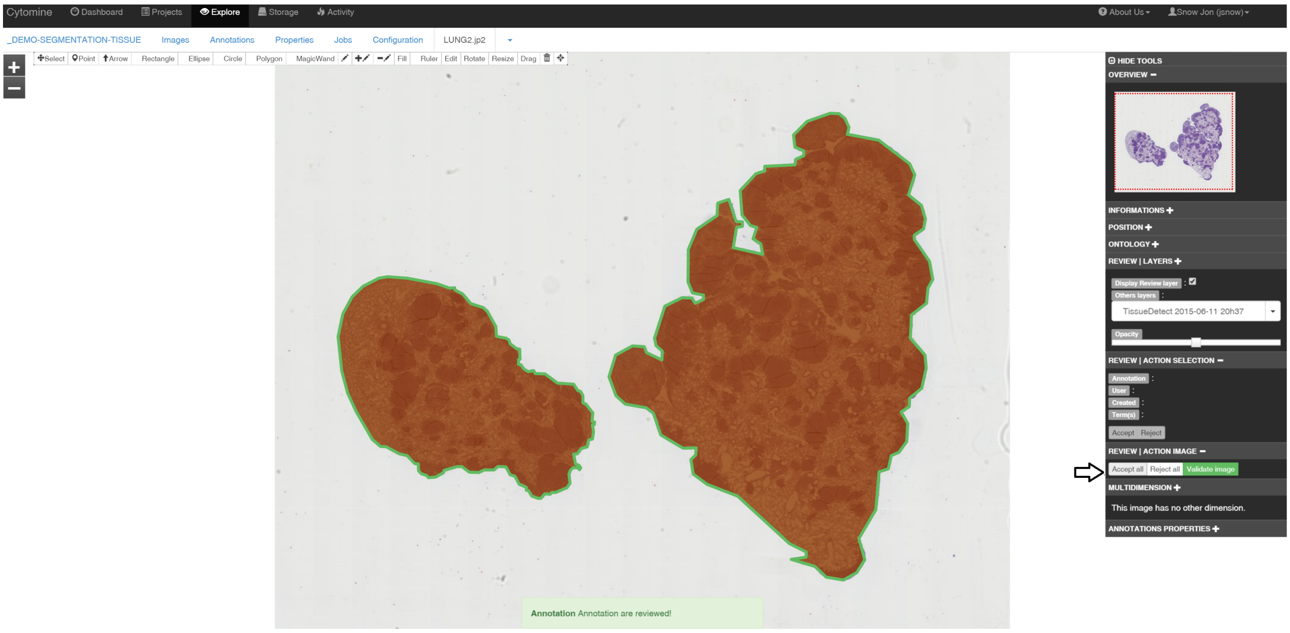

Once sections have been detected (e.g. to compute section Area), a typical application might be to detect tumor islets within them. For that, we use the Cytomine-DataMining Semantic Segmentation analysis module. We will exploit Tumor and Negative manual annotations to build a Tumor detection model, then we will use the model to segment whole slide images. As for the TissueDetect module, the software has to be added to the Project (if not already done) in the Project Configuration panel. Then the TissueSegmentModel_builder has to be run using appropriate parameter values (in our case select the current Project identifier to download annotations, the "Tumor" as predicted terms, the "Section" as excluded terms, and zoom level = 2). Learning is in general only performed once in a project (using annotations coming from this project or other ones), provided that images within a given project have similar visual appearances.

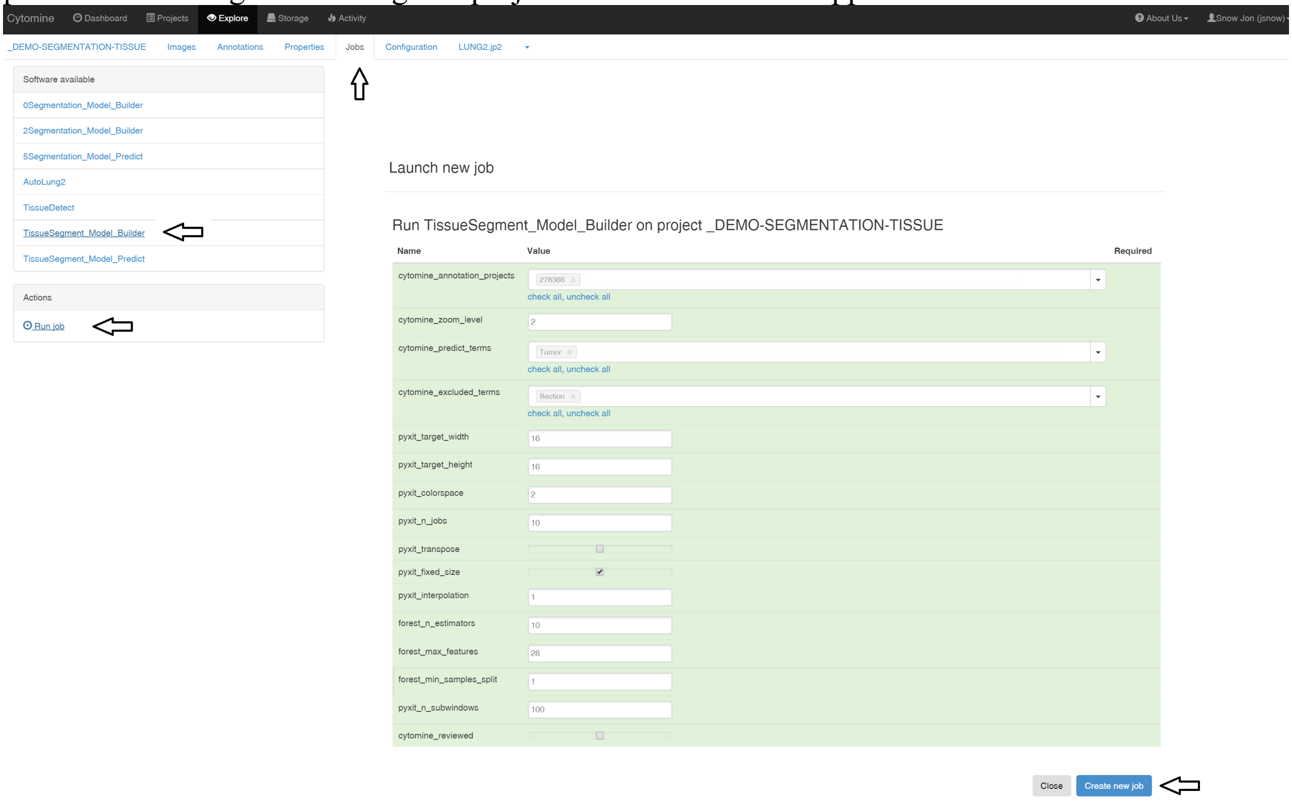

Once the Job reaches the "Success" status (in roughly one minute using our "toy" data on the demo instance), the Tumor detection model is ready to be applied to whole-slide images. The user then launch the TissueSegment_Prediction Job using appropriate parameter values (the image identifier, identifier of the TissueSegmentModel_builder job which created the model, the "Tumor" as predict term, the "Section" ROI term and activate the cytomine_reviewed_roi to only apply the model within validated Sections, zoom_level = 2):

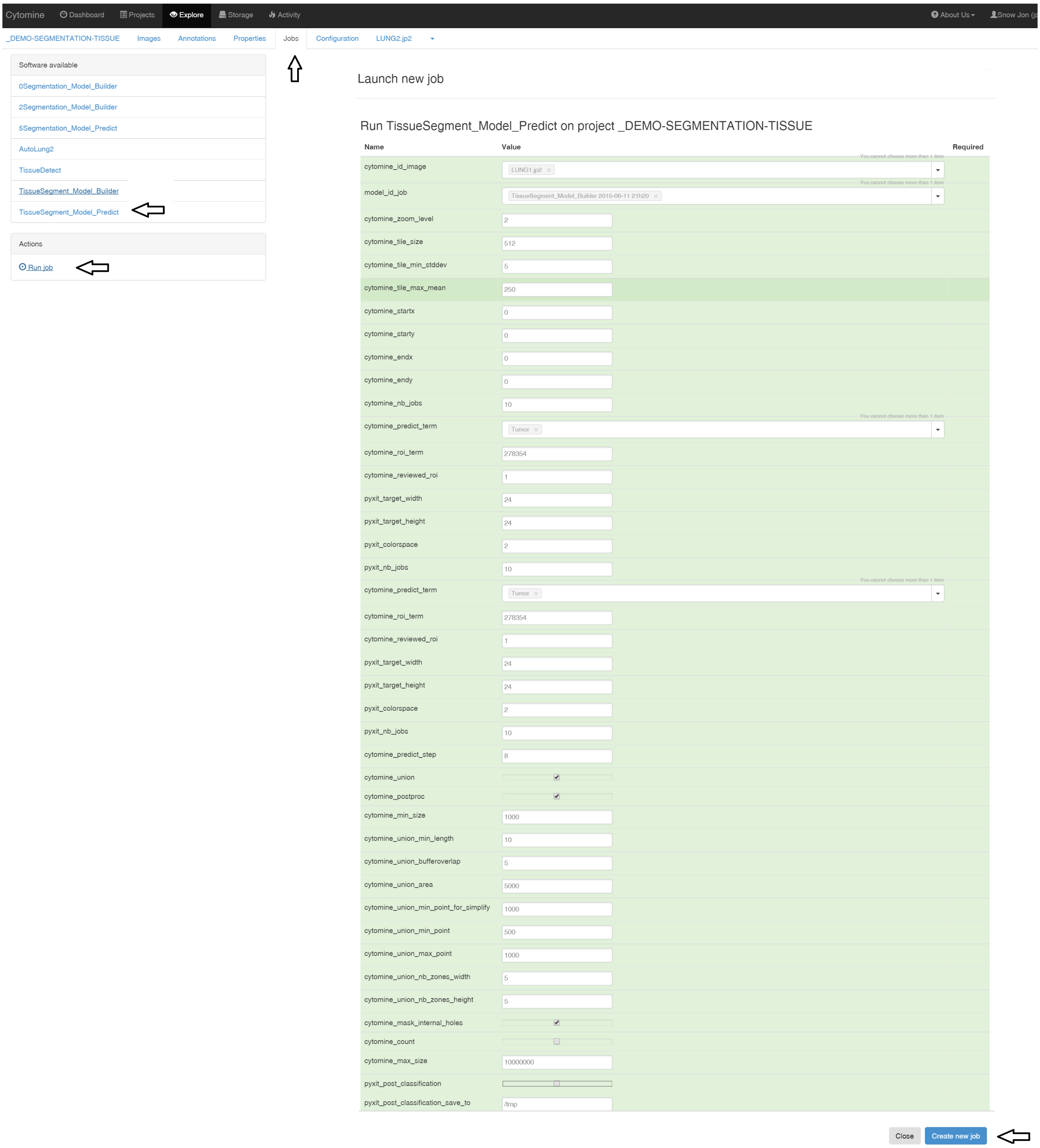

This procedure can take time (from minutes to hours) depending on model complexity, image sizes, resolution level, and allocated computing resources. It will create progressively annotations in tiles of the image. The process is running in the background and the user can perform other tasks meanwhile. The progress can be seen visually by opening the image and selecting in the "Annotation layer" tool the running Job, as illustrated below:

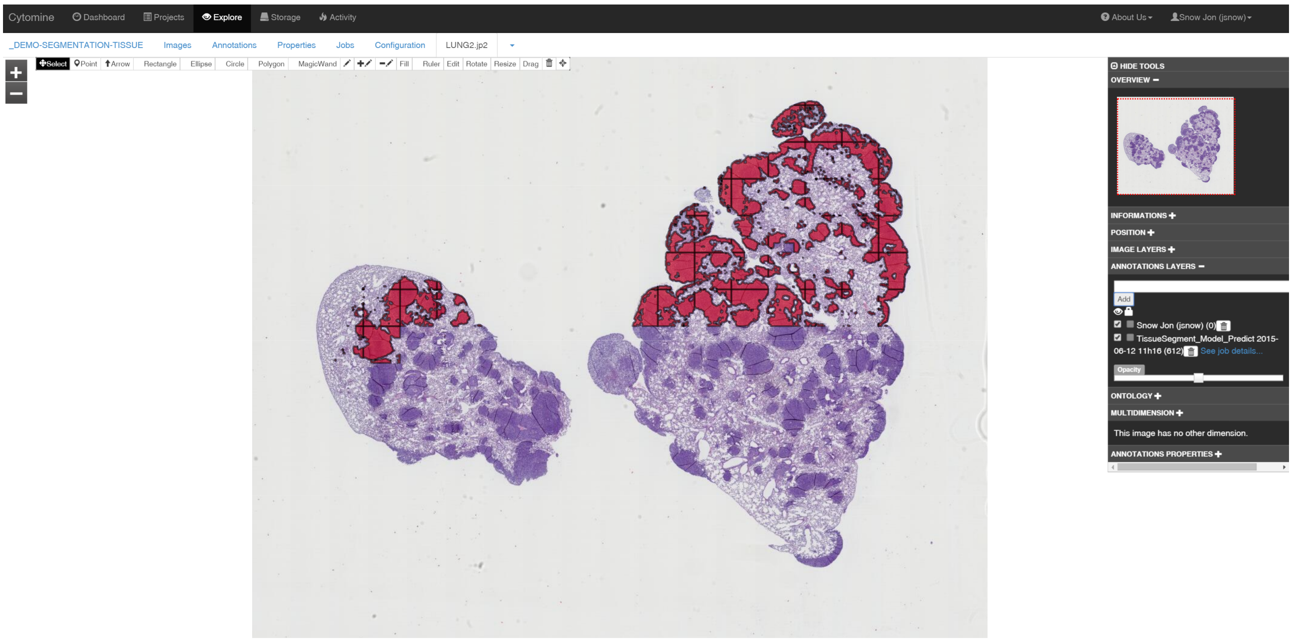

Then once all tiles are processed, the module will apply post-processing and an union procedure of all overlapping geometries. Once the whole process is done (it displays the "Success" status in the Job panel, it can take roughly 40 minutes to process this "toy" image on our demo instance), the user can review these tumor annotations using the same Reviewing procedure as for Tissue detection, by selecting the corresponding TissueSegment_Prediction job (the "Section" annotations can be hidden to ease reviewing by unselecting the box "Show" in the Ontology panel):

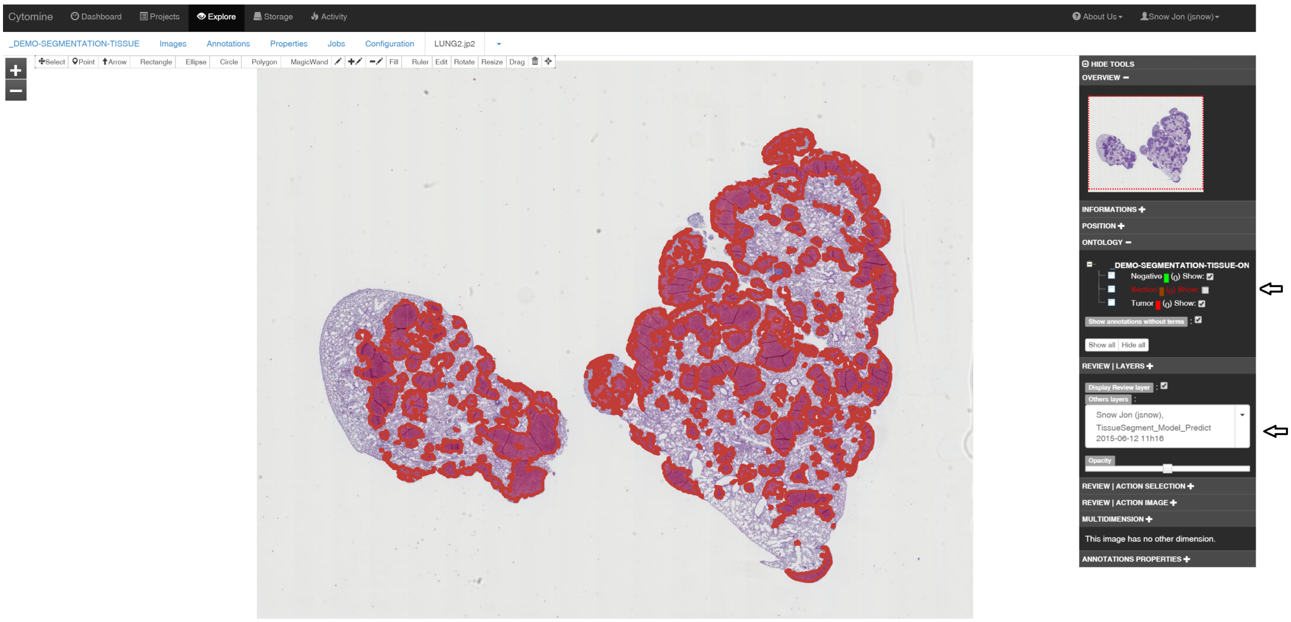

When most of the annotations are correct, we recommend to use the "Accept all" operations that will validate all annotations at once (they now take visually the original term color and have a green border) :

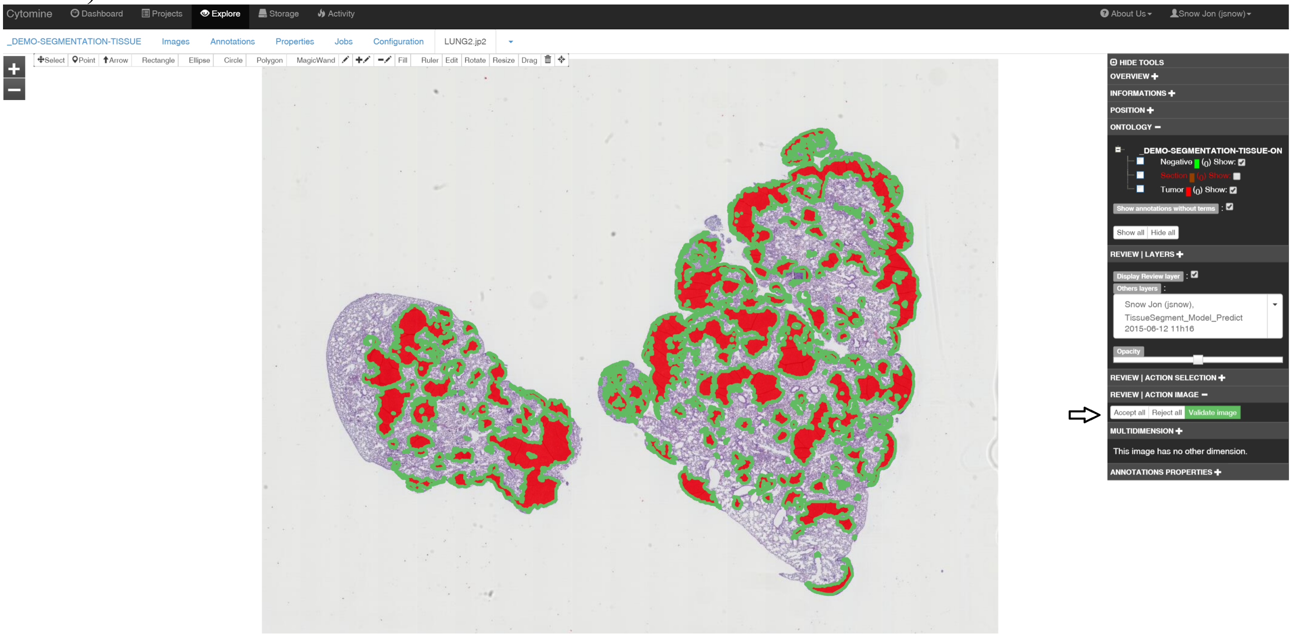

If annotations are not satisfactory at all, we recommend to annotate manually more examples (corresponding to observed predictions errors) and re-train a model. If only some annotations do not fit well the regions of interest (e.g. it misses portions of several tumor islets), the user can use proofreading tools to edit their geometries (or reject objects, e.g. tissue subtypes that are not tumor islets). Using the "t" keyboard or the "Display review layer" checkbox, the user can switch the display of the review layer for further inspection to look at the original tissue, and refine tumor contours using +/-/Fill polygon operations (+ operates an union of the draw polygon with the reviewed polygon, - subtract it):

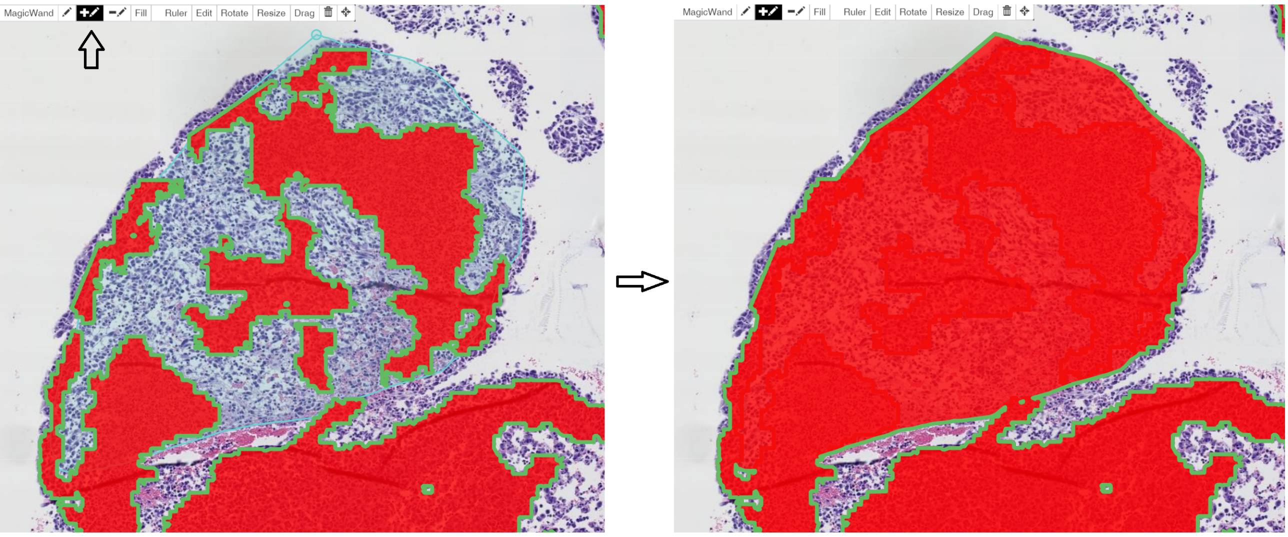

Once the whole image is proofread, the user has to Validate the image (green button) in the right

Tool panel:

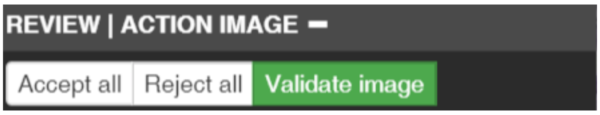

Now, it is possible to use the "ComputerTermArea" software to generate statistics about Section and Tumor area and ratios. Add the software to the project (once) in the Project configuration Panel, and run the Job:

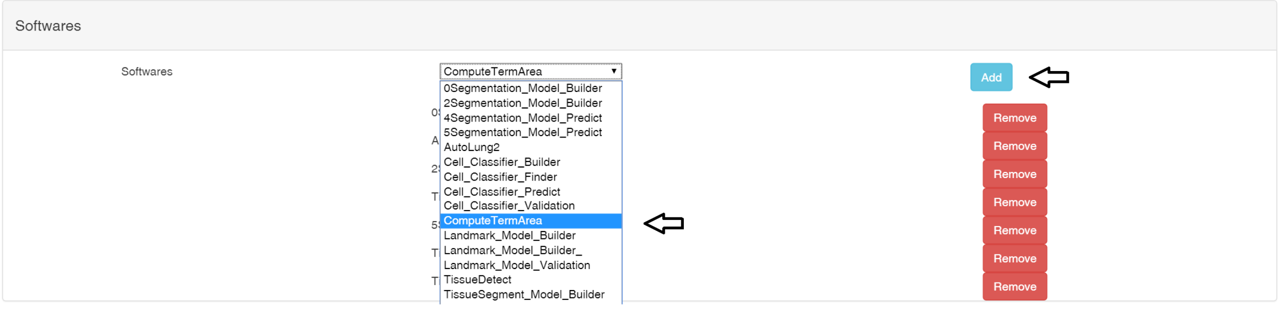

Then run the job, select "Tumor" and "Section" as Terms, and your image, in order to generate and download a CSV file with statistics:

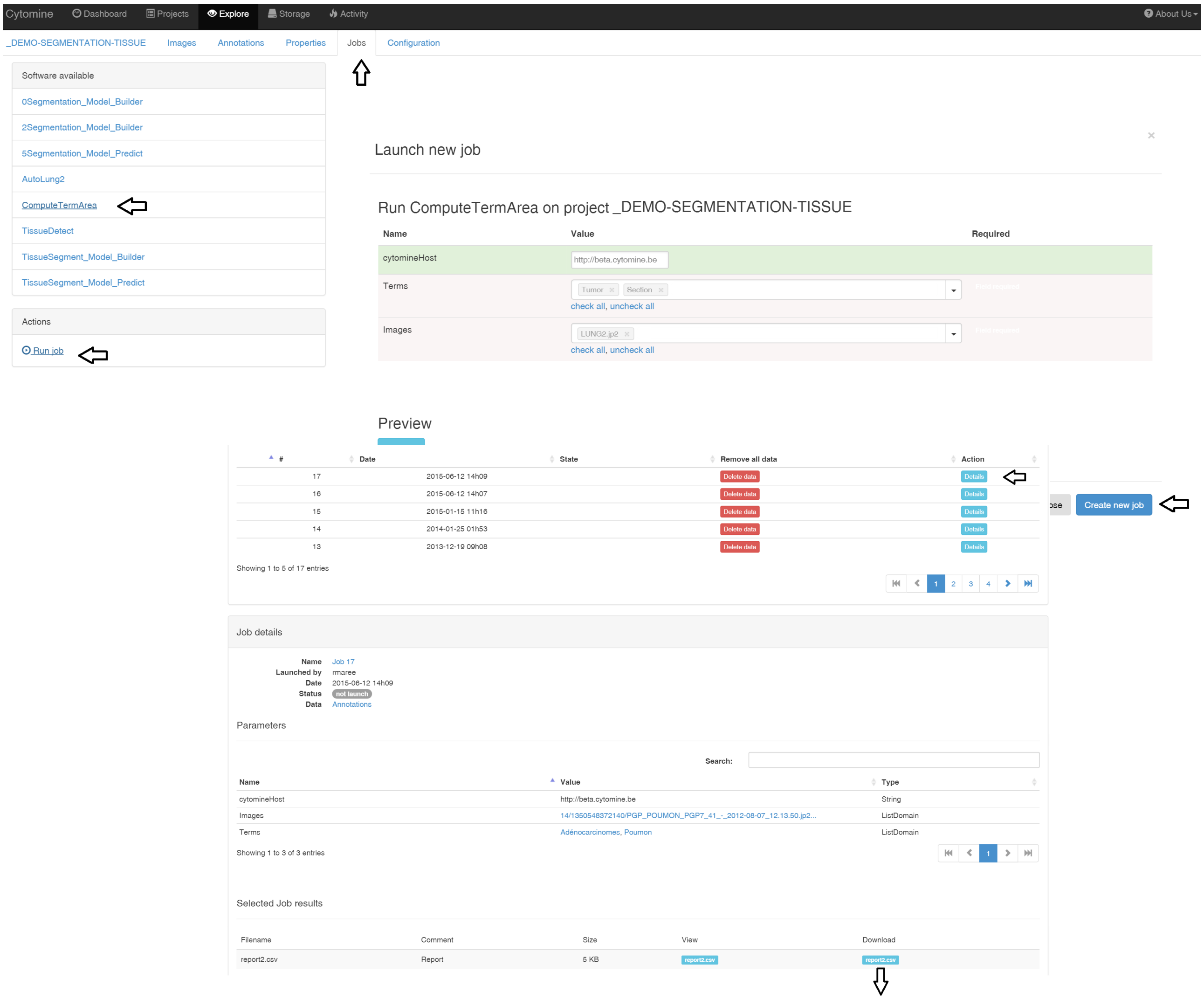

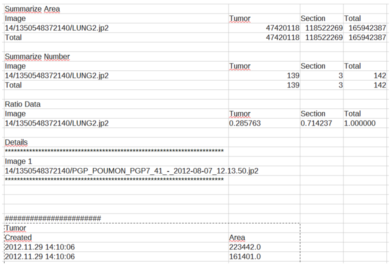

Summarize Area			
Image	Tumor	Section	Total
14/1350548372140/LUNG2.jp2	47420118	118522269	165942387
Total	47420118	118522269	165942387
Summarize Number			
Image	Tumor	Section	Total
14/1350548372140/LUNG2.jp2	139	3	142
Total	139	3	142
Ratio Data			
Image	Tumor	Section	Total
14/1350548372140/LUNG2.jp2	0.285763	0.714237	1.000000
Details			
**			
Image 1			
14/1350548372140/PGP_POUMON_PGP7_41_-_2012-08-07_12.13.50.jp2			
**			
########################			
Tumor			
Created	Area		
2012.11.29 14:10:06	223442.0		
2012.11.29 14:10:06	161401.0		

5.2.4.2 Object detection and classification

In this guide, we explain how to apply an object finder procedure followed by an object classification step, e.g. to classify positive and negative cells in cytology images. This example uses four softwares from the Cytomine-DataMining analysis modules: classification validation, classification model builder, object finder, and classification prediction. The provided "toy" demo project (DEMO-CLASSIFICATION-CELL) contains two main classes of cells (10 positive in red, 10 negative in blue). The goal is to build a workflow to detect and classify these cells automatically.

First, the classification validation module allows to evaluate by cross-validation classification performances of the image classification algorithm, given its parameter values and manual annotations with semantic terms. It is launched as other modules through the Jobs panel of the Project:

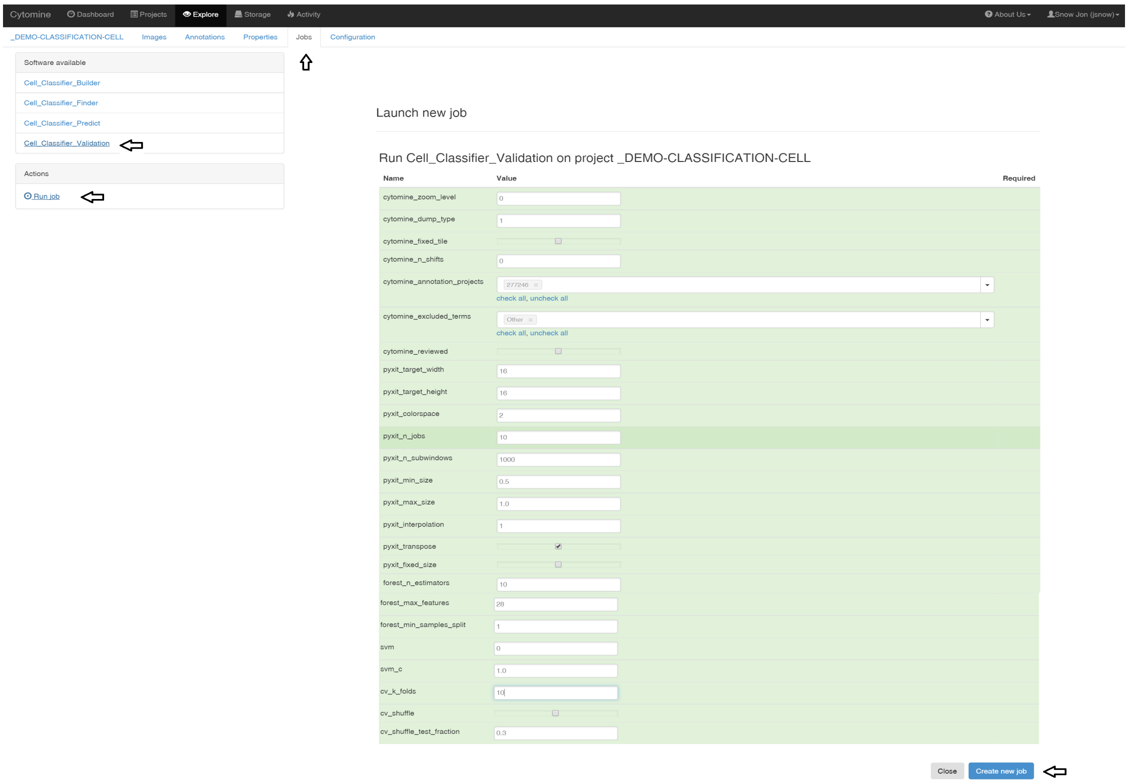

Once the process reaches the "Success" state, it is possible to view recognition rates, misclassified objects, and an interactive confusion matrix of the classifier in the Job Details (click on the Blue "Details" button, then scroll down and click on the blue "View confusion matrix"). It is possible to click on the confusion matrix numbers to view galleries of cell classifications. In our example, the classifier reaches 100% recognition rate for both cell types:

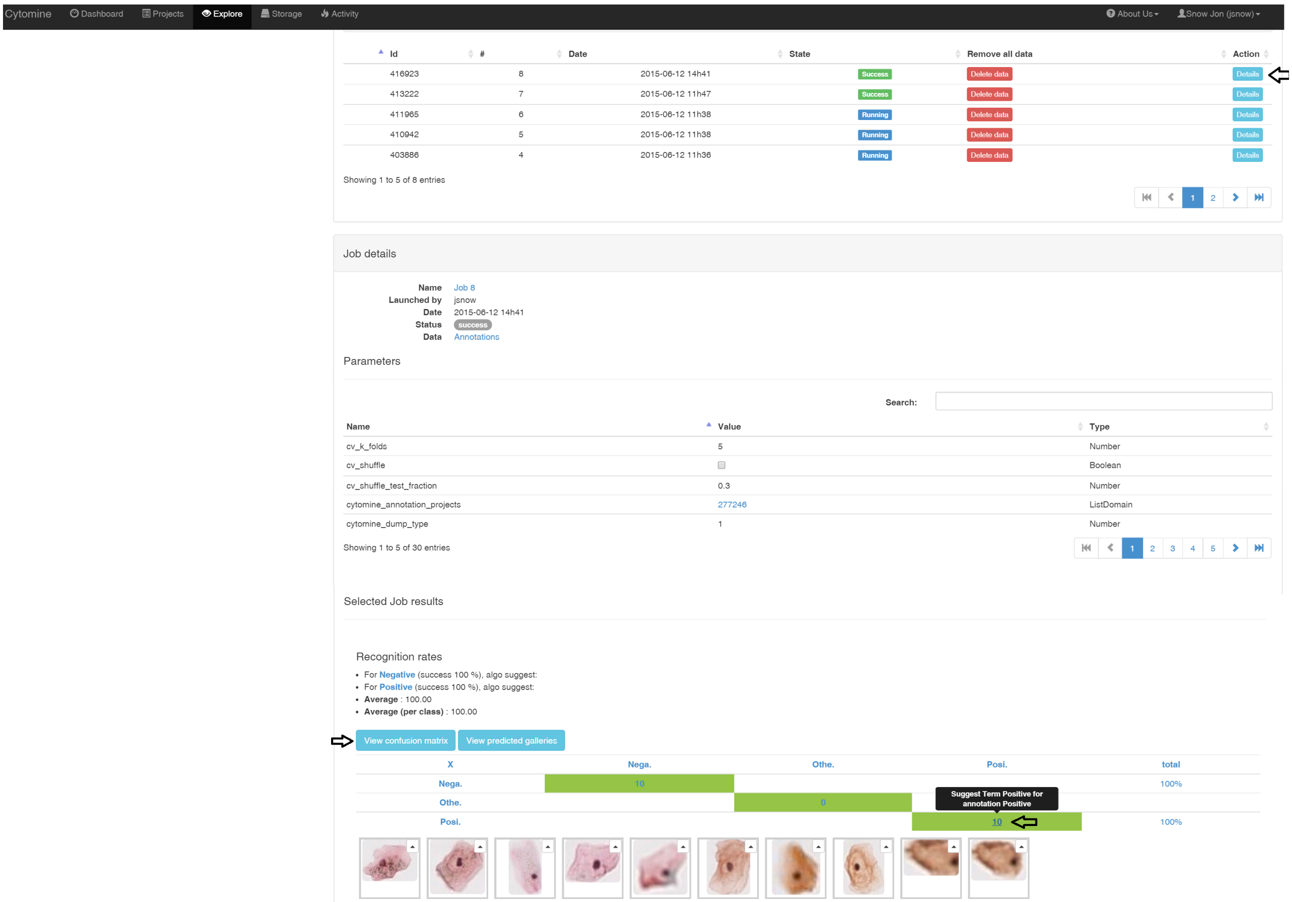

The classification validation module only evaluates classification models without saving them. Once satisfactory results are obtained, the user can build with corresponding parameter values and save a classification model by using the classification model builder module. It will save a model on the processing server that can be later reused by the classification prediction module (it takes less than one minute on our demo instance using our small "toy" data):

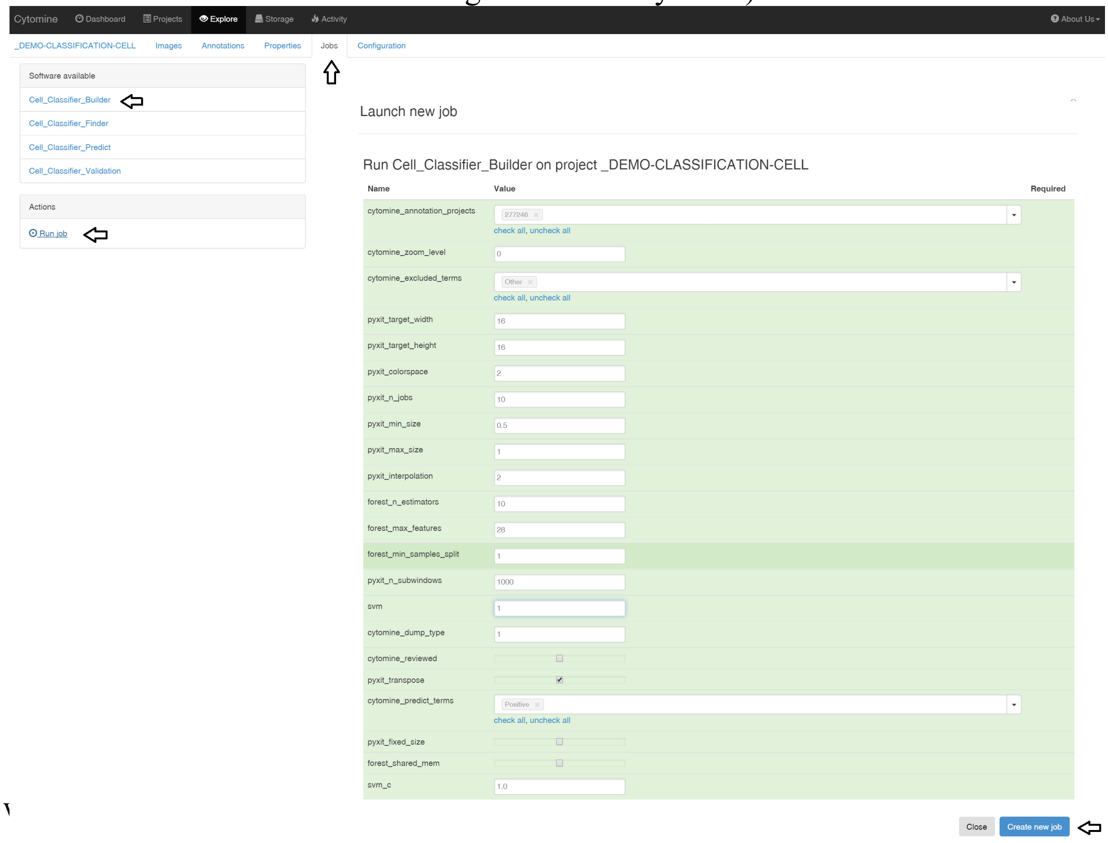

Now it is possible to apply this model to classify annotations of an image. We thus need before to apply an object finder to the image to create annotations corresponding to candidate objects. To ease the choice of a thresholding algorithm, it is first possible to apply standard algorithms on-the-fly, tile per tile, in the Cytomine-WebUI image explore view. The user needs first to add the image layer (in the Project Configuration panel), then apply it using the "Image Layers" panel at the right of the image Explore view:

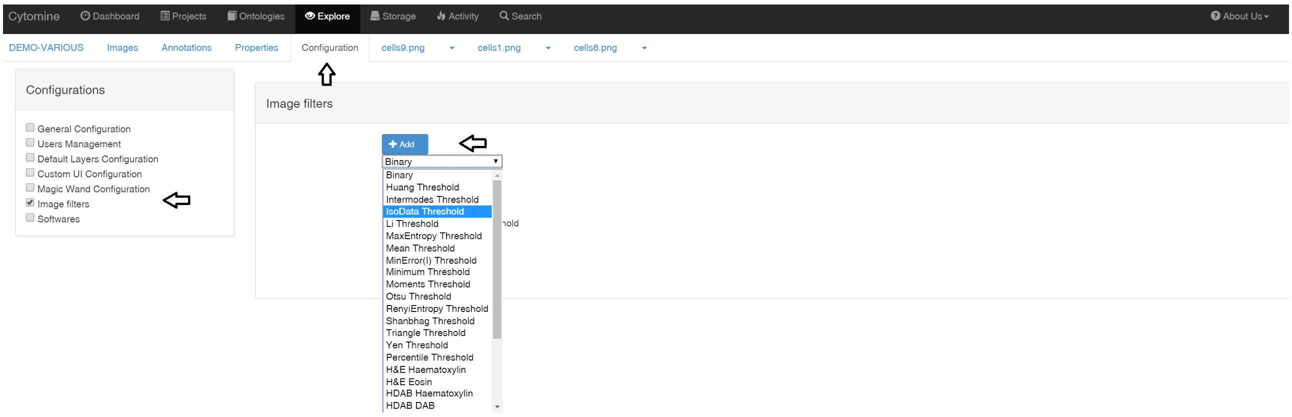

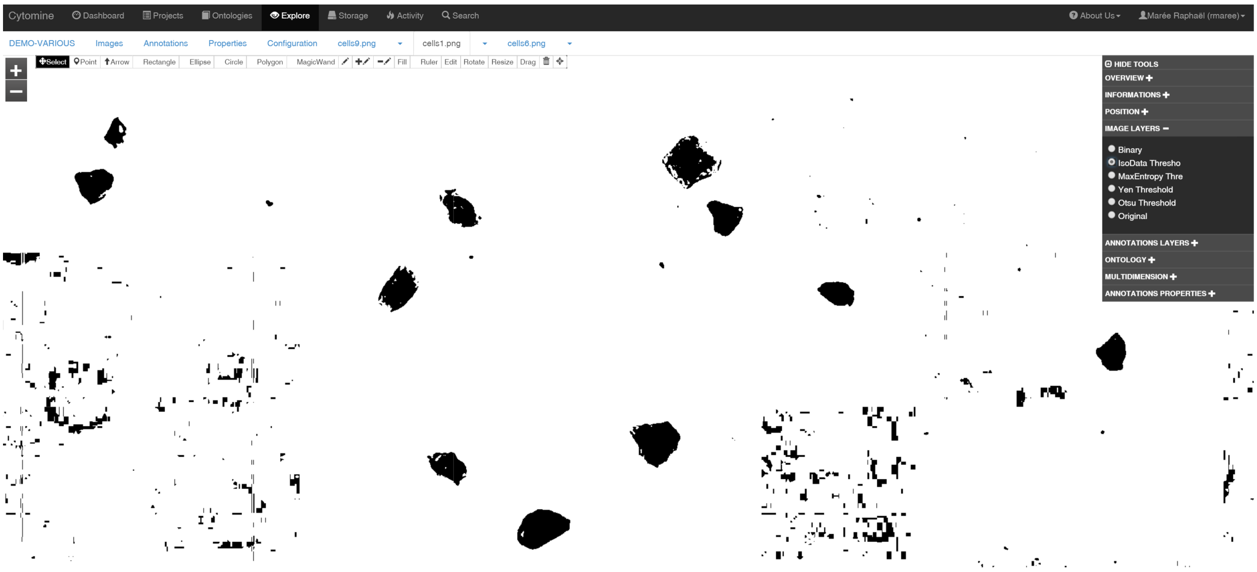

To apply the Object finder module on an image to create annotations, the user needs to add the software to the project (once), then launch it. It will create annotation objects in the corresponding job layer (here we use zoom level=1 and adaptive thresholding algorithm on cells1 image):

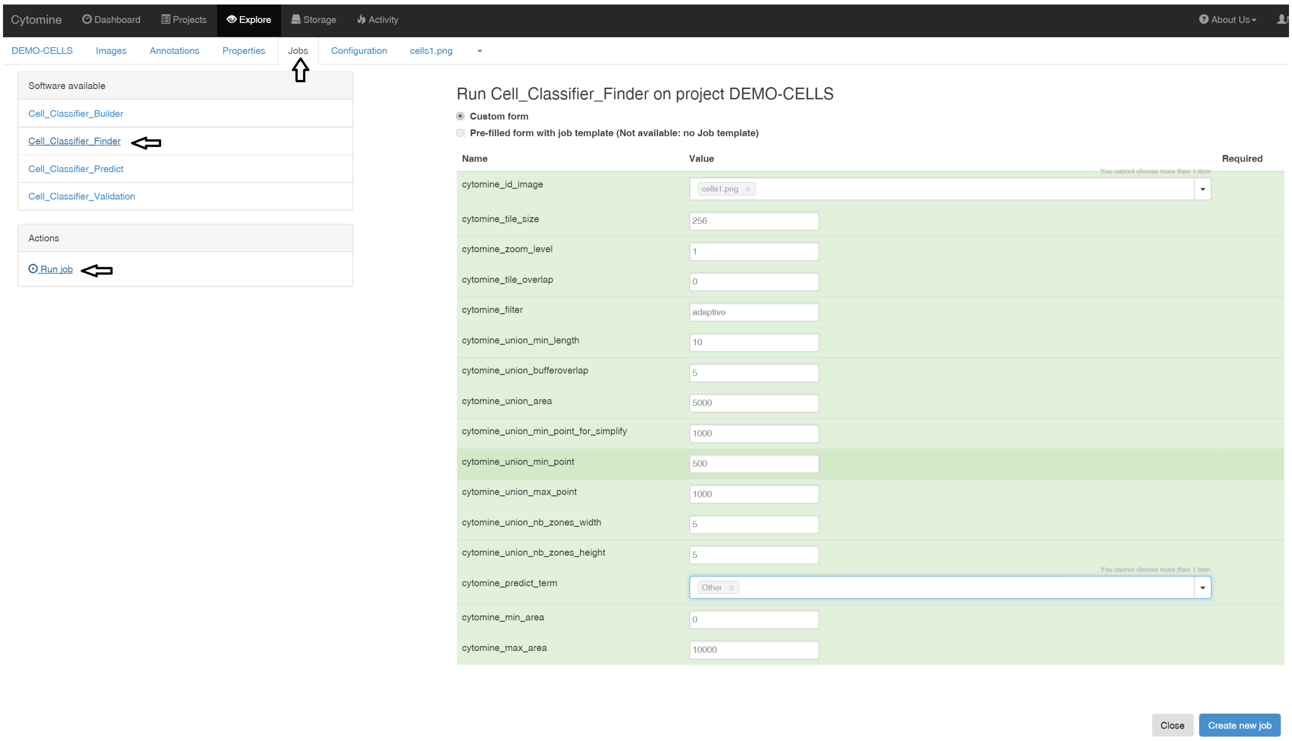

Once the job has reached its "Success" (less than one minute for one image on our demo instance) status, detected objects can be seen in "View predicted galleries" (blue button in the "Details" of the job) or directly in the Explore view of that image:

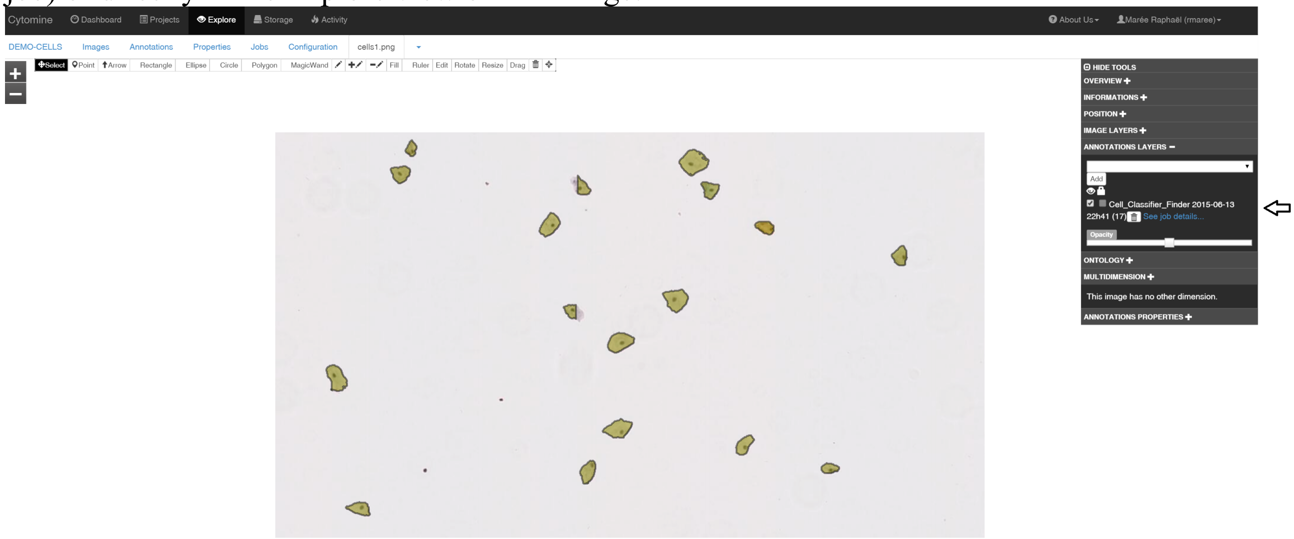

Now, we can apply a classifier to these detected objects in the image, by launching the classification prediction module (Cell_Classifier_Model_Predict), using the previously built classifier model, and the previously found candidate annotations (using the identifier of the job which generated these objects):

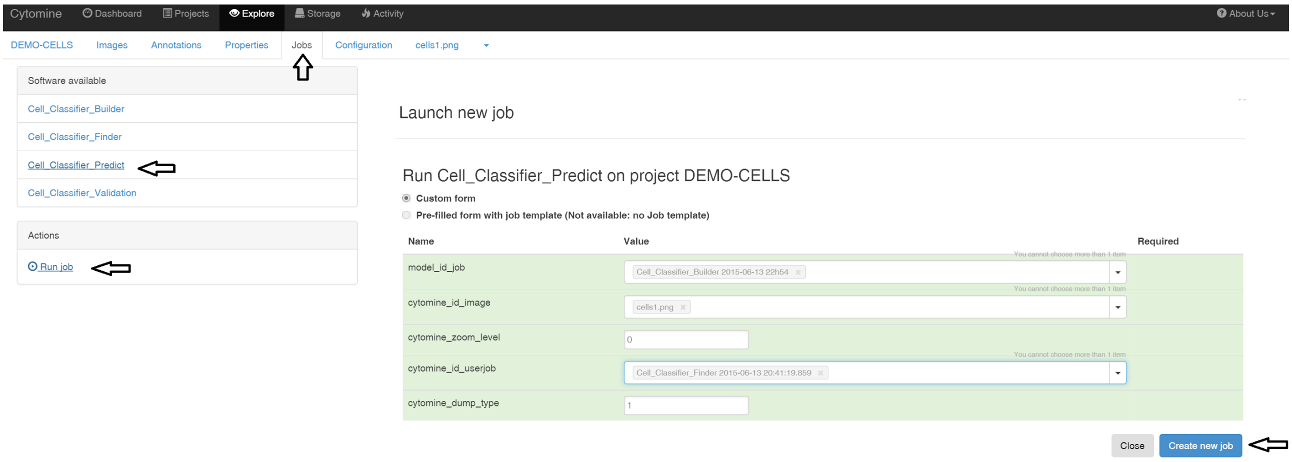

Once the classifier has reach its "Success" status (it takes less than one minute on our demo instance using annotations detected in cells1.png image), it is possible to visualize its classifications (as another job layer, either in the Explore view, or as "Predicted galleries" in the Annotation tab through the Details of the job).

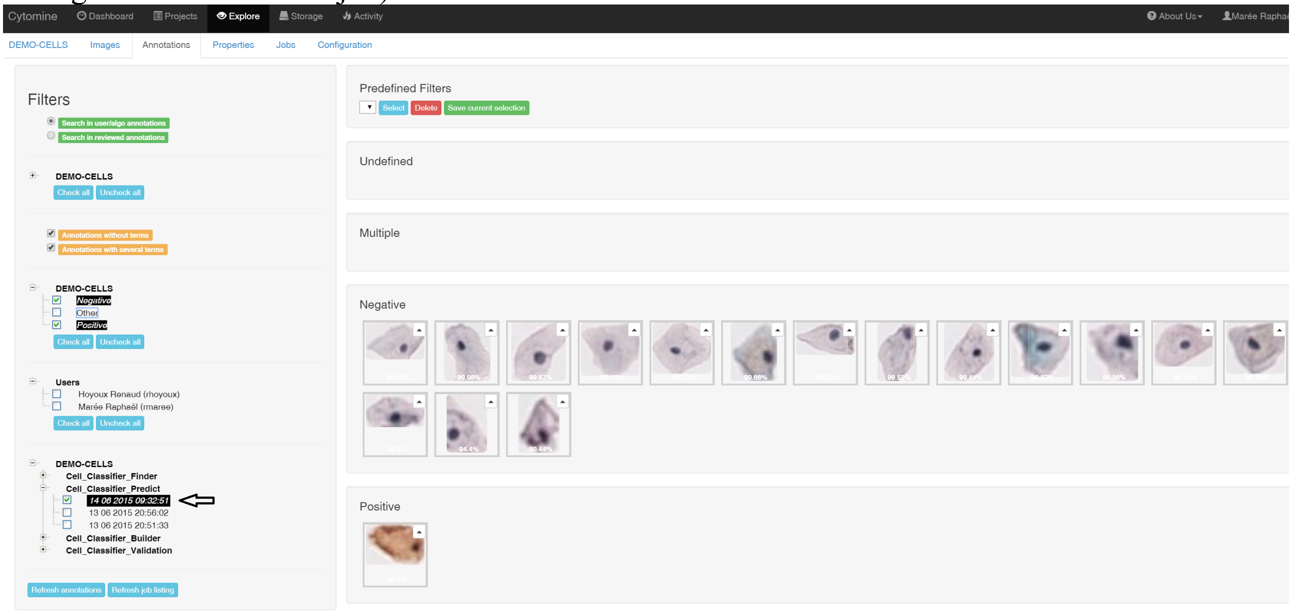

In this toy example, the classifier perfectly classifies cells (one positive in green, others negative).

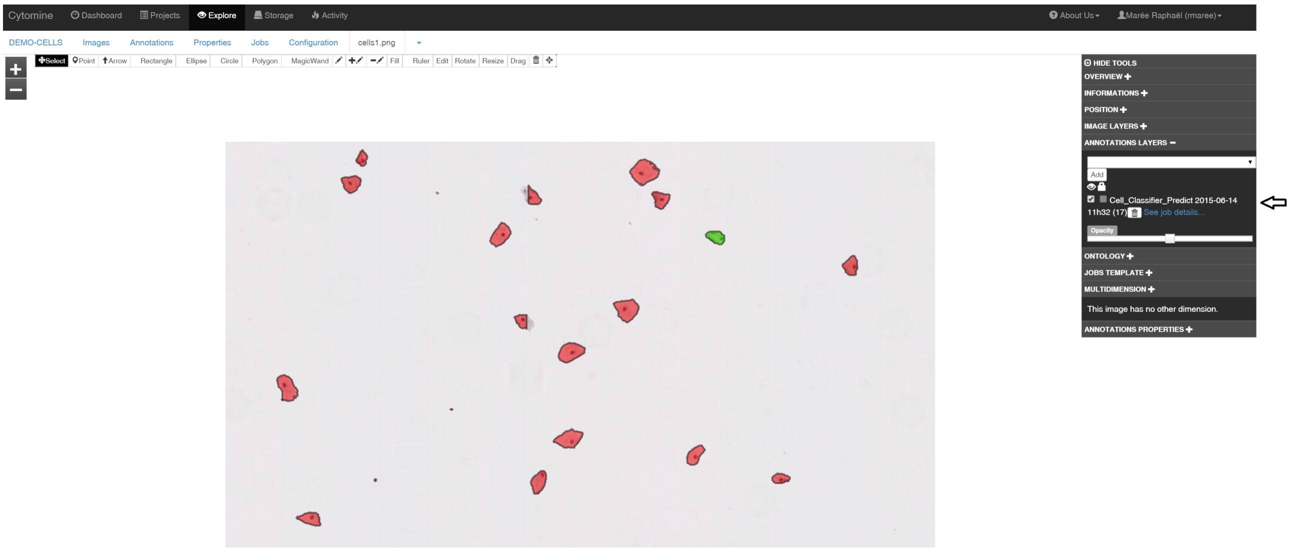

As in the tissue detection application, these annotations can be reviewed by an expert. We have developed a specific "Review (Objects)" module to review object classifications in a more efficient way. It can be launched from the Project image listing:

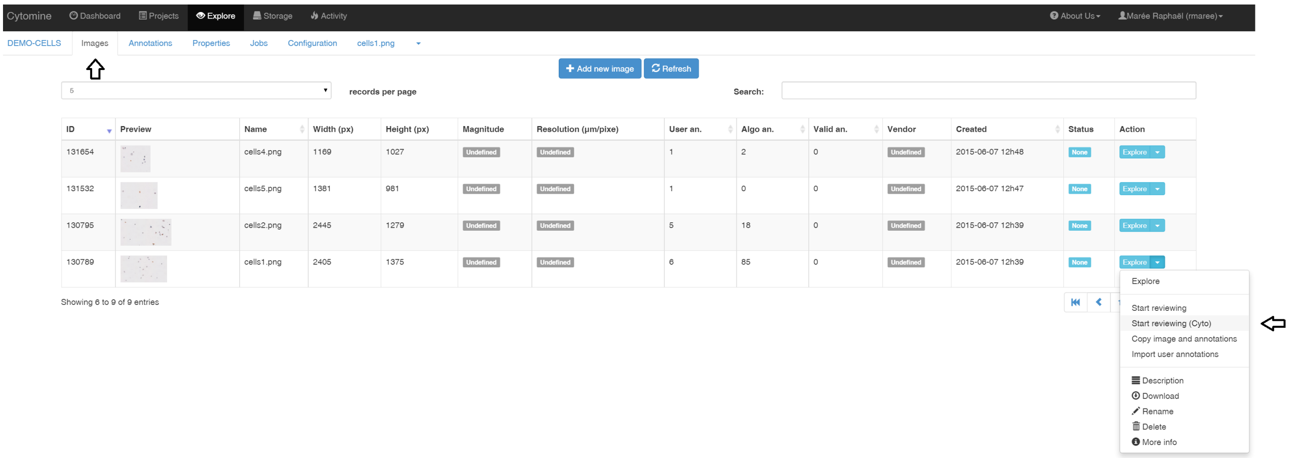

This module allows to select a user/job layer and displays galleries of its predictions (using pagination across all annotations). The user can then either accept these classifications, or correct them by drag'n'drop into visual boxes corresponding to the correct term. Validated and corrected annotations are copied to the Review layer of that image.

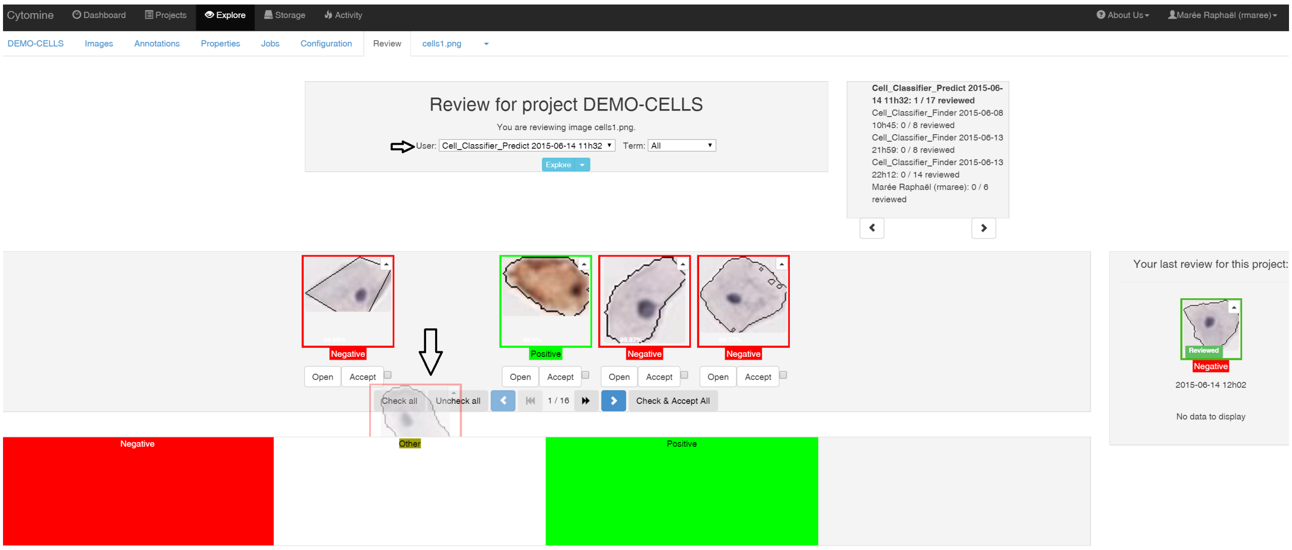

5.2.4.3 Landmark detection

In this guide, we explain how to use the landmark detection module on a "toy" dataset of Zebrafish embryo images. This example uses two softwares from the Cytomine-DataMining analysis modules: Landmark_Builder and Landmark_Predict. The provided "toy" demo project (DEMO-LANDMARK-ZEBRAFISH) contains five landmarks for 20 images, and 10 unlabelled images. The goal is to build a workflow to detect these landmarks automatically in the unlabelled images.

This module uses examples of landmarks positioned in images to build landmark recognition models. Here is an example of 5 manual landmarks on Zebrafish alizarin red images:

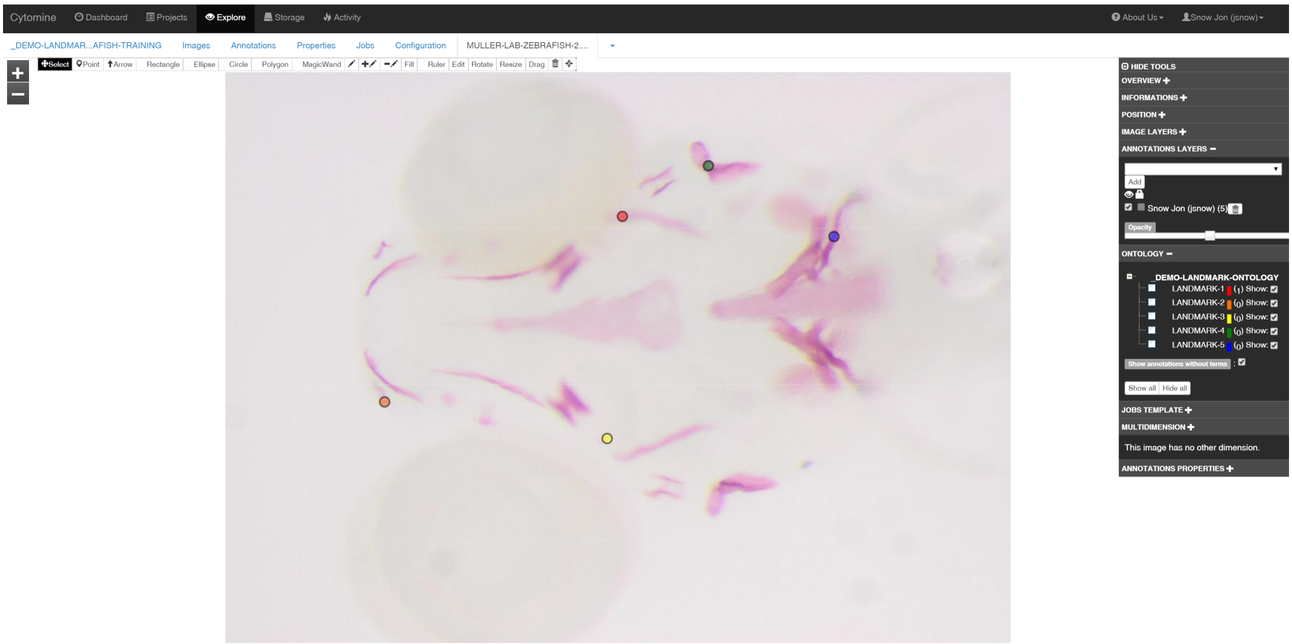

As previously, to launch the learning procedure, the user first needs to add the software Landmark_Model_Builder to its project (if not alreay done) in the Project Configuration panel:

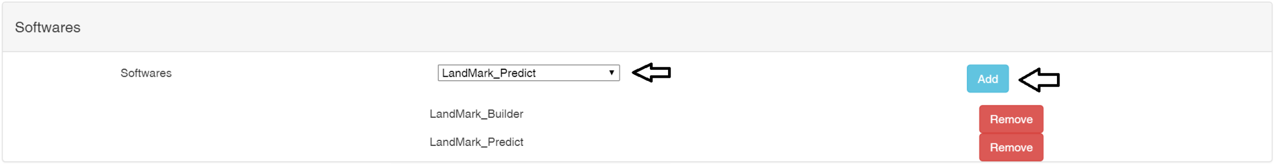

Then, it launches the training algorithm (the algorithm will use all landmark UserAnnotations from images of this project):

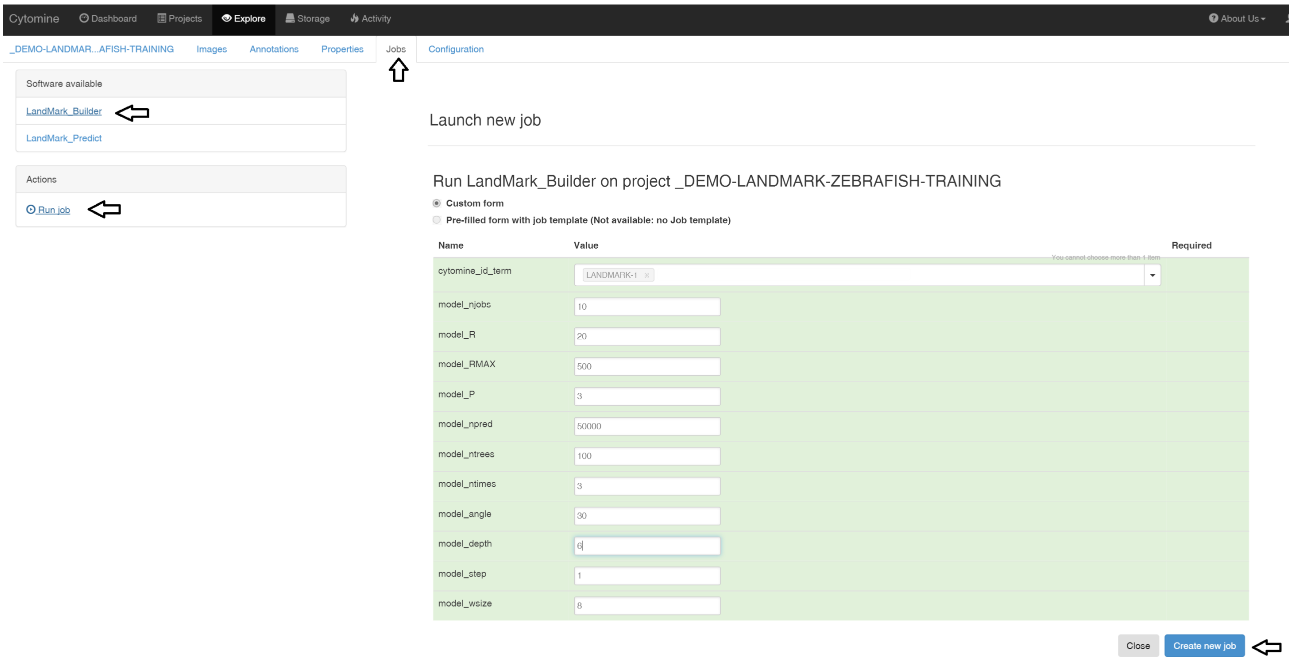

This procedure will generate a model to detect a single landmark. If multiple landmarks have to be detected, the training procedure should be repeated for other landmarks. Once a job has reached the "Success" status (it takes roughly 5 minutes per landmark on our demo instance with default parameter values), its model can be used to predict landmarks in other images. In this guide, we created models for landmark_1 to landmark_5 using 20 manually annotated images.
Applying models to predict landmarks is done by launching the Landmark_Model_Predict module and by selecting the previously build models:

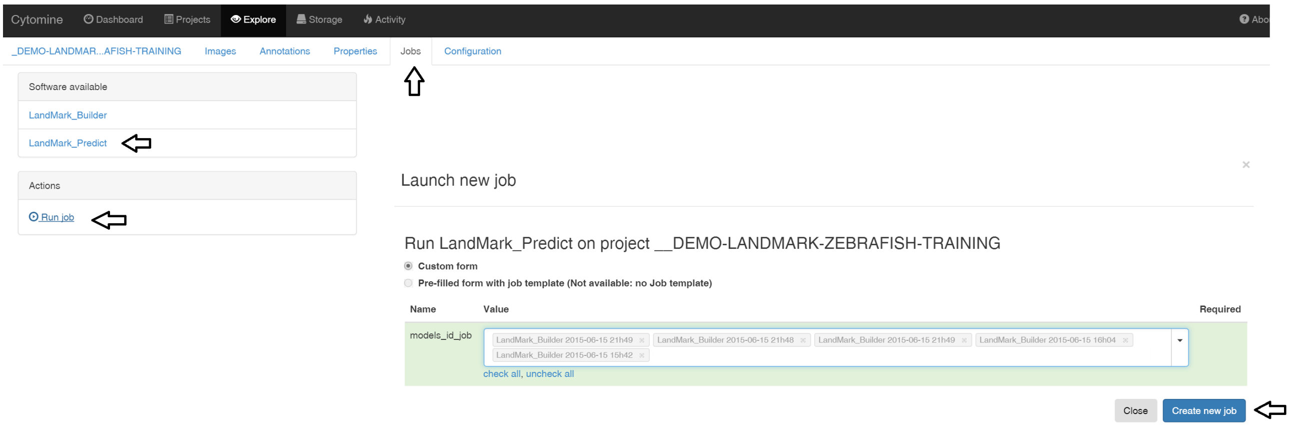

In our case, it will create Point annotations corresponding to the five predicted landmark positions in all 30 project images (it takes roughly 12 minutes on our demo instance with default parameter

values). Here we illustrate predictions for a training image (the Figure shows both manual and predicted landmarks to assess position precision, one can observe slight shifts only) and an unlablled image for which no manual annotation was provided for training the model:

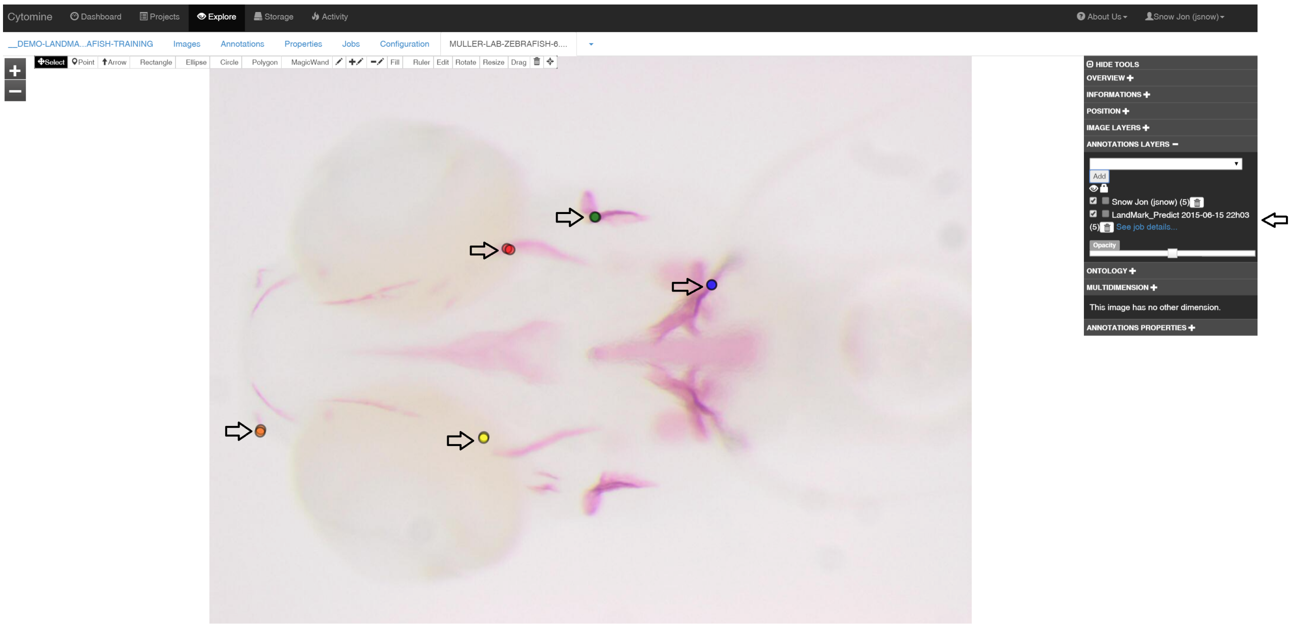

As with other modules, the user can now proofread these detections on new images. From the listing of images of the project, the user selects the "Review" function:

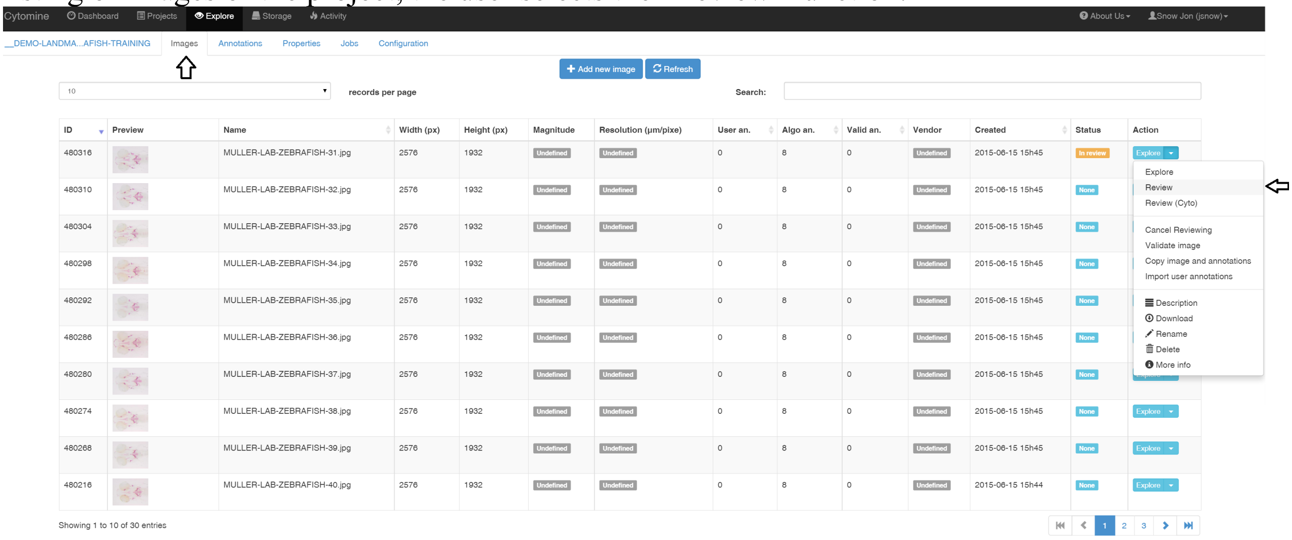

It opens the image in the Explore view with reviewing tools activated. The user first selects the UserJob layer in the right panel. When most of the landmarks are well detected, we recommend to use the "Accept all" function (Points now have a green border) and then use the "Drag" tool (click on the landmark to edit) to move landmarks that are not well detected only:

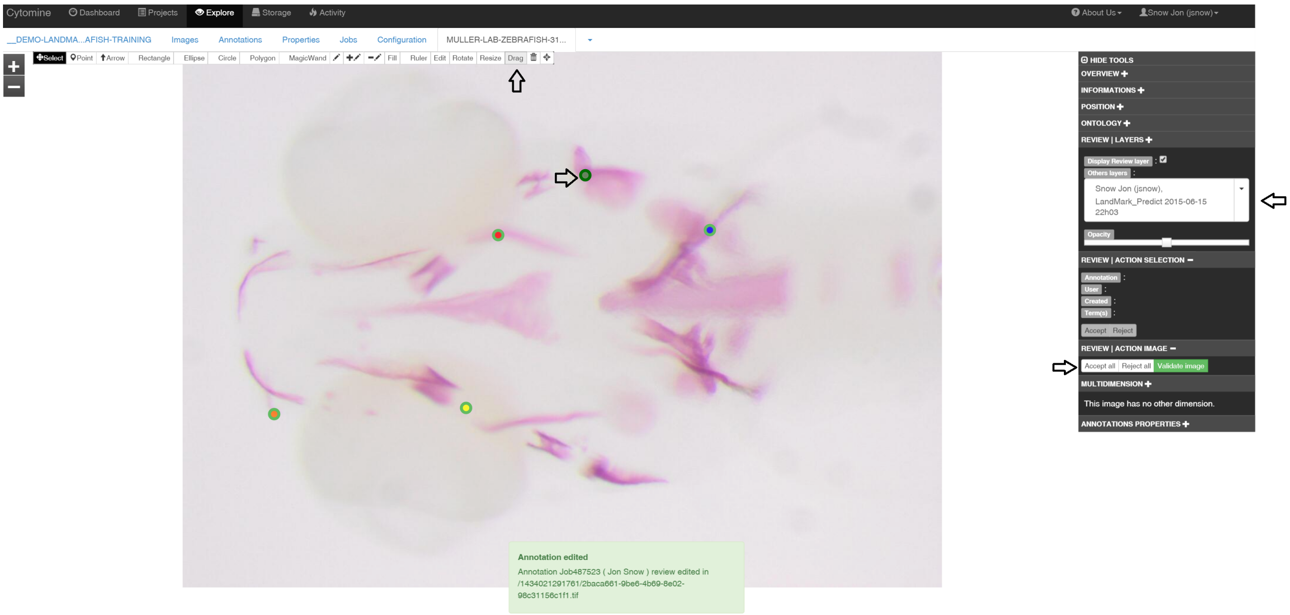

Once all landmarks are well positioned, the user validates the image (green button). Reviewed landmark positions can later be exported (using the Export Landmark job) to derive morphometric measurements.

5.2.5 Textual search

In addition to content-based image searching and user interface tools to filter annotation based on semantic terms, it is possible to perform textual searches through Cytomine-WebUI using http://demo.cytomine.be/#search-

Searching will be performed across data entities (projects, images, annotations, domains, properties, descriptions) that are accessible by the user. In the example below, the user looks for "Laboratory Tumor Development Biology" and the search engine displays a project that contains these words in its description:

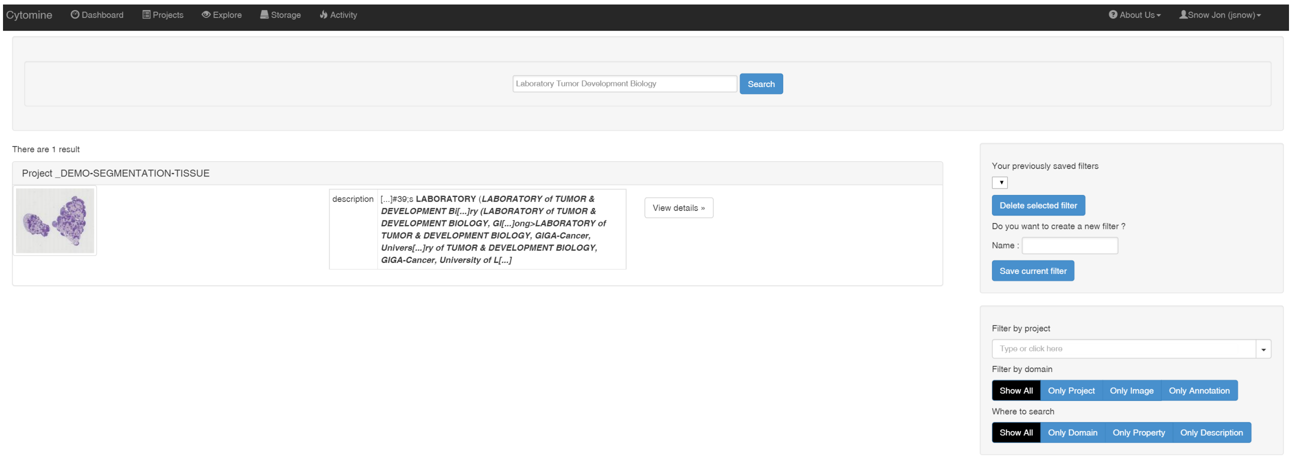

5.2.6 Online users

Cytomine can perform the tracking of all user activites (user positions in images are stored in the database) that e.g. allows multiple users to follow remotely another user observation paths and actions. Online users appear in the project dashboard, and in the Explore Annotation layers panel.

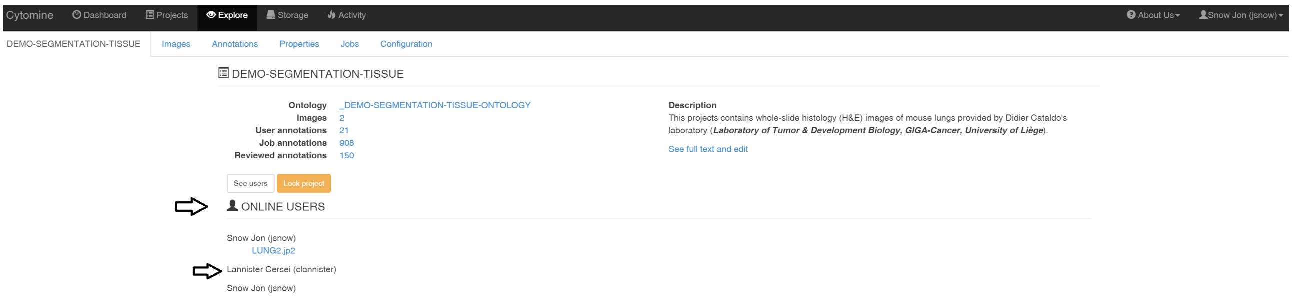

It is possible to follow another online user (in green) by checking the closest box next to the user name. Once the followed user moves in the gigapixel image or creates an annotation, the following user explore view will move to the same position and display novel annotations. Geographically distributed users can thus discuss remotely in front of the same image areas (using e.g. instant messaging or phone calls).

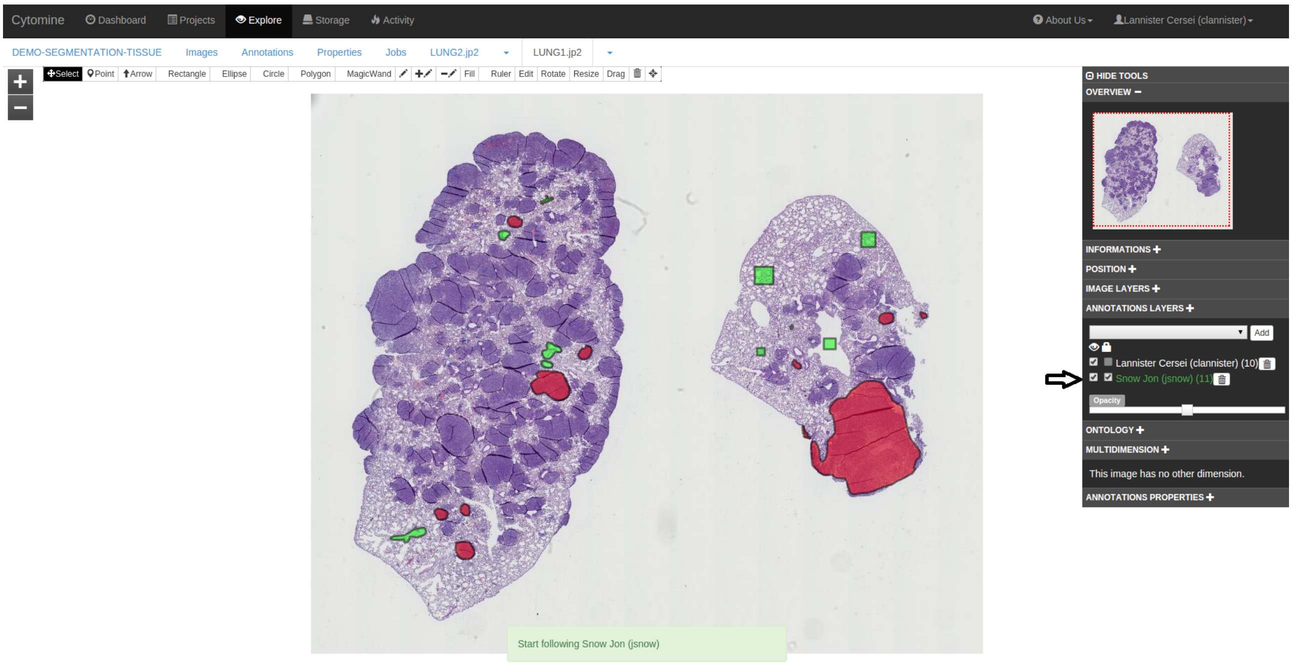

5.2.7 Blind assessment

5.2.7.1 Blind configuration

Cytomine has a blind mode that can be activated by administrators of a project in its Configuration Panel. This option will hide (for non-admin users) in Cytomine-WebUI image names (in the Image listing, the Explore view, …) so that the user quantifying experimental outcomes is blinded to the experimental setting that might appear in original filenames. This option also hides user activities and annotation statistics. This option might be used to reduce bias in analyzing imaging data.

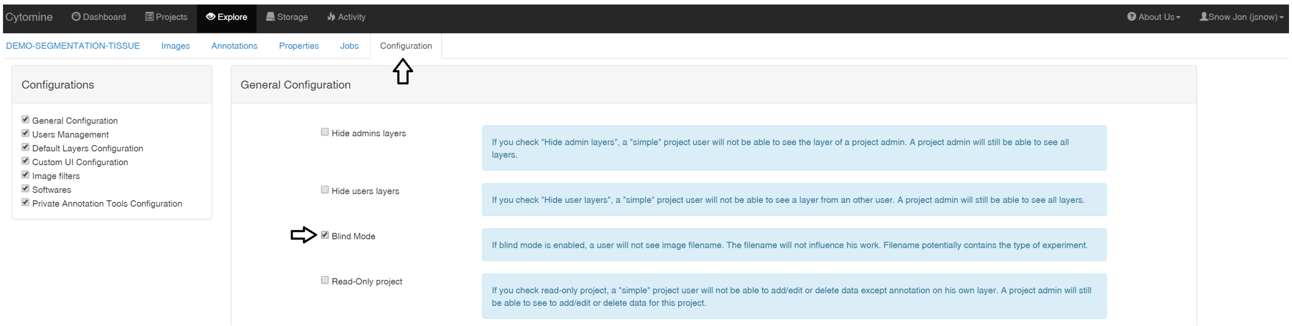

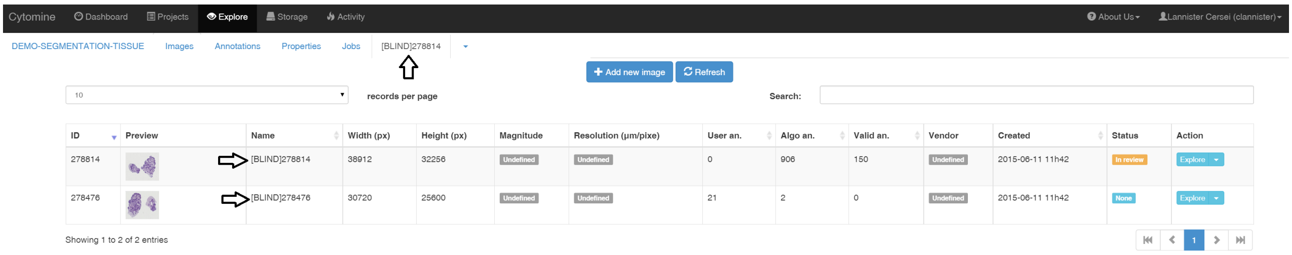

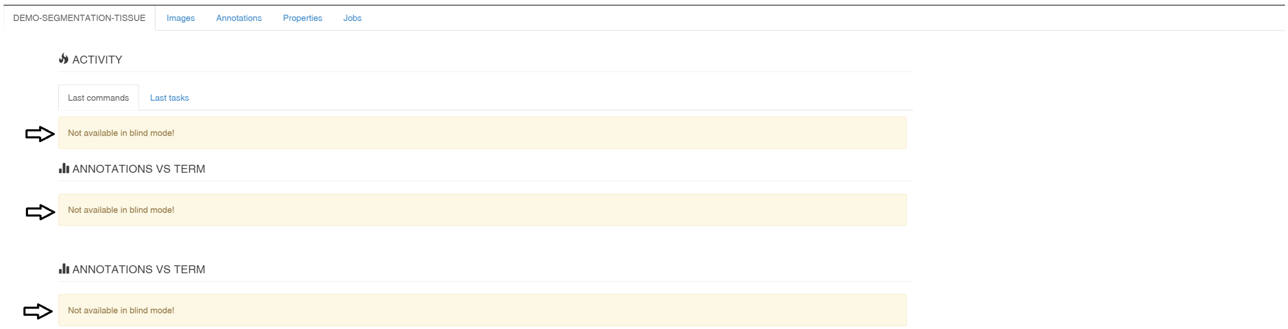

5.2.7.2 Cytomine-IRIS: Measuring Inter-observer Reliability

In contrast to the intentionally collaborative nature of the Cytomine-Core and Cytomine-WebUI annotation modules, Cytomine-IRIS, the inter-observer reliability study module, provides an intuitive web interface for blind assessment of annotations. It is based on the public Cytomine REST API described in section 6.3 to leverage existing Cytomine backend functionality like project and user management, or annotation services for manipulating ontology terms of annotations. On the other hand, it provides a completely decoupled user interface. In addition to the basic project configuration done using the regular Cytomine-WebUI, particular projects and images can be disabled per user on IRIS, such that for example within existing projects only few images are used for assessing the inter-observer reliability among particular users.

IRIS Installation

Since Cytomine-IRIS is an additional module, multiple instances of IRIS running on different servers can be connected to a single Cytomine-Core server. Thus, the installation of IRIS differs a bit and is detailed on https://github.com/cytomine/Cytomine-IRIS.

In contrast to previous sections, this user guide is based on larger dataset not yet publicly available and therefore it is not directly reproducible on our demo instance (http://demo-iris.cytomine.be/iris/index.html#/) but main functions are presented here to allow users to apply these concepts on a reduced "toy" dataset or on their own data.

Annotation Labeling and Reviewing

The structure of a standard Cytomine project is reflected in the workflow of IRIS: once a user logs into IRIS, a list of available projects is shown, where particular projects may be disabled just for this IRIS instance. In order to start labeling, the user opens a project and views a list of available images which contain the annotations:

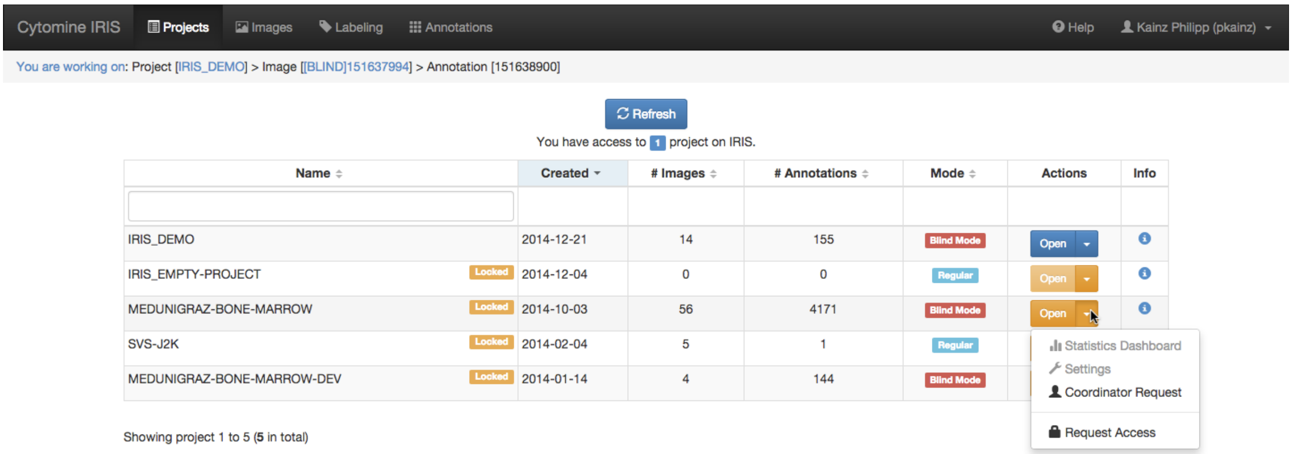

The image table also reflects the overall labeling progress within each image and provides functions for sorting and filtering. For example, this enables the user to filter out all images, where annotations are left to be labeled. This is particularly useful in situations where the study protocol requires all users to label all annotations, or when there are lots of images in a project.

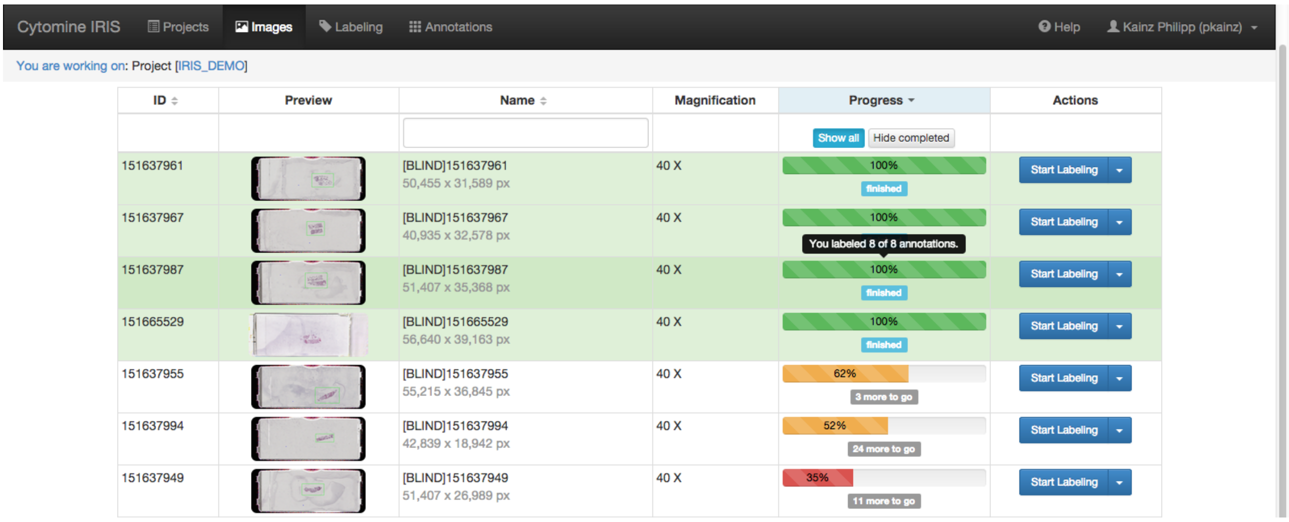

The annotation labeling can be performed per image in a dedicated view by selecting "Start Labeling" from the rightmost column. Image zooming and panning is available like in the main Cytomine-WebUI, allowing proper exploration of the image context of an annotation in order to

make an informed decision about the label. The user can hide annotations where a label already exists and thus just navigates through unlabeled annotations, while their order is preserved.
Navigation among annotations can be done either using the buttons above the image view or by pressing the keys “n” for the next, or “p” for the previous annotation. A label is assigned by simply selecting the term in the table on the right hand side, which is basically a flat representation of a possible hierarchical ontology:

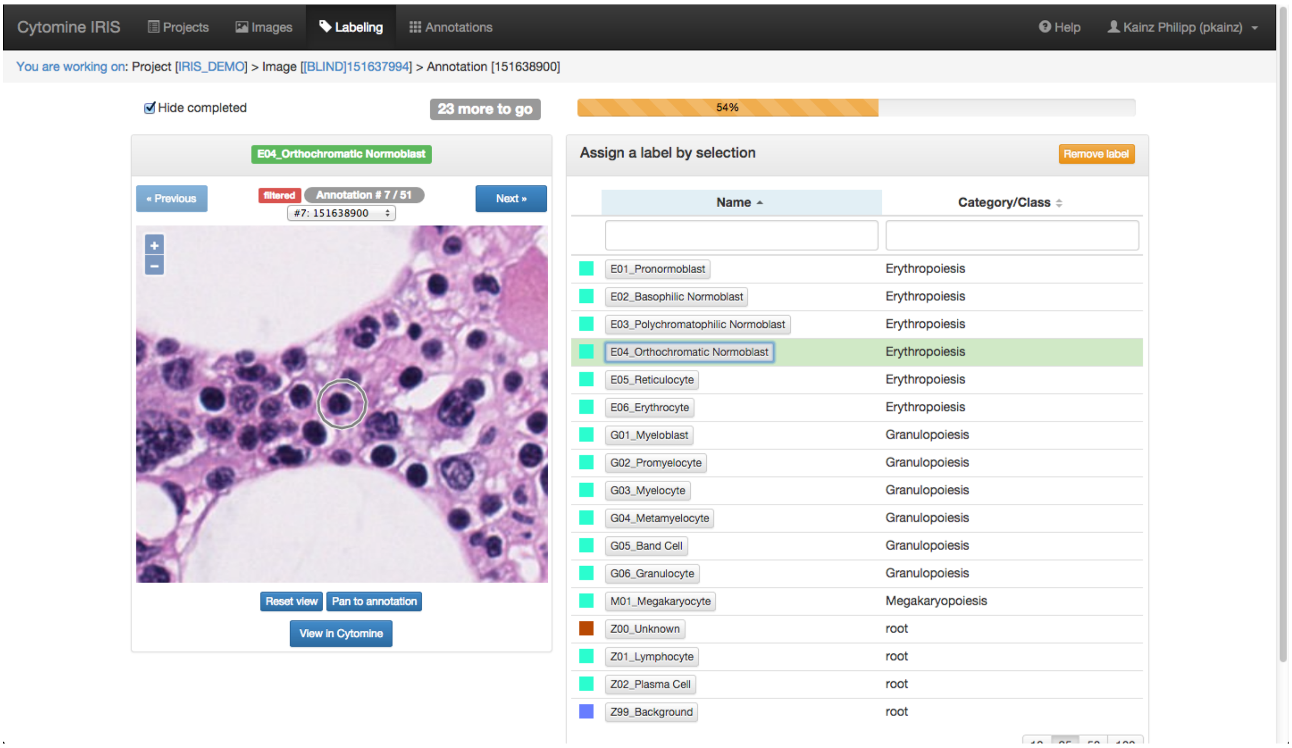

It is convenient to view annotations in a gallery-like view per ontology term, which can be done by selecting “Annotations” from the menu bar at the top. This enables users to visually filter out outliers that do not comply e.g. with the appearance of the majority of annotations having the same label. Moreover, the gallery can display annotations across different images in a project and provides several methods to correct the label either directly or by navigating to the labeling view, if more context around the annotation is required. Multiple objects can also be re-assigned labels at once, which drastically reduces the time of manually navigating to the corresponding images and finding these annotations in the labeling view.

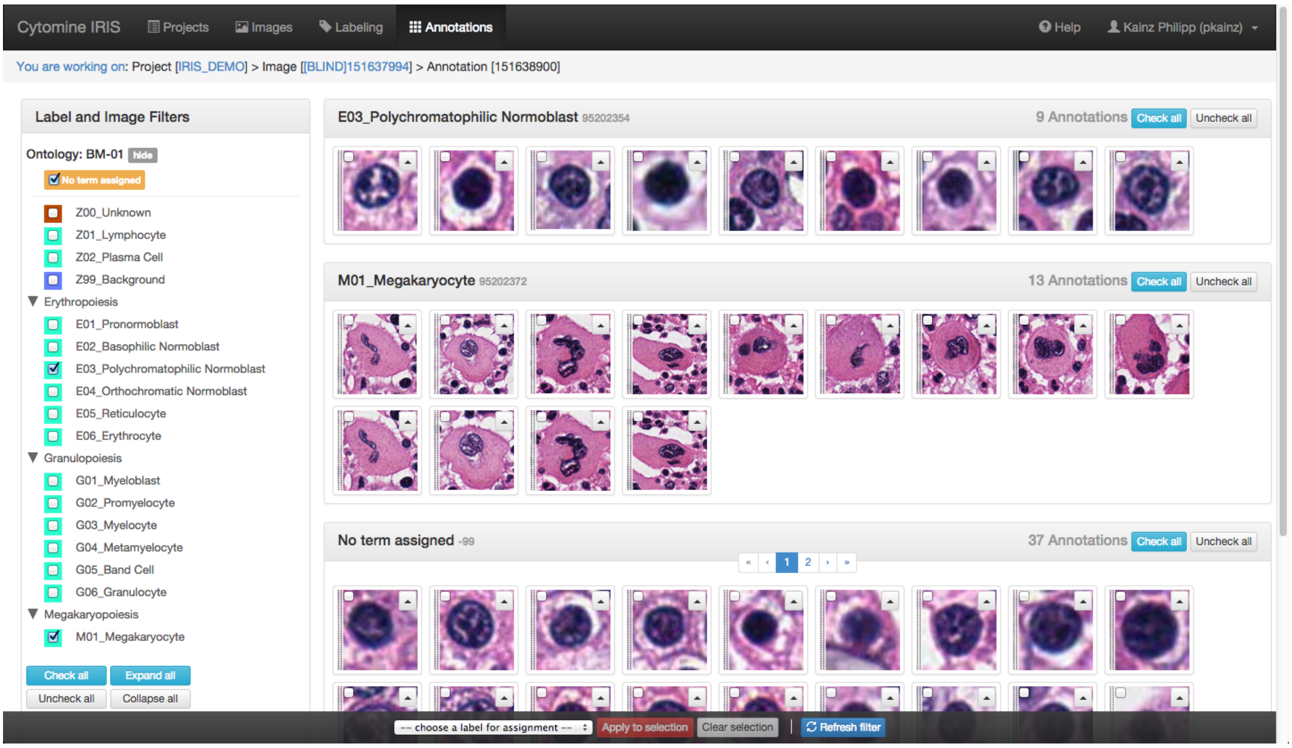

IRIS Sessions

In some scenarios, a single image may contain hundreds of annotations that sometimes cannot be assessed at once. Finding the exact annotation to continue is tedious and thus, IRIS preserves the current status of labeling across logins in sessions, such that the user can continue labeling at the exact same point where the application has been left last time. This can be done by simply going to any IRIS page and clicking on the current image name below the menu bar, or pressing the “r” key on the keyboard, which takes the user back to the labeling view in order to resume the labeling.

Interface Documentation: Help Pages

Each view in IRIS has a dedicated help page that is accessible from the main menu bar (top right corner) or by pressing the “h” key. The pages contain instructions on how to handle the interface.

IRIS Project Coordination

The standard Cytomine user management is extended in IRIS by granting special rights to particular users: the IRIS project coordinators. Each user can request to become a project coordinator and they are authorized once as such by the administrator of the IRIS instance. Coordinators are able to view and visualize project statistics such as histograms of assigned ontology terms for all users or annotation agreements. Each of these visualizations also comes with a variety of filters for particular users, ontology terms and images. Moreover, they can manage project and image access settings for all users and authorize other users to become a project coordinator. Communication among access request and authorization is handled via email, using the email address encoded in the Cytomine User Account page.

Dashboard: User Statistics

The distribution of assigned terms can be evaluated for one or more users in the Statistics Dashboard of each project from the project table (project coordinators). This enables a quick

examination, for example, of whether one or more users are biased towards assigning a particular label. This also gives a clear picture of how many terms have already been assigned by the users with respect to the total number of assigned terms and the total number of available annotations.

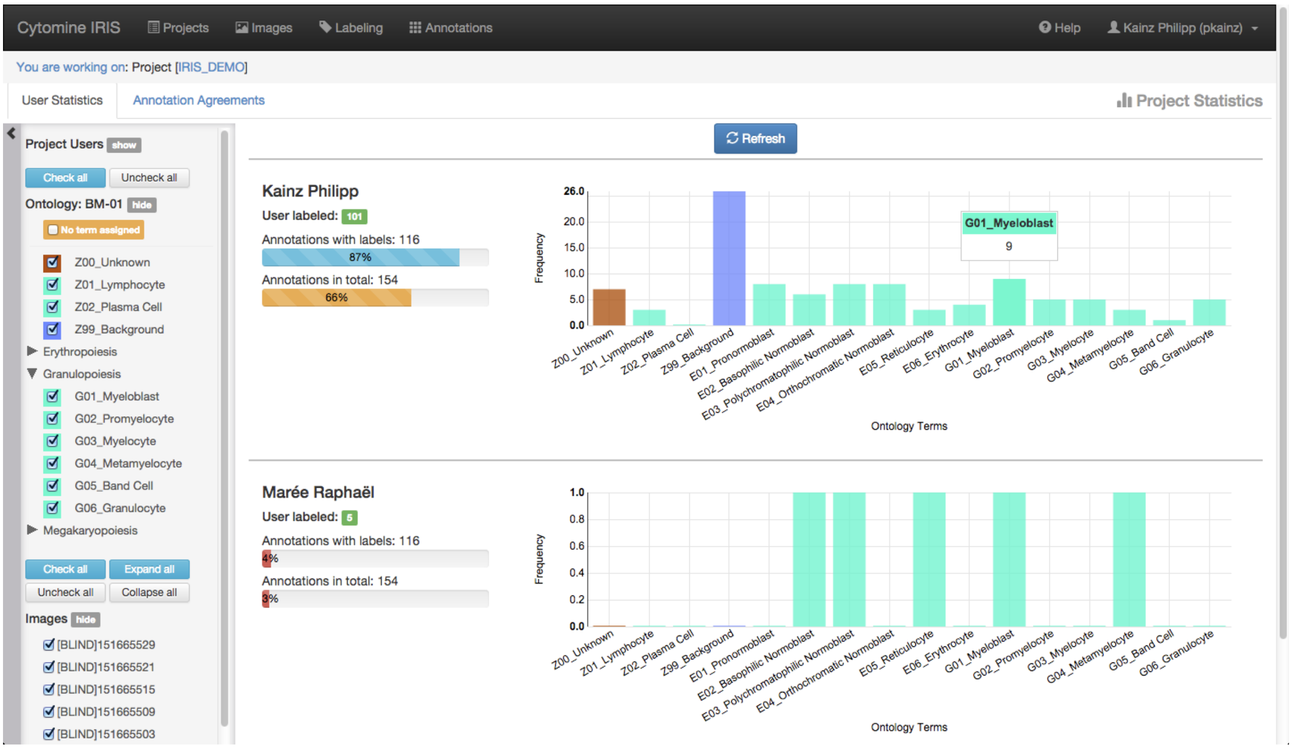

The coordinator can combine several filters (users, ontology terms, images) to meet the current requirements of the query.

Dashboard: Observer Agreements per Annotation

Inter-observer variability of labeled annotations can be visualized in the Statistics Dashboard of a project by selecting the tab titled “Annotation Agreements”.

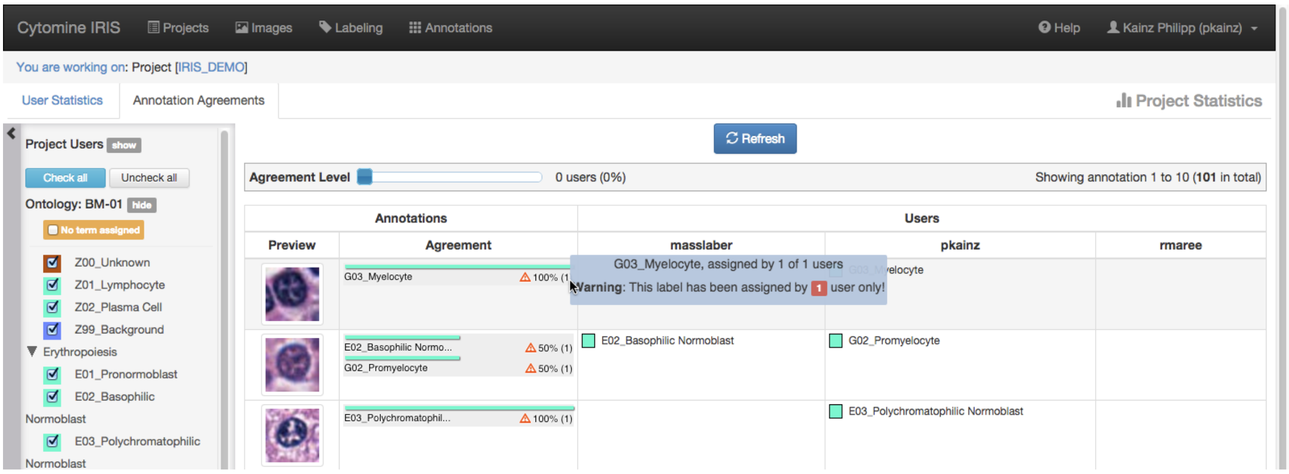

From the filter panel on the left hand side, a coordinator can construct the query and a list of annotations is shown. In addition, this result list can be filtered by annotation agreements using the “Agreement Level” slider at the top of the list. This slider's maximum value corresponds to the total

number of observers that assigned any label in the query. So if the slider is moved to the right, the agreement level increases and filters out annotations, where at least *n* or more observers are agreeing on one or more terms. Moving it to the right usually shrinks the list and hence all annotations in the smaller list have a higher level of inter-observer agreement.

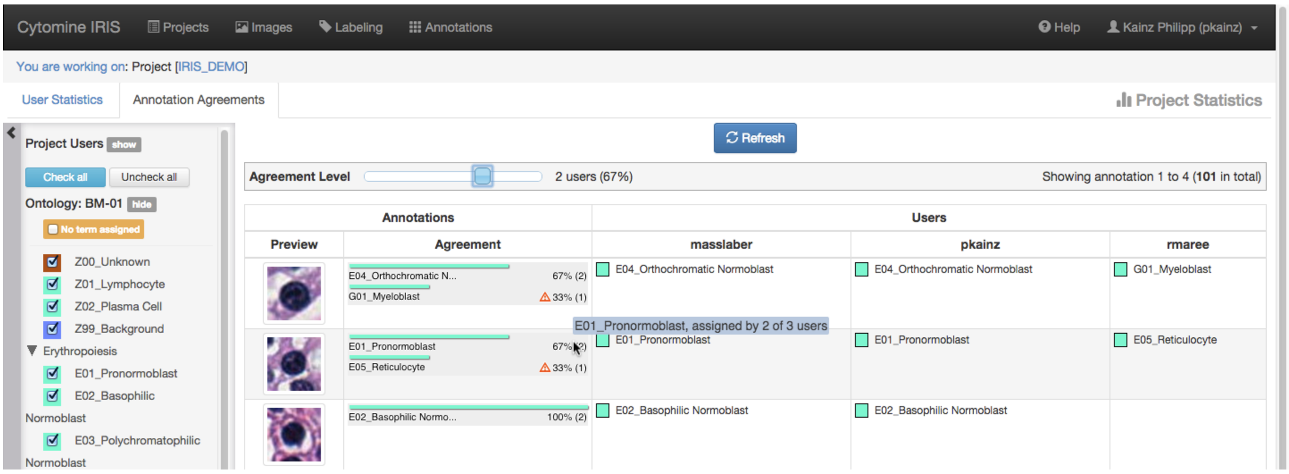

5.3 Usage (API documentation)

Cytomine documentation is available on http://doc.cytomine.be/
A documented RESTful API is continuoulsy updated and accessible online at
http://demo.cytomine.be/restApiDoc/?doc_url=http://demo.cytomine.be/restApiDoc/api#
If you install your own Cytomine instance, this documentation is also automatically installed on your server by our automated installation procedure and then available at:
http://$CORE_URL$/restApiDoc/?doc_url=http://$CORE_URL$/restApiDoc/api#

This API is used both by Cytomine-WebUI (including Cytomine-IRIS) and Cytomine-DataMining analysis modules. It can also be used by third-party software to extend the software for specific purposes. Code examples that encapsulate http requests in Java and Python are provided here:

- https://github.com/cytomine/Cytomine-java-client/tree/master/src/main/java/be/cytomine/client/sample
- https://github.com/cytomine/Cytomine-python-client/tree/master/client/examples

Adding novel softwares and activate them in the Cytomine-WebUI can be done following instructions available from
http://doc.cytomine.be/display/ALGODOC/Extension+with+new+softwares and examples provided here:

- https://github.com/cytomine/Cytomine-java-client/blob/master/src/main/java/be/cytomine/client/sample/SoftwareExample.java

Creating novel web applications communicating with Cytomine components can be done starting from our simple web application template available from: https://github.com/cytomine/Cytomine-sample-webapp

To better understand communication workflows, web users can inspect communications between their web browser and Cytomine components through the "Network" tab in the "Inspect Element"

(Google Chrome)/"Inspector" (Mozilla Firefox) module of their web browser:

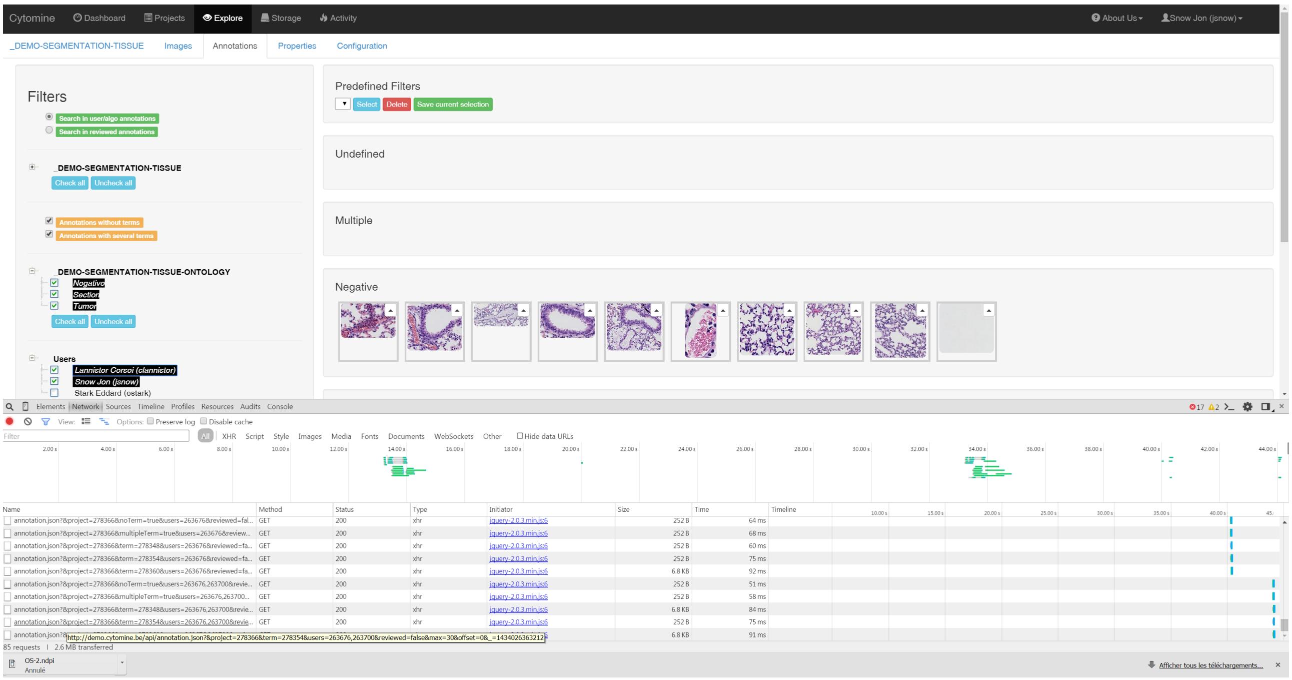

In addition, the API online documentation includes a "Playground" for each web service that allows the user to test server responses according to user input (e.g. here we use the service to get annotation description given an annotation identifier):

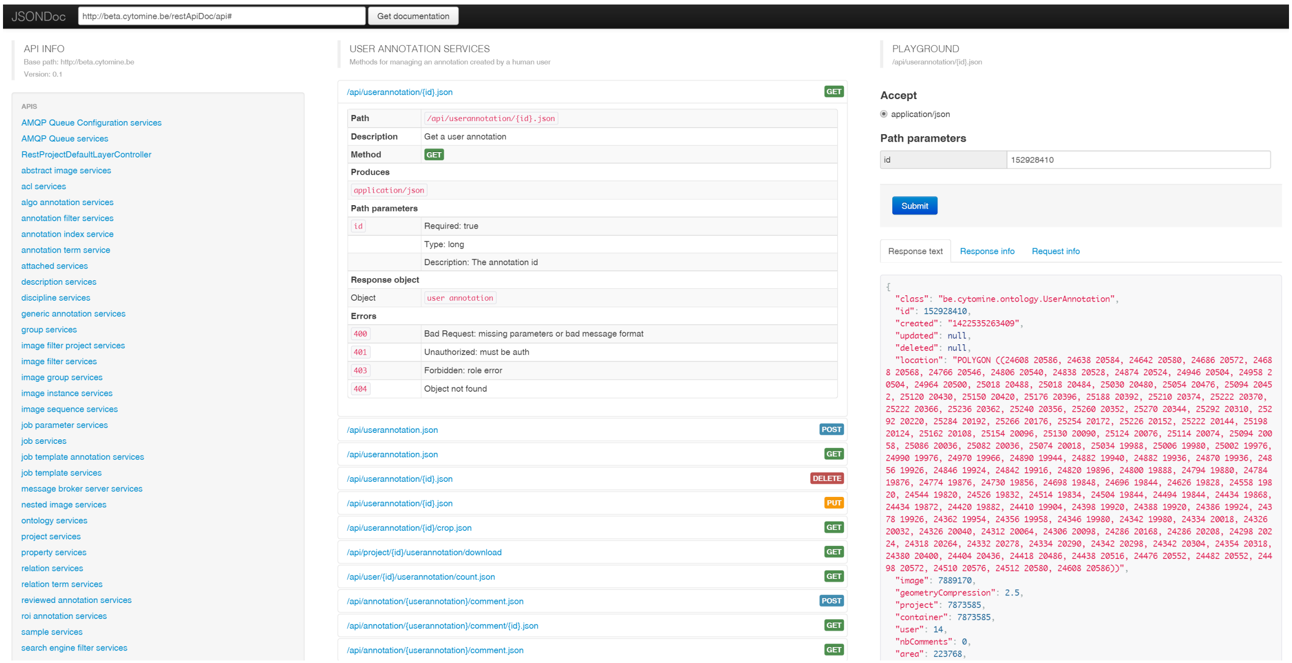

The following table give some examples of URLs to export data (e.g. using jsnow username account on demo.cytomine.be):

**URL example**	**Description**
http://demo.cytomine.be/api/user.json	List of users (JSON)
http://demo.cytomine.be/api/user/263676.json	Description of a specific user (JSON)
http://demo.cytomine.be/api/project.json	List of projects (JSON)
http://demo.cytomine.be/api/project/528050.json	Descrpition of a specific project (JSON)
http://demo.cytomine.be/api/project/528050/imageinstance.json	List of images in a specific project (JSON)
http://demo.cytomine.be/api/imageinstance/528460.json	Description of a specific image (JSON)
http://demo.cytomine.be/api/abstractimage/528120/thumb.png?maxSize=1024	Thumbnail of a specific image (PNG)
http://demo.cytomine.be/api/annotation.json?&project=528050	List of annotations in a specific project (JSON)
http://demo.cytomine.be/api/annotation.json?&project=528050&term=528044&users=263676&images=528132	List of annotations of a specific ontology term, in a specific project, for a specific user (human or userjob), in a specific image (JSON)
http://demo.cytomine.be/api/annotation/528401.json	Description of a specific annotation (JSON)
http://demo.cytomine.be/api/userannotation/528401/crop.png	Crop image of a specific annotation (PNG)
http://demo.cytomine.be/api/userannotation/528401/crop.png?increaseArea=2	Crop image of a specific annotation (PNG) with context
http://demo.cytomine.be/api/userannotation/528401/crop.png?zoom=2	Crop image of a specific annotation at zoom level 2 (PNG)
http://demo.cytomine.be/api/userannotation/528401/crop.png?zoom=2&mask=true	Crop binary mask of a specific annotation at zoom level 2 (PNG)
http://demo.cytomine.be/api/userannotation/528401/crop.png?zoom=2&alphaMask=true	Crop alpha mask of a specific annotation at zoom level 2 (PNG)
http://demo.cytomine.be/api/imageinstance/528460/window-23181-14285-1000-1000.png	Tile of size 1000x1000 at a specific location (23181,14285) in a specific image (PNG)
http://demo.cytomine.be/api/imageinstance/528460/window-23181-14285-1000-1000.png?mask=true&review=true&terms=528044	Tile reviewed mask of size 1000x1000 at a specific location (23181,14285) for a specific ontogy term in a specific image (PNG)
http://demo.cytomine.be/api/job/1939560.json	Description of a specific job (JSON) including software parameter values

Supplementary References

1. Pietzsch, T., Saalfeld, S. Preibisch, S., Tomancak, P. Nature Methods 12, 481–483. (2015)
2. Saalfeld, S., Cardona, A., Hartenstein, V., and Tomancak, P. Bioinformatics **25**(15), pp. 1984–1986. (2009)
3. Carpenter AE, Jones TR, Lamprecht MR, Clarke C, Kang IH, Friman O, Guertin DA, Chang JH, Lindquist RA, Moffat J, Golland P, Sabatini DM. Genome Biology 7:R100. (2006)
4. Schindelin, J. Arganda-Carreras, I. Frise, E., Kaynig, V., Longair, M., Pietzsch, T. Preibisch, S., Rueden, C., Saalfeld, S., Schmid, B. Tinevez, J.-Y., James White, D., Hartenstein, V., Eliceiri,K., Tomancak, P., Cardona. A. Nature Methods 9, 676–682 (2012)
5. de Chaumont, F.Dallongeville, S., Chenouard, N. ,Hervé, N., Pop, S. , Provoost, T.,Meas-Yedid, V., Pankajakshan,P., Lecomte, T., Le Montagner Yoann,Lagache, T., Dufour A., Olivo-Marin, J.-C.,. Nature Methods 9, 690–696. (2012)
6. Sommer, C., Strähle, C., Köthe, U., Hamprecht, F.A. in Proc. IEEE ISBI, 230–233. (2011).
7. Allan, C., Burel, J-M., Moore, J., Blackburn, C., Linkert, M., Loynton, S., MacDonald, D., J Moore, W., Neves, C., Patterson, A., Porter, M, Tarkowska, A., Loranger, B., Avondo, J., Lagerstedt, I., Lianas, L., Leo, S., Hands, K., T Hay, R., Patwardhan, A., Best, C. J Kleywegt, G., Zanetti, G. & Swedlow, J. Nature Methods 9, 245–253. (2012)
8. Zsolt L. Husz, Thomas P. Perry, Bill Hill, Richard A. Baldock. Advances in Visual Computing, Lecture Notes in Computer Science Volume 5875, pp 924-933, (2009)
9. Rübel, O., Greiner, A., Cholia, S., Louie, K., E. Wes Bethel, R. Northen, T., Bowen, B.. Anal. Chem., 85 (21), pp 10354–10361 (2013)
10. Marée, R., Denis, P., Wehenkel, L., Geurts, P., Proc. ACM MIR, pp. 91-100 (2010).
11. Marée, R., Wehenkel, L., Geurts, P., Invited chapter in Decision Forests in Computer Vision and Medical Image Analysis, Advances in Computer Vision and Pattern Recognition, Criminisi, A; Shotton, J (Eds.), pp 125-141. (2013)
12. Marée, R., Geurts, P., Wehenkel, L. Technical Report, University of Liege http://hdl.handle.net/2268/175525 (2014)
13. Marée, R., Geurts, P., Piater, J., Wehenkel, L. Proc. CVPR (1):34-40. (2005)
14. Marée, R., Geurts, P., Wehenkel, L., IPSJ Transactions on Computer Vision and Applications, Information Processing Society of Japan, (2009).
15. Moosmann, F., Nowak, E., Jurie, F., IEEE Transactions on Pattern Analysis and Machine Intelligence. 30(9):1632-1646. (2008)
16. Leung, T., Malik, J., International Journal of Computer Vision 43(1):29-44. (2001)
17. Sivic, J., Zisserman, A., Proc. ICCV (2):1470-1477. (2003)
18. Dance, C., Willamowski, J., Fan, L., Bray, C., Csurka, G. Proc. ECCV Workhop on Statistical Learning in Computer Vision. (2004)
19. Dumont, M., Marée, R., Wehenkel, L., Geurts, P., Proc. VISAPP(2):196-203. (2009)
20. Stern, O., Marée, R., Aceto, J., Jeanray, N., Muller, M., Wehenkel, L., Geurts, P. Proc. IAPR International Conference on Pattern Recognition in Bioinformatics (7036):179-190. (2011)
21. Murphy, RF. Nature Chemical Biology 7, 327–330 (2011)
22. Danuser, G. Cell 147(5):973-978 (2011)
23. de Souza, N. Nature Methods 9(10): 38 (2013)
24. Liberali, P., Pelkmans, L. Nature Cell Biology 14(12): 1233. (2012)
25. Fuhai, L., Zheng, F., Guangxu, J., Zhao, H., Wong, S., Stephen T.C., PloS Comput Bio. 9(4): e1003043 (2013).
26. Marée, R., Rollus, L., Stevens, B., Louppe, G., Caubo, O., Rocks, N. Bekaert, S., Cataldo, D., Wehenkel, L. Proc. IEEE ISBI: 902-906 (2014)
27. Wang et al., IEEE Trans Med Imaging. Epub doi:10.1109/TMI.2015.2412951 (2015)
28. Vandaele, R., Marée, R., Jodogne, S., Geurts, P. Technical Report, University of Liege, (2015) http://hdl.handle.net/2268/182930
29. Marée, R., Stevens, B., Rollus, L., Rocks, N., Moles Lopez, X., Salmon, I., Cataldo, D., Wehenkel, L., Diagnos Pathol 8 (S1):S26 (2013)
